# Comparing land surface phenology of major European crops as derived from SAR and multispectral data of Sentinel-1 and -2

**DOI:** 10.1016/j.rse.2020.112232

**Published:** 2021-02

**Authors:** Michele Meroni, Raphaël d'Andrimont, Anton Vrieling, Dominique Fasbender, Guido Lemoine, Felix Rembold, Lorenzo Seguini, Astrid Verhegghen

**Affiliations:** a European Commission, Joint Research Centre (JRC), Via E. Fermi 2749, I-21027 Ispra, VA, Italy; b University of Twente, Faculty of Geo-information Science and Earth Observation, P.O. Box 217, 7500, AE, Enschede, the Netherlands

**Keywords:** Satellite image time series, Land surface phenology, Agriculture, Crop phenology, Sentinel-1, Sentinel-2, LUCAS survey, DWD ground phenological observations, Europe

## Abstract

The frequent acquisitions of fine spatial resolution imagery (10 m) offered by recent multispectral satellite missions, including Sentinel-2, can resolve single agricultural fields and thus provide crop-specific phenology metrics, a crucial information for crop monitoring. However, effective phenology retrieval may still be hampered by significant cloud cover. Synthetic aperture radar (SAR) observations are not restricted by weather conditions, and Sentinel-1 thus ensures more frequent observations of the land surface. However, these data have not been systematically exploited for phenology retrieval so far. In this study, we extracted crop-specific land surface phenology (LSP) from Sentinel-1 and Sentinel-2 of major European crops (common and durum wheat, barley, maize, oats, rape and turnip rape, sugar beet, sunflower, and dry pulses) using ground-truth information from the “Copernicus module” of the Land Use/Cover Area frame statistical Survey (LUCAS) of 2018. We consistently used a single model-fit approach to retrieve LSP metrics on temporal profiles of CR (Cross Ratio, the ratio of the backscattering coefficient VH/VV from Sentinel-1) and NDVI (Normalized Difference Vegetation Index from Sentinel-2). Our analysis revealed that LSP retrievals from Sentinel-1 are comparable to those of Sentinel-2, particularly for winter crops. The start of season (SOS) timings, as derived from Sentinel-1 and -2, are significantly correlated (average r of 0.78 for winter and 0.46 for summer crops). The correlation is lower for end of season retrievals (EOS, r of 0.62 and 0.34). Agreement between LSP derived from Sentinel-1 and -2 varies among crop types, ranging from *r* = 0.89 and mean absolute error MAE = 10 days (SOS of dry pulses) to *r* = 0.15 and MAE = 53 days (EOS of sugar beet). Observed deviations revealed that Sentinel-1 and -2 LSP retrievals can be complementary; for example for winter crops we found that SAR detected the start of the spring growth while multispectral data is sensitive to the vegetative growth before and during winter. To test if our results correspond reasonably to in-situ data, we compared average crop-specific LSP for Germany to average phenology from ground phenological observations of 2018 gathered from the German Meteorological Service (DWD). Our study demonstrated that both Sentinel-1 and -2 can provide relevant and at times complementary LSP information at field- and crop-level.

## Highlights

• Crop-specific phenology retrieved from Senetinel-1 and Sentinel-2 time series.
• European Union wide survey LUCAS exploited to focus on major European crops.
• Comparable phenology from cross polarization ratio VH/VV and from Sentinel-2 NDVI.
• Retrieved phenology consistent with ground phenological observation from DWD.
• Sentinel-1 and -2 provide relevant and at times complementary LSP information.

## 1 Introduction

Phenology can be defined as the timing of biological events. Plant phenology directly controls vegetation productivity by mediating carbon, water, and energy fluxes (Richardson et al. 2013). Crop phenological information is crucial for yield modelling and crop monitoring, because the impact of weather conditions on the final crop yield depends on the crop phenological stage (Ceglar et al. 2019; Sakamoto et al. 2013). Crop phenology provides important information for crop-management, for example for efficient irrigation, fertilization, pest management (Gao et al. 2017), and post-harvest management (Stathers et al. 2013). Phenology is also used to monitor the crop progress in food security monitoring applications (Rembold et al. 2019) and can represent a key indicator for the control of the European Common Agricultural Policy (CAP, Devos et al. 2018), allowing a first verification of the farmers' crop declaration by comparing the expected timing of growing season with the satellite-derived one.

Crop growth stages are conventionally identified by ground-based monitoring activities. These activities provide accurate description of crop phenology but are also highly time- and resource-consuming and thus seldom put in place at large scale. For instance, at present no ground survey exists at the European level for estimating crop phenology. Remote sensing data can support crop growth monitoring at scale by providing timely information on crop health status and phenological development. Multi-temporal remote sensing data can be used to derive descriptors of land surface phenology (LSP), the spatio-temporal development of the vegetated land surface (De Beurs and Henebry 2005). Examples of such descriptors are the start of greening/season (SOS) and the onset of senescence or end of season (EOS). Over croplands, LSP can inform about management practices (e.g. sowing, harvesting), crop type groups (e.g. summer and winter crops) and has been used to estimate crop yield (Bolton and Friedl 2013; Kucharik 2006; López-Lozano et al. 2015).

Moderate to coarse resolution observations (250–1000 m) from different multispectral satellite missions (e.g. the Advanced Very High Resolution radiometer, AVHRR; the Moderate Resolution Imaging Spectrometer, MODIS; the Satellite Pour l'Observation de la Terre - Vegetation, SPOT-VGT) have been frequently used to derive LSP (e.g. De Beurs and Henebry 2005; Jönsson and Eklundh 2002; White et al. 1997; Zhang et al. 2003), because they offer adequate temporal resolution (i.e. daily overpass of the sensors). A recent review of phenology retrieval methods and instruments used can be found in Zeng et al. (2020).

The higher spatial resolution from Landsat and Sentinel-2 satellites has opened the possibility of moving closer to the phenology of specific plant species and, as such, may provide a stronger link to ground-observed data (Fisher et al. 2006). This is particularly relevant for crop phenology that can vary substantially among neighbouring fields because of different crops grown, cultivars and management practices (e.g. sowing dates, harvesting time, irrigation and fertilization), in contrast to the phenology of natural vegetation species that is less spatially heterogeneous because mostly driven by soil, topographic conditions and climate (Kimball 2014).

Fine spatial resolution LSP (e.g. 30 m) has been extracted from Landsat imagery (Melaas et al., 2013, Melaas et al., 2016). The 10–30 m resolution offered by recent multispectral satellite missions, including Sentinel-2, can resolve single agricultural fields and thus estimate field- and crop- specific phenology. Recently, multiple LSP retrieval efforts used data from the Sentinel-2 satellites and harmonized Landsat 8 and Sentinel-2 imagery (Bolton et al. 2020; Fan et al. 2020; Jönsson et al. 2018; Vrieling et al. 2018). Sentinel-2A and 2B satellites (S2 in the following) provide multispectral imagery at 10 to 60 m resolution and a five-day revisit interval at the equator. This interval is shorter towards the poles where overlap between orbits increases. However, despite S2's short revisit time, effective phenology retrieval may still be hampered by significant cloud cover reducing the actual availability of valid observations (Cheng et al. 2020; Jönsson et al. 2018).

Synthetic Aperture Radar (SAR) observations such as those provided by Sentinel-1 are not restricted by weather conditions, and Sentinel-1A and 1B satellites (S1 in the following) thus ensure land surface observations every six days at the equator for single orbit direction (ascending or descending) or more frequent towards the poles and/or when both orbit directions are combined. This is a key advantage for phenology retrieval because it provides a dense temporal sampling throughout the season, which potentially allows to describe the vegetation seasonal development with greater detail, as compared to current multispectralobservations at equivalent spatial resolution. However, whereas the research field of LSP retrieval from multispectral data has been very active in the last decades (Zeng et al. 2020), only few examples of the use of SAR data for this purpose are available.

S1 provides dual polarization (VV and VH) backscatter coefficients in the C-band resampled at 10 × 10 m ground pixel spacing in the Interferometric Wide (IW) swath mode. The SAR response of C-band sensors is related to vegetation biomass, structure, and soil conditions. The backscatter from vegetation has been studied since the late 1970s (Ulaby et al. 1982) and is largely dictated by the leaf structure, leaf orientation, and canopy water content. Simplified models (Attema and Ulaby, 1978) which are derived from more elaborate electromagnetic scattering models (Ulaby et al. 1990), describe backscattering from crop canopies as that from a layered water cloud overlaying a soil surface. The backscatter of the soil surface is modulated by the soil material properties and its moisture content, which together determine the dielectric properties, and by the surface geometry (Fung and Chen, 2004). Polarimetric SAR (PolSAR) measurements have been used to detect crop growth stages, often of rice, treating phenology retrieval as a classification task where stages are regarded as separate classes and identified by a static or dynamic classification algorithm trained for a specific crop and area (Bouvet et al. 2009; Mascolo et al. 2016). McNairn et al. (2018) used multi-year RADARSAT-2 quad polarization and TerraSAR-X dual polarization SAR data with a dynamic filtering framework and field observations to estimate canola growth stages. The introduction of freely accessible Sentinel-1 data has greatly stimulated a renewed interest in studying the backscattering of vegetation. Sentinel-1 provides consistent access to calibrated backscattering parameters at a frequency that matches the dynamics of the crop phenology cycle. Stendardi et al. (2019) studied S1 VH backscatter in relation to NDVI (Normalized Difference Vegetation Index) from S2 in alpine meadows and found the retrieved LSP comparable when extracted independently from both sources. Khabbazan et al. (2019) found that temporal variations in backscatter data reflect changes in soil water content and plant structure associated with phenological development for crops in the Netherlands. Veloso et al. (2017) studied the temporal behaviour of S1 backscatter coefficients and S2 NDVI of various crop types and suggested that the backscatter coefficient ratio VH/VV (cross polarization ratio, CR) may be applied in a similar fashion to NDVI for mapping and monitoring crops.Vreugdenhil et al., 2018 suggested the use of CR for crop monitoring as it minimises the effect of soil moisture and soil-vegetation interaction effects. Nasrallah et al. (2019) detected wheat germination and harvesting dates in selected fields in Lebanon with CR (VV/VH in their work), while the use of VV and VH backscatter coefficients was preferred for the heading and soft dough dates, respectively. Likewise, Schlund and Erasmi (2020) showed the potential of CR for detecting the date of shooting and harvesting of selected wheat fields in Germany. All these studies recognised that more research is needed to clarify the usefulness of CR because changes in soil roughness and vegetation structure also affect its temporal behaviour to some extent. Mercier et al. (2020) evaluated the potential of Sentinel-1 and -2 data to retrieve wheat and rapeseed phenological stages with a classification approach over a limited set of fields in northern France. The study showed that the combined use of input features from both sensors improved the accuracy in the classification as compared to the use of each sensor alone.

At present, the potential of S1 data for crop-specific phenology has not been investigated systematically over a range of different crops and environmental conditions. Capitalising on the availability of geolocated crop type information from the European Union (EU) wide ground Land Use and Coverage Area frame Survey (LUCAS), this manuscript aims at filling this gap by providing a first comparison between the phenology obtained from S1 and S2 of major European crops. A plausibility check for the retrieved LSP is made using ground-based phenological observations of crops in Germany obtained from the German Meteorological Service (DWD).

Specifically, the goals of this paper are:

1. to retrieve LSP of main European crops from 10 m resolution optical S2, as well as from SAR S1 data;
2. to compare LSP retrievals from S1 and S2 over a large area and diverse agro-ecological conditions;
3. to provide a first consistency evaluation between retrieved LSP and ground-based phenology observations in Germany.

The paper is organised as follows. Section 2 provides information on the study area and on the data used, including the ground surveys and the satellite data. Section 3 describes the pre-processing of satellite data and the algorithm used to extract LSP. Results of crop-specific S1 and S2 LSP estimates are presented in Section 4 and discussed in Section 5. Finally, conclusions and outlook are reported in Section 6.

## 2 Study area and data

The study is executed over the EU for the year 2018, using data from the LUCAS Survey (https://ec.europa.eu/eurostat/web/lucas). It is noted that 2018 was a peculiar year, with droughts and severe heatwaves affecting Europe during summer. Drought conditions in central and northern Europe caused yield reductions up to 50% for the main crops, yet wet conditions in southern Europe saw yield gains up to 34%, both with respect to the previous five-year mean (Toreti et al. 2019).

### 2.1 LUCAS-Copernicus

The LUCAS survey, carried out by EUROSTAT on a three-yearly basis since 2006, focuses on the state and the dynamics of changes in land use and cover in the EU. The survey is a land cover and land use standardized data collection exercise that extends over the whole of the EU's territory every three years with the main objective of providing accurate EU-level land use/cover statistical estimates. The survey is carried out mainly in-situ with a large number of ground observations collected throughout the EU covering all land covers, which include artificial land, cropland, woodland, shrubland, grassland, bareland, water, and wetlands. A comprehensive description of LUCAS campaigns (years 2006, 2009, 2012, 2015 and 2018) can be found in d'Andrimont et al. (2020a).

In 2018, a new component was included in the LUCAS survey, referred to as the “Copernicus module” from the name of the EU's Earth Observation Programme, Copernicus. For 90,557 points (a fraction of the 337,031 LUCAS points collected in 2018), a specific protocol was applied to collect additional in-situ information that could serve as ground-truth for Earth Observation analysis. As part of this protocol, the geographic location of classical LUCAS points is assigned to a surveyor, who in the case of croplands needs to classify the crop type and ascertain that the same crop type is present in a 1.5 m radius around that point. The surveyor should be able to observe the point and reliably classify it, being exactly at the point location or nearby. This classical LUCAS observation is valid only for a fraction of the 10 m Sentinel (−1 and − 2) pixel, i.e. the circle with 1.5 m radius (representing thus an area of 7.07 m^2^), and it is thus not directly usable with such decametric sensors. In fact, the 10 m pixel (i.e. 100 m^2^) could be covered by different land covers while the LUCAS observation only captures one. The exact geolocation of the surveyor observation is recorded only in the corresponding LUCAS-Copernicus entry, along with information on the spatial extent of the observed land cover and neighbouring land covers within 50 m from the recorded point (51 m is recorded if the land cover is homogeneous for an extent larger than 50 m) in the four cardinal directions (N, E, S, W). Thus, although typically close each other, the nominal geolocation of a LUCAS point may not exactly overlap with that of a LUCAS-Copernicus point.

In order to obtain areas with homogeneous land cover and useful for remote sensing application, information from both LUCAS and LUCAS-Copernicus points was gathered and filtered to build polygon geometries as fully described in d'Andrimont et al. (2020b) and briefly reported hereafter. First, for each of the LUCAS-Copernicus points, a polygon with unique crop type cover was delineated using the surveyor's information about land cover type in the four cardinal directions, i.e. the four distances in the cardinal directions provided by the surveyor are added to the coordinates of the point to build a polygon (i.e. an irregular quadrilateral). The quadrilateral diagonals can measure up to 102 m (0.52 ha, equivalent to 52 pixels with 10 m resolution), but are less in case the surveyor found a field boundary within 50 m of the LUCAS-Copernicus point. Second, as we are interested in crop type information (LUCAS legend level-3), we had to gather it from the LUCAS classical points because LUCAS-Copernicus points only record the crop group (legend level-2). For this match, we retained only those records where the LUCAS nominal geolocation falls within the LUCAS-Copernicus polygon and the identified crop type covered a homogeneous area estimated to be larger than 0.1 ha.

As a result, 6095 LUCAS-Copernicus cropland polygons were retained. Here, we limited the analysis to all non-forage crops with more than 100 records in the LUCAS-Copernicus database. These comprise the following nine crops: common wheat, barley, maize, durum wheat, oats, rape and turnip rape, sugar beet, sunflower, and dry pulses. These nine crops comprise the most cultivated non-forage crops in the EU (Eurostat, 2018). We note that LUCAS-Copernicus does not distinguish between winter and spring varieties of barley and wheat. Due to the further focusing on only nine crops, the final sample size used in this study amounts to 4659 polygons (average area = 0.37 ha, SD = 0.13 ha; crop-specific sample size in Table 2). These polygons were then used as regions of interest to extract satellite time series.

### 2.2 Ground phenological observations

A dataset of ground phenological observation for Germany during year 2018 was obtained from the German Meteorological Service (Deutscher Wetterdienst - DWD). DWD maintains a database of phenological observations that covers a large number of years and plants, including various crop species. The phenological observations are collected by volunteer surveyors at specific observation locations (so-called stations) throughout the agricultural season. The observations take place two or three times per week, over the same field that is located within a distance of 5 km from the nominal geolocation associated to the station (DWD 2015); precise field locations are not available but the station locations are. For each crop, a predefined set of phenological stages is recorded. A specific phenological stage is deemed to occur when more than 50% of the plants in the field have reached that stage. Surveyors report the estimates of crop phenological stage as assessed qualitatively by visual observation.

The phenological stages are defined according to a specific DWD-code with associated description. A lookup table between DWD scale and the more widely used BBCH scale is provided with the data. BBCH is a numeric classification system (Meier 2001) where phenological stages are subdivided in principal (e.g. germination, leaf development, etc.) and secondary stages (short developmental steps which are passed successively during the respective principal growth stage). These principal and secondary stages are represented in the BBCH code as the first and second digit.

Data and the full data description are freely available at the DWD Climate Data Centre (CDC, ftp://ftp-cdc.dwd.de). From the full database we used the available crop types that matched the ones investigated in the present study: i.e. wheat, barley, maize, rapeseed, oats, and sugar beet. We then retained the observations checked as reliable according to DWD quality flags (Kaspar et al. 2015a; Kaspar et al. 2015b). A direct match between LUCAS-Copernicus and DWD crop type was established for all crops except barley. In fact, contrary to LUCAS-Copernicus, DWD distinguishes between winter and spring varieties of barley. We coupled both DWD barley varieties with the same LUCAS barley class. For the DWD-code 24 (harvest) no BBCH association was provided, but we associated it with the BBCH code 99 (harvested product) for all the crops.

The number of sample observations varies per crop and per phenological stage. Table 1 provides a complete description of sample availability and phenological stage description. As shown in Table 1, the number of DWD samples is in all cases larger than the number of LUCAS-Copernicus polygons available in Germany (the number of successful LSP retrieval is reported).

**Table 1:** Number of DWD ground observations and LUCAS-Copernicus polygons with successful LSP retrievals in Germany. Stage description and corresponding BBCH code is reported for DWD. The number of ground observations varies according to the stage observed due to missing observations for some of the stations. Note that the DWD crop type rapeseed was associated to the LUCAS crop type rape and turnip rape.

			Sample size (n)						
Data set	Description	BBCH	Wheat	Winter barley	Spring barley	Maize	Rapeseed	Oats	Sugar beet
DWD									
	Sowing	0	613	590	392	636	502	389	254
	Early leaf development	10	592	585	390	654	488	376	256
	Later leaf development	14					416		
	Early stem elongation	31	576	571	352	571	490	331	
	Rosette growth (50% cover)	35							255
	Early heading	51	612	619	346		550	357	
	Early inflorescence, heading	53				592			
	Early flowering	61				586	642		
	Grain development	75	524			501		311	
	Early ripening	83				435			
	Late ripening	87	567	581	339	364		353	
	Fully ripe	89					519		
	Harvest	99	650	654	375	623	586	384	290
LSP	NDVI-derived		247	97	97	65	29	10	25
	CR-derived		251	99	99	68	31	10	27

### 2.3 Sentinel data

We extracted Sentinel-1 and -2 data for the LUCAS-Copernicus polygons from Google Earth Engine (GEE). For S2 we used Level-1C imagery, top of atmosphere (TOA) reflectances from the GEE collection “COPERNICUS/S2”. TOA reflectances were used because top of canopy reflectance products (Level-2A) over the full EU were in the process being ingested in the GEE system and available only since 2019 at the time of our analysis, thus not covering the period of interest.

For S1 we used data acquired in the Interferometric Wide (IW) swath mode from both ascending and descending orbits, providing dual-polarization (VV and VH) imagery at 10 × 10 m pixel spacing. Data were gathered from the GEE collection “COPERNICUS/S1_GRD_FLOAT” that includes the Ground Range Detected (GRD) scenes, processed using the Sentinel-1 Toolbox to remove thermal noise and generate a calibrated, ortho-corrected raw (i.e. no dB scaling applied) backscatter coefficient σ^0^. All available observations of Sentinel-1 and -2 between 1 August 2017 and 31 March 2019 were downloaded to guarantee that a full phenological cycle could be covered for all crops investigated.

## 3 Methods

### 3.1 Processing of Sentinel data

Cloud, cloud shadow, and snow screening of S2 data was implemented in GEE using a simple cloud- and snow-score algorithm adapted from the one developed by Housman et al. (2018) for Landsat 8. Briefly, the algorithm assigns a higher cloud-score to pixels with: higher brightness in the blue and cirrus bands (S2 bands 1, 2 and 10), higher brightness in all visible bands (bands 2 to 4), and higher Normalized Difference Water Index (NDWI, Gao 1996; calculated with bands 8 and 11). Shadows are detected by assessing the correspondence between dark pixels and the cloud ground projection made using actual solar geometry at time of overpass and nominal cloud height. Snow-score is assigned using the Normalized Difference Snow Index (NDSI, Hall et al. 2002) formed by the green and SWIR (bands 3 and 11), in addition to the brightness indicators described for the cloud-score. Finally, the Normalized Difference Vegetation Index (NDVI) is computed using bands 4 and 8 at 10 m spatial resolution.

Both NDVI and CR are calculated at the pixel level in GEE. The average NDVI and CR was then calculated for each polygon delineated as described in Section 2.1 by weighing the pixel value by the fraction of the pixel that intersects the LUCAS-Copernicus polygon. In this way, pixels at the border of the homogeneous polygon (with potentially a different land cover or crop type) are assigned a lower weight. In addition, to reduce possible contamination due to edge effects we reduced the quadrilateral dimension using a one-meter internal buffer. We did not employ a larger buffer to avoid larger reduction of the sample area (and number of pixels). Nevertheless, despite both S1 and S2 have a pixel spacing of 10 m, we recognise that the edge effect on polygon-level averages may be larger for S1 as compared to S2 due to the coarser resolution of S1 (22 m in azimuth and 2.7 m in near range and 3.5 m in far range for single-look-complex imagery).

For NDVI, the average was extracted from cloud and snow free pixels inside the polygon, and in addition the standard deviation was calculated. While the standard deviation of NDVI informs about land surface heterogeneity within the polygon, the standard deviation of the radar backscatter coefficient is strongly influenced by speckle (i.e. due to constructive and destructive interference of waves), and for a homogeneous target is proportional to its mean (Touzi 2002), i.e. the standard deviation equals the mean divided by the square root of the so-called equivalent number of looks (ENL, being about 4 for S1 GRD data). For this reason, we do not show the within-polygon standard deviation of the S1 data in this paper, as it would relate more to the expected signal than to field heterogeneity.

The total number of pixels per polygon and the number of valid pixels are retrieved. Temporal profiles are then downloaded locally for further processing. A description of the available samples is presented in Table 2.

**Table 2:** Crops considered in this study: definition, total number of polygons, statistics of the number of valid satellite observations available in the period 1 August 2017 and 31 March 2019, and crop group (either winter crops or summer crops).

	LUCAS		Polygons (n)	S2 obs. (n)			S1 obs. (n)			Crop group
Crop	Code	Definition		Avg	Min	Max	Avg	Min	Max	
Common wheat	B11	*Triticum aestivum*, spring or winter wheat	1899	78	27	184	328	162	474	winter
Barley	B13	*Hordeum vulgare*	1028	85	24	183	318	162	490	winter
Maize	B16	*Zea mays*	562	78	28	181	325	165	442	summer
Durum wheat	B12	*Triticum durum*	313	97	36	180	302	165	404	winter
Rape and turnip rape	B32	*Brassica napus* and *Brassica rapa* var. *oleifera*	238	73	28	163	338	165	426	winter
Oats	B15	*Avena sativa*	219	85	29	186	325	169	426	winter
Sugar beet	B22	*Beta vulgaris* var. *altissima*	155	67	31	140	346	195	528	summer
Sunflower	B31	*Helianthus annuus*	123	93	46	169	303	166	404	summer
Dry pulses	B41	Dry peas (*Pisum sativum*), Chickpea (*Cicer arietinum*), Cowpea (*Vigna sinensis*; *Dolichos sinensis*), Pigeon pea (*Cajanus cajan*), Field peas (*Pisum arvense*), Field beans (*Vicia faba spp.),* Lentils (*Lens culinaris*), Lentil vetches (*Vicia ervilia*), Vetches, spring or common vetch (*Vicia sativa, Vicia villosa*), Lupins (*Lupinus spp.*), Peanuts (*Arachis hypogaea*)	122	89	30	177	318	165	445	winter

Although NDVI polygon-level averages are computed on cloud-screened pixels only, we did further screening to avoid possible undesired contaminations. We discarded S2 NDVI averages that were computed when the cloud screening detected 5% or more cloud-affected pixels inside the polygon, indicating close proximity of clouds. Then, to avoid that snow melting, associated with a sharp increase of NDVI, leads to a false detection of early green-up (Wang et al. 2018), we used a method adapted from Busetto et al. (2010) and Filippa et al. (2019). We first identified potentially snow-affected data points as those NDVI polygon-level averages having at least 5% of snow-covered pixels (based on the NDSI snow score). Then we computed a polygon-specific minimum baseline NDVI value as suggested by Jönsson et al. (2018), i.e. the 5th percentile of average NDVI distribution for the polygon, excluding potentially cloud- and snow-affected observations. Finally, for such potentially snow-affected observations, we kept the maximum between the values of observed NDVI and the baseline minimum.

For Sentinel-1 CR we kept all the available observations, i.e. ascending/descending and overlapping orbits. By combining ascending and descending passes, the revisit time over Europe can be between two and four days for non-overlapping orbits, and less when orbits overlap. The effect of different incidence angles on backscatter coefficients is attenuated by the use of the VH/VV ratio (Schlund and Erasmi 2020). Where orbit overlap occurs, mostly towards more Northern latitudes, and multiple observations are available on a single day, we retained their average.

**Fig. 1:**
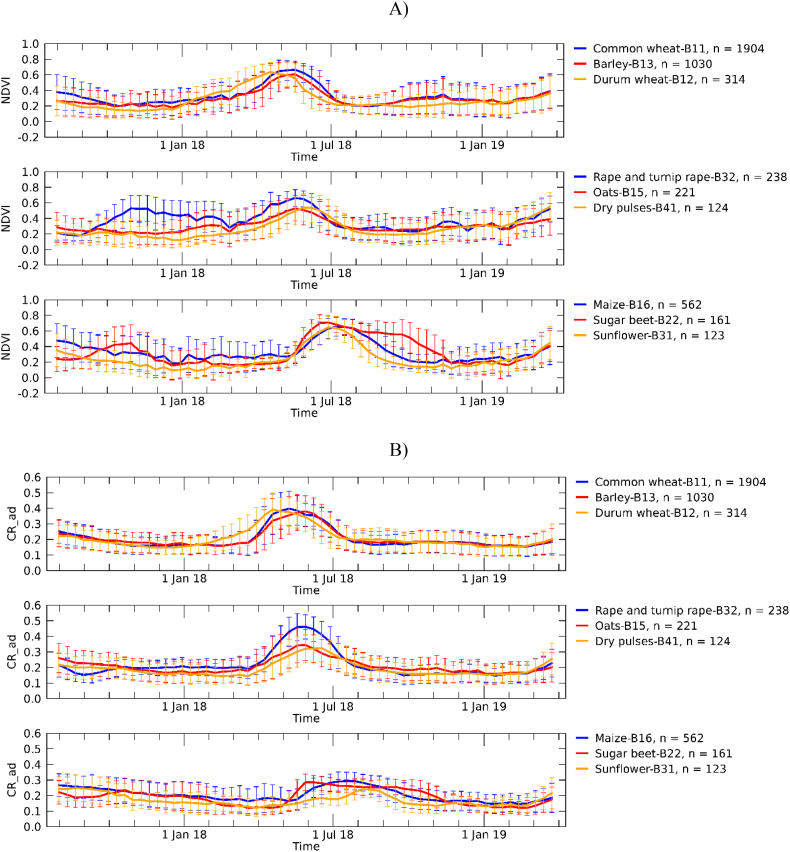
Average per-crop temporal profiles of NDVI from S2 (A) and CR from S1 (B). The label ‘CR_ad’ is used to indicate that both ascending and descending orbits of S1 were used. Each crop profile is obtained by averaging the profiles of all LUCAS-Copernicus polygons of that specific crop. Error bars refer ±1 SD. The number of polygons per crop is reported in the legend.

**Fig. 2:**
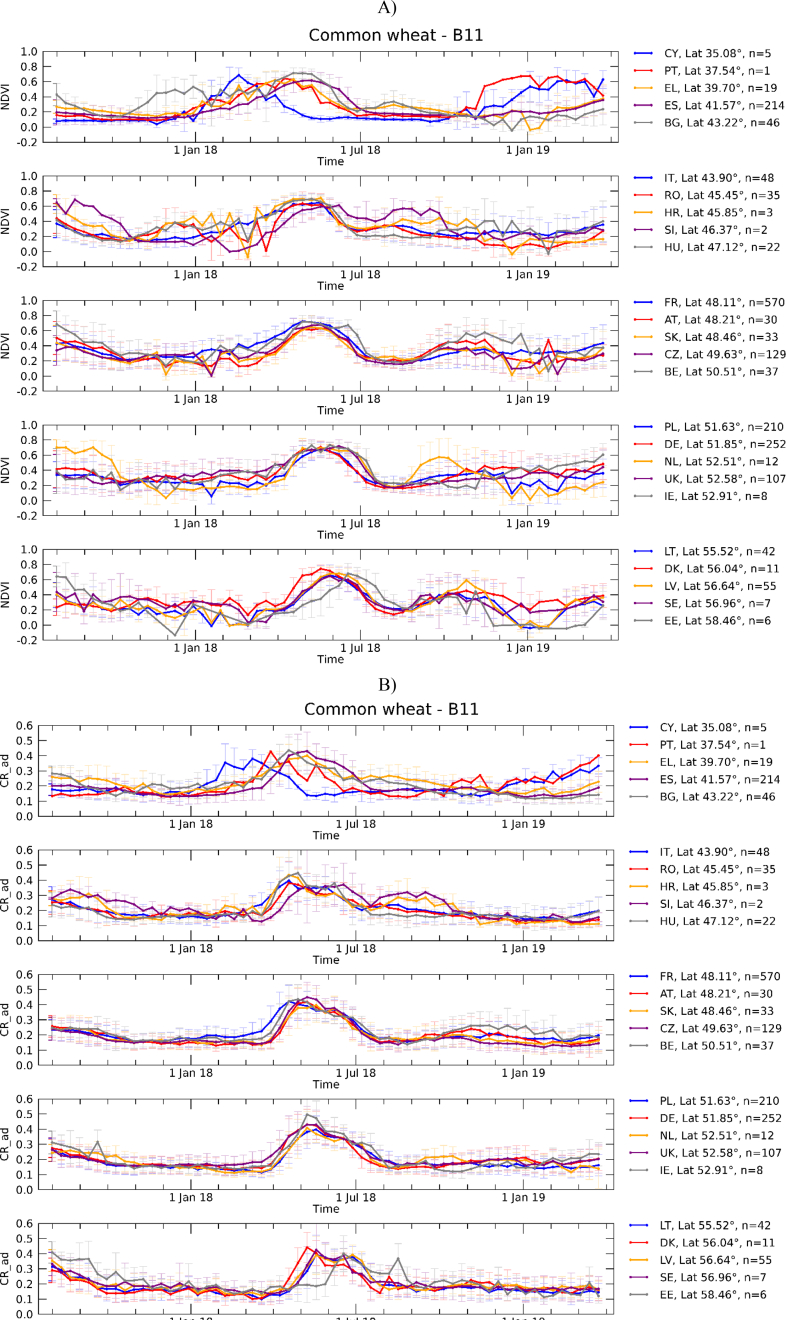
Country-level average wheat temporal profiles of NDVI (A) from S2 and CR (B) from S1. A single country profile is obtained averaging all polygon-level mean profiles in the country. Error bars refer ±1 SD. Countries are ordered by increasing latitude (i.e. average latitude of all the polygons within the country), reported in the legend with the number of polygons per country. The list of country codes can be found in Supplementary Table S1.

As an example of the gathered dataset, EU-level average profiles for all crops are shown in Fig. 1 and country-level average temporal profiles for wheat in Fig. 2 (other crops in Supplementary Fig. S1). For the purpose of presenting the multi-polygon profiles, single profiles with irregularly gridded observations were temporally composited at regular 10-day intervals using the mean. With the exception of rape and turnip rape, where only NDVI appears to respond to the early plant development in autumn, NDVI and CR temporal profiles of winter crops show larger similarities compared to that of summer crops. Large standard deviations of both EU- and country-profiles highlight large variability in crop development and phenology. In Fig. 2, a slight shift of the peak towards later dates with increasing latitude is more evident in NDVI but visible in CR as well.

### 3.2 LSP extraction

We used the same method to extract the phenological timing from S1 and S2. Therefore, in the following we will refer to a generic vegetation index (VI) to indicate both S1 CR and S2 NDVI. Minor VI-specific adaptations will be detailed in the text.

#### 3.2.1 Identification of the crop-specific time domain

**Fig. 3:**
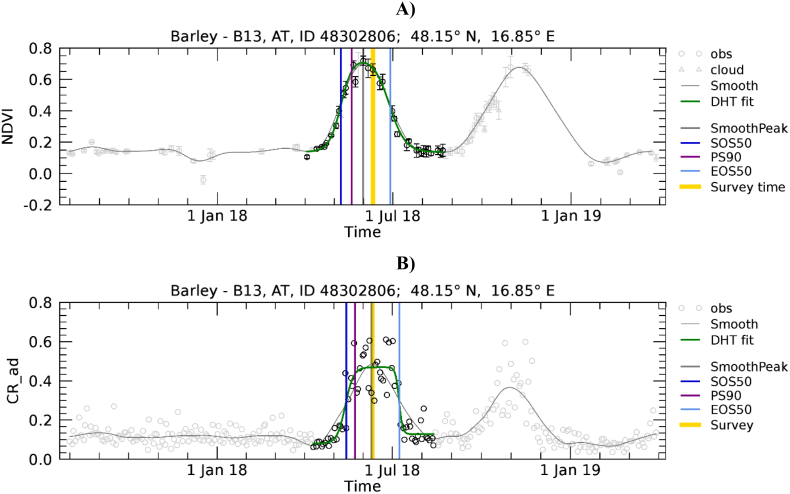
Temporal profiles of NDVI (A) and CR (B) extracted for a barley polygon in Austria, the area of the polygon in number of pixels (n) is 49.2. CR is labelled as CR_ad to indicate that both ascending and descending orbits are used. The LUCAS-Copernicus point identification number (ID) and coordinates of the polygon centroid are in the title. VI data points are polygon averages, ± standard deviation (SD) in the case of NDVI. Data points falling in the identified crop-specific time domain are in black, others in grey. NDVI data points are represented by circles when the observation is considered cloud-free, triangles when it is cloud affected (and thus not considered in further processing, see Section 3.1), and squares when the observation was contaminated by snow and replaced by the background value (Section 3.1). The grey continuous line is the temporally smoothed curve of the data points (Section 3.2.1). The green continuous line is the fitted parametric function (Section 3.2.2). Coloured vertical bars mark the timings of: maximum smoothed value (grey, SmoothPeak), peak season (purple, PS90), SOS extracted using the 50% thresholds (dark blue, SOS50), EOS extracted using the 50% thresholds (light blue, EOS50), LUCAS-Copernicus survey (orange, Survey time). (For interpretation of the references to colour in this figure legend, the reader is referred to the web version of this article.)

We used a model-fit approach to extract phenology. With this approach, a parametric function is fitted to the VI time-series over a defined temporal domain (e.g. Beck et al. 2006; Jönsson and Eklundh 2002; Zhang et al. 2003). Because we expect variability in the timing of crop development over the wide EU territory due to different climatic conditions and management practices, the temporal domain cannot be static and determined a priori (i.e. the same for all polygons of a specific crop). When targeting natural vegetation and using multi-annual VI time-series, the assumption of periodicity and no major inter-annual change of the shape of VI can be made per-pixel to extract the number of growing seasons per year and the seasons' temporal breakpoints (Meroni et al. 2014). These assumptions are not valid for a field-level and crop-specific analysis because different crop types with different phenologies are usually grown in rotation on the same field in successive years. Therefore, temporal profiles from previous years may not be representative of the current crop being grown and consequently we need to rely on the observations of the year of interest only. However, a single annual VI temporal profile often contains information that is not related to the growth cycle of the crop of interest. In fact, multiple peaks may be present, before or after the one of the targeted crop. These additional cycles are related to the natural vegetation growth (weeds) or management practices (e.g. cover or catch crops). An example is shown in Fig. 3 where the barley cycle occurring between May and July is followed by an additional cycle of an unknown vegetation cover with similar magnitude. In addition, some winter crops (e.g. wheat and barley) may present a distinct winter increase of NDVI, followed by winter dormancy (NDVI stagnation) during the first months of the year, followed again by a steep increase afterwards (see Fig. 11C).

Crop cycles are identified as periods of increase and then decrease of the VI happening in the expected, and crop-group specific time period. Based on expert knowledge of the analysts producing the JRC-MARS Crop Monitoring in Europe (van der Velde et al. 2019) and available crop calendars (JRC-MARS, 2020), we defined the time periods during which we expect that the maximum leaf area index of the crop (and peak VI value) should occur: i.e. 1 March – 31 July for winter crops (see Table 2), and 1 June – 31 August for summer crops.

To automatically identify the temporal domain corresponding to the main crop cycle on the VI profile for every single polygon, we proceed as follows (see Supplementary Fig. S2 for a graphical example):

1. We generate a daily time series of the VI using Savitzky-Golay smoothing (Savitzky and Golay 1964). Standard smoothing is applied to CR time series whereas we use the upper-envelope adaption for NDVI adapted from Chen et al. (2004) as described in Meroni et al. (2014).
2. We identify local extrema (maxima and minima) over the smoothed curve.
3. If two identified maxima occur less than 15 days apart, we eliminate the smaller of the two maxima, as well as the minimum in between those maxima.
4. To avoid the selection of a min-max-min segment that is composed of a relevant maximum bounded by irrelevant minima at its sides (e.g. due to noise as min*2* in Fig. S2), we proceed as follows. For each of the maxima, we identify the two neighbouring minima, discarding the shallow ones that are likely unrelated to phenological cycles. We assume that a minimum is phenologically relevant if its removal would cause a significant change in the area below the curve connecting the neighbouring maxima. To achieve this, we compute: A) the VI area below the segment that connects the two maxima and above the overall VI minimum, and B) the area of the triangle connecting the two maxima and the minimum in between. The ratio B/A quantifies the effect of the minimum. The minimum is discarded if this ratio is smaller than 0.15 (this threshold being tuned by visual inspection of a large number of profiles). The smaller of the two neighbouring maxima is discarded as well.
5. Among the remaining maxima, we discard those with very small VI values. To do so, we compute height of the peak (maximum value minus the smallest of the neighbouring minima) and we discard it, as well as the largest of the two surrounding minima, if smaller than 0.1 and 0.075 for NDVI and CR, respectively.
6. The area below each remaining min-max-min segment in the crop-group time period is computed and the period with the largest area selected to automatically focus on the main crop.

For CR, we proceed as above for steps 1 to 5. After that, having recognised that the CR cycle corresponding to the NDVI cycle is not always the one with the largest area under the CR trajectory, we select the min-max-min segment that is closest to the one identified by NDVI. In this way we avoid to introduce differences in the retrieved phenology that are solely due to the different identification of the crop temporal domain. As a final result of this procedure we identify a time domain for NDVI and one for CR that are used to fit the parametric function.

#### 3.2.2 LSP retrieval

Although various methods exist to extract phenological information from remote sensing time series (Zeng et al. 2020), function fitting is best suited for non-dense (S2) or noisy data (S1) (Cai et al. 2017; Jönsson et al. 2018). We retrieved phenology from the raw temporal profiles of NDVI and CR using the fitting function proposed by Meroni et al. (2014). The method is suitable for handling the irregular time sampling of cloud-free S2 data. A double hyperbolic tangent (DHT) function is used to approximate the seasonal development of the crop:

(1) $$\mathrm{VI}\left(t\right)=a_{0}+a_{1}\frac{\tanh\left[\left(t-a_{2}\right)*a_{3}\right]+1}{2}+a_{4}\frac{\tanh\left[\left(t-a_{5}\right)*a_{6}\right]+1}{2}-a_{4}$$

where VI is the vegetation index used (either NDVI or CR), *t* is time (days), *a*_*0*_ is the background minimum VI value, *a*_*1*_ and *a*_*4*_ are the VI amplitudes during green-up and decay phases, *a*_*2*_ and *a*_*5*_ are the timing of inflection points of the two phases, *a*_*3*_ and *a*_*6*_ control the steepness of the two phases. A hyperbolic tangent is functionally equivalent to the logistic function, which is extensively used for retrieving phenology (e.g., White et al. 2014; Zhang et al. 2003). Different from the six-parameter formulation of double logistic models (e.g., Beck et al. 2006; Jönsson et al. 2018), we added a seventh parameter to account for the fact that minimum VI values may differ before and after the season.

The parameters of the DHT functions are determined using the constrained Levenberg–Marquardt least squares fit (Markwardt 2009) between the modelled VI data at daily time-step and observed VI data over the time interval defined in Section 3.2.1. Constrained optimization is used to avoid that the function takes unrealistic shapes due to missing or noisy data. Since NDVI values may be biased towards lower values because of undetected clouds, the model optimization for this VI is adapted to the upper envelope of the observations using an iterative weighting scheme similar to that proposed by Chen et al. (2004). Initial and constrain values of model parameters are set as described in Table S2 of the Supplementary Material.

Timings of SOS and EOS are defined using thresholds applied to the green-up and decay phases of the fitted function. SOS occurs when the value of the fitted curve exceeds the initial base value plus a fraction of the green-up amplitude. EOS occurs when the value of the fitted curve drops below the final base value plus a fraction of the decay amplitude. For both metrics, we considered a 50% threshold as in various previous studies (Nijland et al. 2016; Vrieling et al. 2018; White et al. 1997).

We then computed an indicator of the timing of the peak season, PS90, occurring when the function reaches 90% of the green-up amplitude. We found PS90 to be a more robust measure as compared to the time when maximum VI is reached, because the fitted DHT function frequently results in a stable plateau of relatively large VI values (Vrieling et al. 2018).

The accuracy in the determination of LSP metrics depends on the quantity of observations and their distribution over time. When insufficient data are available at the beginning or end of the season, the corresponding phenological timing (SOS or EOS) are uncertain. To avoid artefacts, the model fit is not attempted when: i) there are less than eight valid observations in the identified temporal domain, ii) there is very small variability in the observations (95th–5th percentile difference smaller than 0.1 VI units). Finally, results of the model fit are discarded if there is unbalanced observation availability, i.e. insufficient (less than three) available valid data points in the green-up or decay phase.

### 3.3 Comparison of LSP derived from S1 and S2, and with ground observations

S1- and S2-derived LSP timings were compared in terms of Pearson's correlation coefficient; *P*-value of the linear regression with S1 and S2 timings as independent and dependent variables, respectively; mean absolute error (MAE); and bias or mean error (ME) computed as the mean difference between S1 and S2 timings.

Establishing a direct geographical link between ground-observed phenology and satellite-derived phenology was not possible because the LUCAS-Copernicus and DWD observations in Germany do not occur at the same locations. The precise location of observed DWD fields is not available because DWD provides the nominal locations, which are known to be within 5 km from the actual fields that were observed. In addition, the large variability at small spatial scale and the lack of clear spatial pattern in the timing of the various DWD phenological stages (see Supplementary Fig. S3 for an example) prevents any attempt to interpolate DWD observations and then establish a geographical link with the LUCAS-Copernicus polygons. Therefore, we used DWD phenology to gain insight into which phenological stage is on average associated to our LSP metrics (SOS50 as indicators of green-up, PS90 as indicator of peak, EOS50 as indicator of senescence/harvest). For each crop type, we computed the country-level mean date of each LSP metric and of each of the reported BBCH stage. Then, we investigated which observed BBCH stage occurred closest to each of the three LSP metrics.

## 4 Results

Out of 4659 polygons, phenology from NDVI was successfully retrieved for 4543 of them (97.5%). For the remaining 116 cases, retrieval did not succeed in 29 cases because of insufficient valid observations, and in 87 cases because of small NDVI variability or insufficient availability of observations in the green-up or decay phase. Phenology retrieval from CR failed for a smaller number of polygons (i.e. 52, CR success rate = 98.9%), which was in all cases because of insufficient temporal variability in the CR trajectory.

### 4.1 LSP of the main crops in the EU

**Fig. 4:**
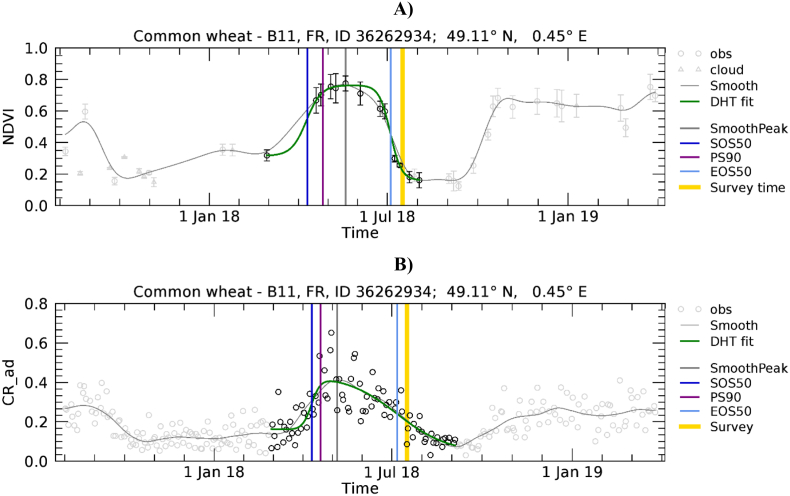
Temporal profiles of NDVI (A) and CR (B) extracted for a common wheat polygon in France, *n* = 36.5 pixels. For a description of graph elements refer to Fig. 3.

**Fig. 5:**
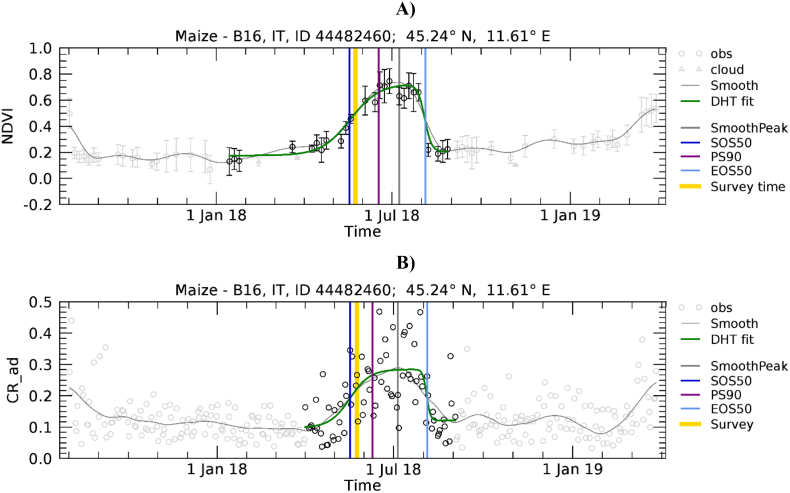
Temporal profiles of NDVI (A) and CR (B) extracted for a maize polygon in Italy, *n* = 17.7 pixels. For a description of graph elements refer to Fig. 3.

Examples of phenology retrieval are shown in Figs. 4 and 5 for a winter and a summer crop, respectively. Obviously, S1 shows a time series of observations denser than that of S2. Presence of clouds during the green-up phase can reduce the number of valid S2 observations and make the shape of the fitted function locally dependent on fewer observations. For example, this is the case of Fig. 4A where only limited observations are available at the beginning of the period (January–March).

**Fig. 6:**
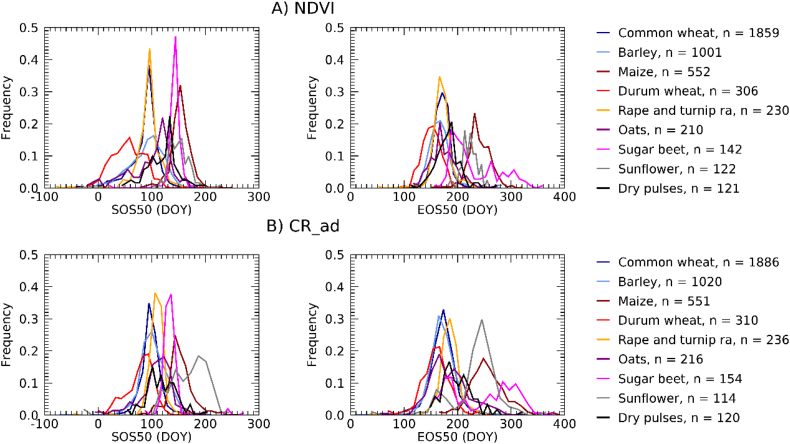
Frequency histograms of SOS50, EOS50 as derived from NDVI (A) and CR (B). Timings are expressed in day of year (DOY) of 2018, negative values indicate dates in 2017. The sample size (n) per crop type refers to the total number of polygons for which phenology was successfully retrieved using NDVI (A) and CR (B). The frequency histogram of PS90 is reported in Supplementary Fig. S4.

Frequency histograms of phenological timing across the EU as derived from NDVI and CR are reported in Fig. 6A and B, respectively. NDVI- and CR-derived phenological timing distributions are in most cases comparable, with notable exceptions like the SOS50 of durum wheat and sunflower, and the EOS50 of sugar beet and sunflower.

**Fig. 7:**
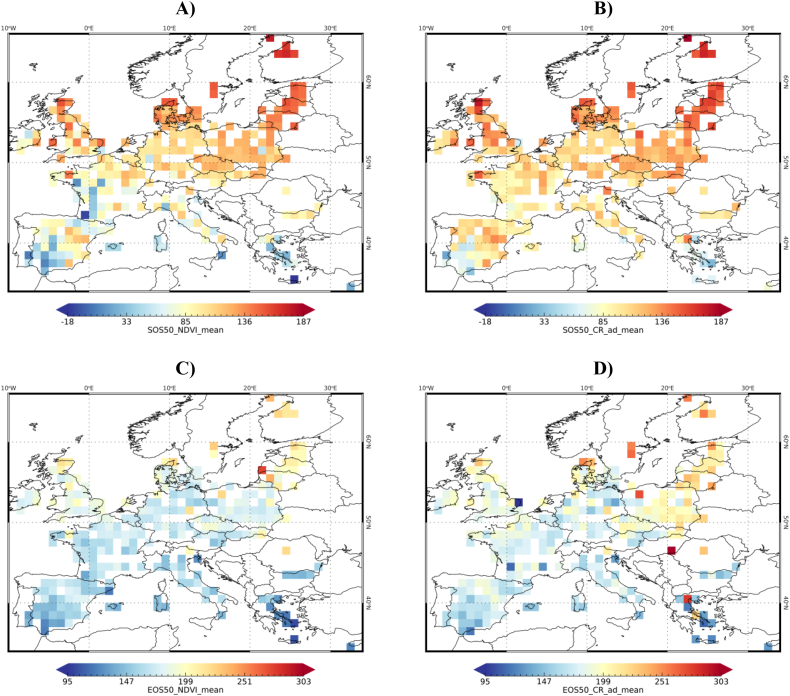
EU-level maps of barley SOS50 (A and B) and EOS50 (C and D) derived from NDVI (A and C) and CR (B and D). Maps were obtained by averaging the phenological timings of the polygons available within the cells of a 1 degree grid. The number of polygons per grid cell is shown in Supplementary Fig. S6.

In order to produce an overview picture of crop phenology in the EU we averaged, per crop type, all the retained LUCAS parcels with successful phenology retrievals over a 1-deg grid. NDVI- and CR-derived barley SOS50 and EOS50 are reported in Fig. 7 as an example (other crop types in Supplementary Fig. S5). LSP extracted from the two VIs describe similar spatial patterns, although with some important differences as for instance SOS50 in western France and some individual cells with late EOS50. A general gradient exists with the Mediterranean countries starting and ending earlier than continental and north-eastern areas. Observed small-scale spatial variations can be due to local climate and management practices. In addition, spatial variability can partly be explained by the fact that the LUCAS class barley includes both winter and spring barley, having different phenology (spring barley being sown much later). The relative abundance of the two varieties within the grid cell average may thus introduce further spatial variability of LSP thus cause the resulting phenology to be earlier (more winter barley polygons) or later (more summer barley polygons).

### 4.2 LSP from Sentinel-1 vs. Sentinel-2

**Fig. 8:**
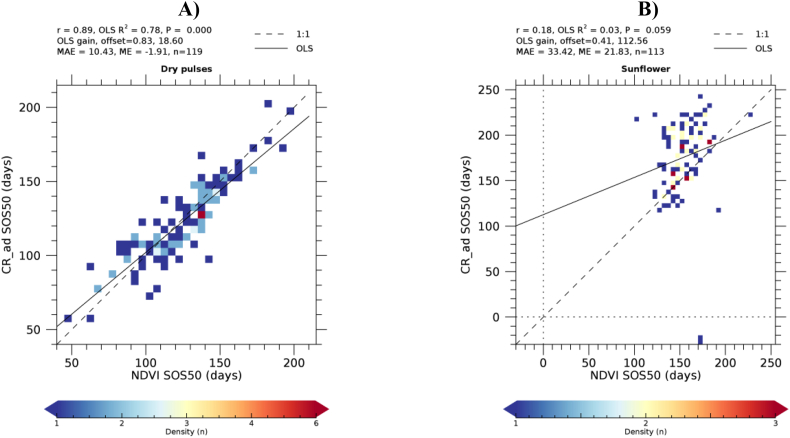
CR- vs. NDVI-retrieved SOS50 density scatterplots for dry pulses (A) and sunflowers (B). LSP timing is expressed as days from 1/1/2018, negative values thus indicating dates in 2017.

For the three LSP metrics considered, Table 3 shows the statistics of the relationship between the CR- and NDVI-derived timings. A significant linear relationship (*P* < 0.01) is found for all LSP metrics and all crop types with the exception of EOS50 of sugar beet and SOS50 of sunflowers. The Pearson's correlation coefficient is in all cases larger for winter crops as compared to summer crops. Correlation decreases for successive phenological events during the crop cycle: it is largest for SOS50 (0.67 on average), followed by PS90 (0.59) and EOS (0.53). The MAE between NDVI- and CR-derived phenological timings is about two weeks for winter crops (between 14.41 and 18.11 days) and larger for summer crops (between 18.91 and 38.26). We note that low correlations do not always coincide with larger MAE and ME. For example, the SOS of summer crops maize and sugar beets have the smallest MAE and ME values of all crops, but a relatively low correlation coefficient. This is because the timing of SOS of these crops shows less variability as compared to winter crops. With only few exceptions the mean error is positive, indicating that CR-derived phenological metrics are generally delayed as compared to NDVI-derived metrics. As an example of agreement between the retrieval from the two indices, Fig. 8 shows the density scatterplot for the crop types having the strongest and weakest SOS50 correlation, i.e. dry pulses (a winter crop) and sunflower (a summer crop).

**Table 3:** Summary statistics of the relationship between CR- and NDVI-derived LSP: SOS50, EOS50 and PS90. *n* is the number of polygons for which a successful LSP retrieval was achieved using both VIs. r is the Pearson's correlation coefficient, P is the significance level of the linear regression between CR- (dependent variable) and NDVI-derived (independent variable) phenology, MAE is the mean absolute error and ME is mean error or bias. MAE and ME are expressed in days. Positive values of ME indicates that CR-retrieved phenological dates occur after those from NDVI.

		SOS50				EOS50				PS90			
Crop	*n*	r	*P*	MAE	ME	r	P	MAE	ME	r	*P*	MAE	ME
Common wheat - B11	1851	0.67	<0.0001	15.3	11.7	0.50	<0.0001	14.7	4.1	0.65	<0.0001	11.2	1.8
Barley - B13	996	0.81	<0.0001	17.3	15.4	0.72	<0.0001	14.6	10.9	0.75	<0.0001	13.1	8.4
Durum wheat - B12	303	0.73	<0.0001	25.2	21.8	0.62	<0.0001	18.1	9.9	0.71	<0.0001	16.0	9.3
Rape and turnip rape - B32	228	0.71	<0.0001	17.1	14.6	0.67	<0.0001	16.8	13.9	0.63	<0.0001	18.2	14.9
Oats - B15	207	0.86	<0.0001	15.9	11.0	0.58	<0.0001	25.1	13.7	0.83	<0.0001	13.1	5.6
Maize - B16	541	0.57	<0.0001	12.0	−0.6	0.52	<0.0001	25.5	18.6	0.42	<0.0001	18.9	4.4
Sugar beet - B22	141	0.63	<0.0001	11.3	−9.4	0.15	**0.0679**	53.2	12.2	0.28	0.0007	17.3	−10.6
Sunflower - B31	113	0.18	**0.0590**	33.4	21.8	0.35	0.0001	36.0	16.7	0.30	0.0011	40.1	28.4
Dry pulses - B41	119	0.89	<0.0001	10.4	−1.9	0.65	<0.0001	19.2	18.0	0.76	<0.0001	14.7	−2.4

Overall average		0.67	0.0066	17.56	9.38	0.53	0.0076	24.82	13.11	0.59	0.0002	18.08	6.64
Winter crop average		0.78	<0.0001	16.88	12.10	0.62	<0.0001	18.11	11.76	0.72	<0.0001	14.41	6.24
Summer crop average		0.46	0.0197	18.91	3.92	0.34	0.0227	38.26	15.82	0.34	0.0006	25.41	7.42

P-values of non-significant regressions (P>0.01) are in bold.

**Fig. 9:**
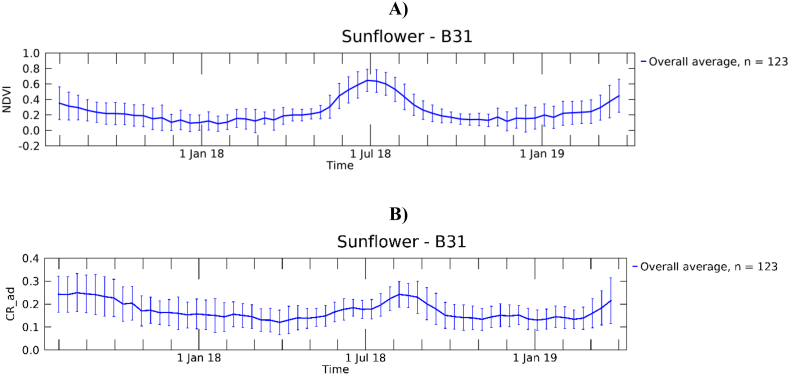
Average temporal profiles of NDVI (A) and CR (B) extracted for all sunflower polygons. Averages extracted as described in Fig. 1.

Disagreement between NDVI- and CR-derived LSP can be attributed to different causes, depending on the crop type. For example, the poor agreement observed for sunflowers (Fig. 8B) is due to the different evolution of the NDVI and CR during the sunflower cycle, especially during the green-up phase from May to July (Fig. 9), resulting in different LSP timings.

**Fig. 10:**
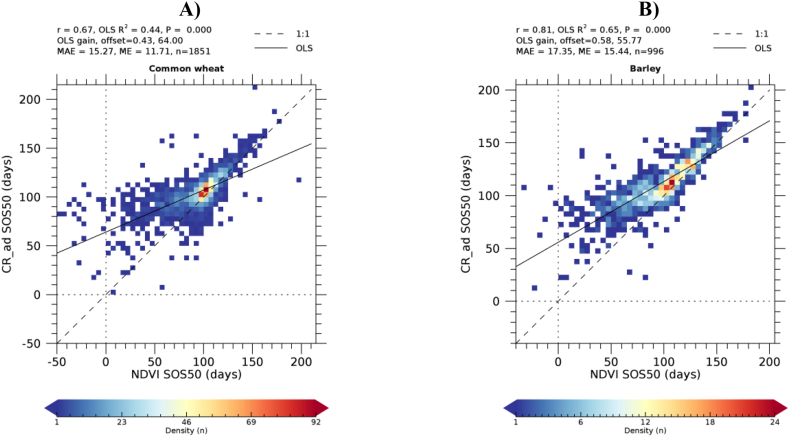
CR- vs. NDVI-retrieved SOS50 density scatterplots for common wheat (A) and barley (B). LSP timing is expressed as days from 1/1/2018, negative values thus indicating dates in 2017.

For winter crops we instead observe a systematic disagreement in LSP under specific circumstances. The correlation coefficient between the NDVI- and CR-derived SOS50 for the two most representative winter crops is reasonably high, i.e. 0.67 for common wheat and 0.81 for barley. The main reason why the correlation is not higher is because of the complex shape of NDVI and CR temporal profiles in the winter and spring season, causing a systematic disagreement in specific cases. Fig. 10 shows the density scatterplot of SOS50 as retrieved from CR (y-axis) and NDVI (x-axis) for all the samples of common wheat (Fig. 10A) and barley (Fig. 10B). While SOS50 has very similar retrievals from both sources for values above approximately 90 days (beginning of April), below this value there is a larger spread, with NDVI-based retrievals consistently being earlier than CR-based retrievals. This is due to two interacting factors: *i*) NDVI is more sensitive than CR to the initial green-up occurring in winter, and *ii*) the presence of a more or less pronounced VI reduction between the winter and spring green-up phases.

**Fig. 11:**
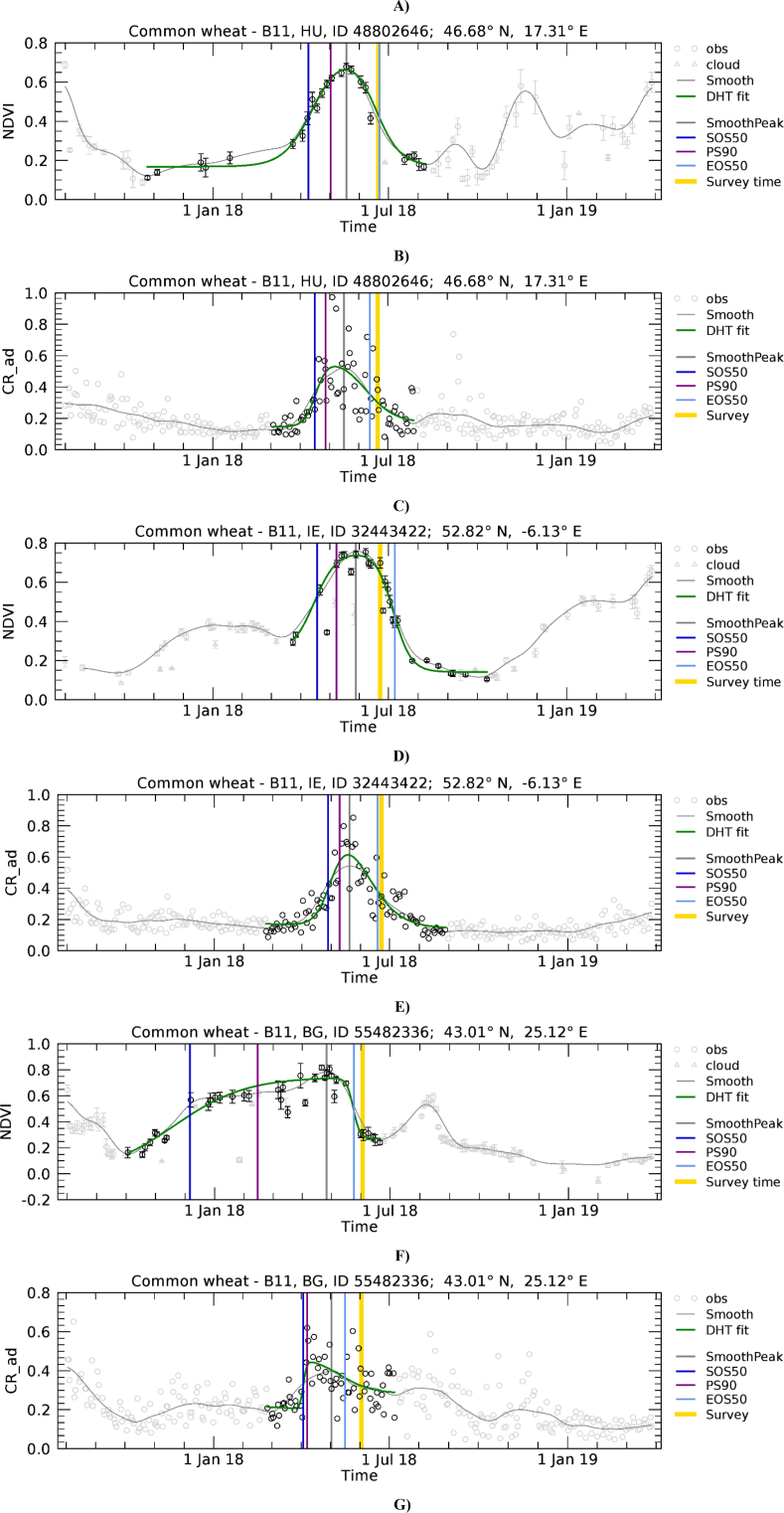

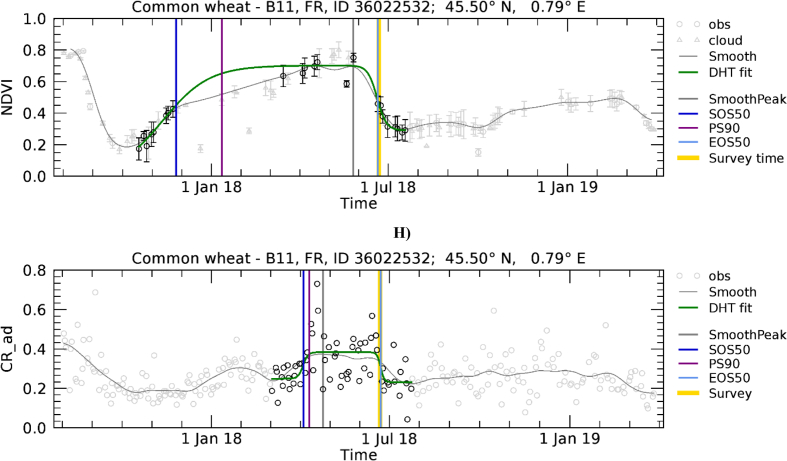
Temporal profiles of NDVI (A, C, E, and G) and CR (B, D, F and H) extracted for wheat polygons in Hungary (A, B; *n* = 13.6), Ireland (C, D; *n* = 46), Bulgaria (E, F; *n* = 22.1), and France (G, H; *n* = 21.6). For a description of graph elements refer to Fig. 3.

Fig. 11 presents the range of wheat development trajectories for Europe to illustrate the variability of winter crop development that we observed in NDVI and CR temporal profiles. The relative importance of the winter and spring green-up phases, as observed on NDVI profiles, varies substantially. In Fig. 11A, only the spring green-up is present. Fig. 11C shows a case where green-up is less important in winter than during spring, while Fig. 11E shows a case where the two peaks are of comparable magnitude. Finally, Fig. 11G shows an example of a profile without S2 valid observations between December and March due to persistent cloud cover. Contrary to NDVI, CR is mostly sensitive to the spring growth. In fact, as observed by Veloso et al. (2017), the CR of cereals remains relatively stable during winter when plants are short and starts increasing significantly at the tillering stage, around the beginning of spring.

**Fig. 12:**
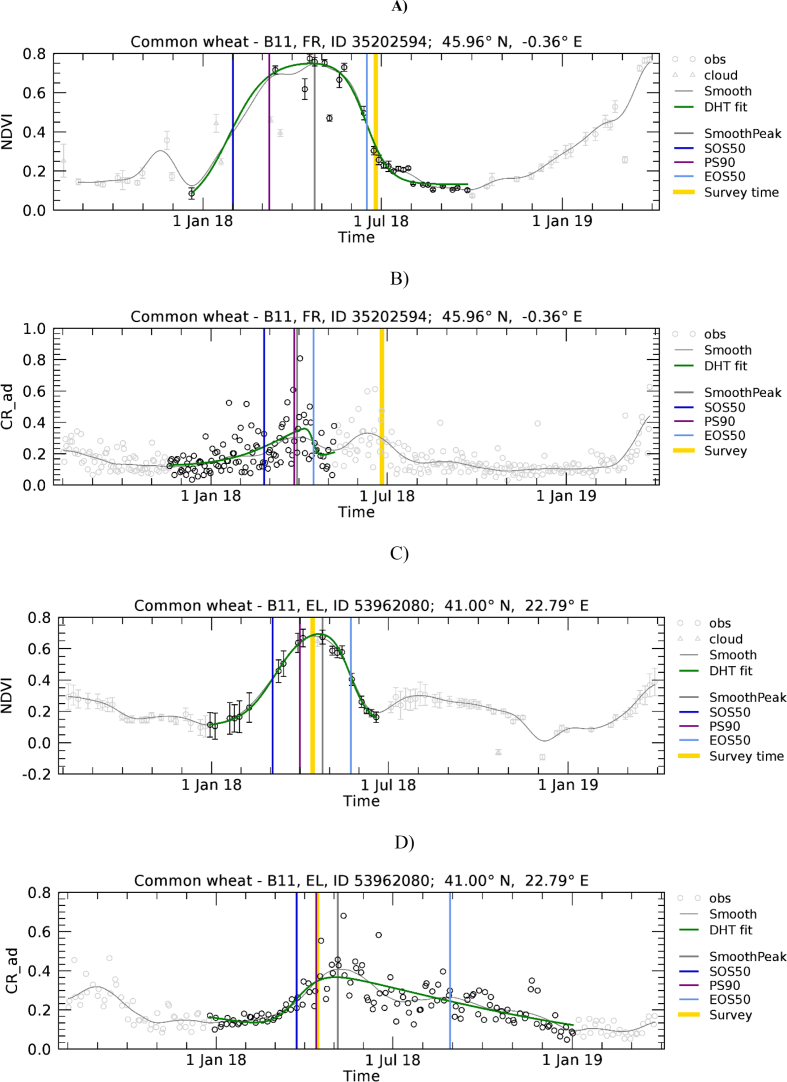
Temporal profiles of NDVI (A and C) and CR (B and D) extracted for wheat polygons in France (A, B; *n* = 18.1), Greece (C, D; *n* = 49.2). For a description of graph elements refer to Fig. 3.

The complex shape of field-level temporal profiles is also a cause for EOS50 mismatch for various cases as those reported as examples in Fig. 12. The phenological cycle of common wheat is correctly identified on the NDVI temporal profile (Fig. 12A and C) that shows a period of sustained increase followed by a sustained period of decrease. On the contrary, the temporal profiles of CR show more complex temporal patterns that result in erroneous identification of the wheat phenological cycles.

### 4.3 Comparison of LSP from Sentinel-1 and -2 with DWD phenology

**Fig. 13:**
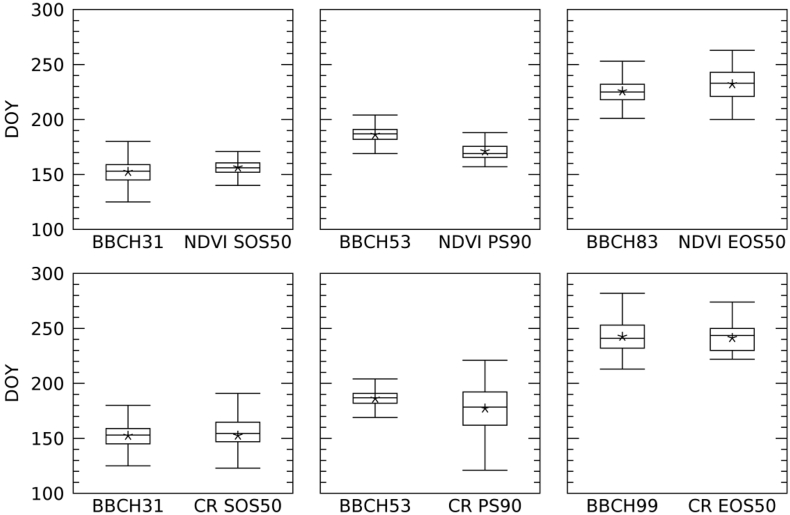
Box-and-whisker plot of the timing of maize LSP metric as detected by S2 NDVI (top row) and S1 CR (bottom row) and corresponding BBCH stage observed by DWD network. The box represents the sample lower quartile, the median and the upper quartile. Whiskers are the sample minimum and maximum, while the star is the sample mean. Sample size as follows: 571 for BBCH31, 592 for BBCH53, 435 for BBCH83, 623 for BBCH99, 65 for NDVI-derived LSP, and 68 for CR-derived LSP.

To assess if our retrievals were consistent with existing field records, we used country-averaged in-situ observations of crop phenology for Germany to gain insights on which phenological stage is linked to our LSP metrics. As an example of the country-level distributions of the LSP metrics and BBCH stages observed by the DWD network, Fig. 13 shows those of maize. Box plots show the distribution of the timings of the BBCH stages closest to SOS50, PS90 and EOS50 derived from NDVI and CR. For SOS50 and PS90, the same BBCH stages are selected independently by the VIs used: 31 (early stem elongation) and 53 (early inflorescence, tip of tassel visible), respectively. The two LSP metrics thus correctly indicate an initial stage of the growing season and a later stage, inflorescence, which occurs roughly half-way through the season. Both NDVI- and CR-derived EOS50 correspond to late season BBCH stage, 99 (harvest) for CR, and 83 (ripening) for NDVI.

Table 4 shows the association between LSP and BBCH stages for all the LUCAS-Copernicus crops that were available in the DWD database. In most cases and for both NDVI- and CR-derived phenology, SOS50 is correctly associated with early BBCH stages (principal stages 1 and 3, leaf development and stem elongation) with a time difference between BBCH and the LSP metric ranging from less than a day to 16 days.

**Table 4:** Identification of the BBCH stage closest to LSP metrics (SOS50, PS90, EOS50) derived from NDVI and CR. Δ is the difference in days between the average timing of the LSP metric and BBCH stage (positive values indicating LSP anticipating the timing of BBCH stage). For a description of BBCH stages and the number of observations see Table 1.

	NDVI						CR					
	SOS50		PS90		EOS50		SOS50		PS90		EOS50	
Crop	BBCH	Δ	BBCH	Δ	BBCH	Δ	BBCH	Δ	BBCH	Δ	BBCH	Δ
Wheat	31	10.0	31	5.5	87	−7.2	31	−4.7	31	6.2	87	−5.7
Barley (winter)	31	−0.1	51	−6.1	87	8.3	31	5.2	51	−6.7	99	−5.4
Barley (spring)	10	−0.3	31	−10.0	87	−8.2	10	5.0	31	−10.6	87	8.2
Rapeseed	51	1.4	61	−1.2	89	−7.5	61	4.7	61	21.4	99	−3.3
Maize	31	4.1	53	−15.1	83	6.5	31	0.1	53	−9.0	99	−1.2
Oats	31	−6.5	31	4.3	87	1.4	31	−1.7	31	6.7	87	−5.7
Sugar beet	35	−6.5	35	6.9	99	−56.3	35	−15.6	35	−4.3	99	−34.4

**Fig. 14:**
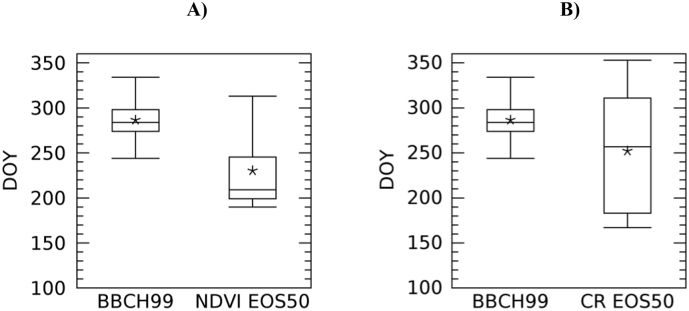
Box-and-whisker plots of the timing of sugar beet EOS50 as detected by S-2 NDVI (*n* = 25, A) and S1 CR (*n* = 27, B) and corresponding BBCH stage observed by DWD network (*n* = 260, in both A and B). The box represents the sample lower quartile, the median and the upper quartile. Whiskers are the sample minimum and maximum, while the star is the sample mean.

EOS50 is also correctly associated with late stages of the crop cycle for both NDVI- and CR-derived LSP. Late secondary stages of the principal stage 8 (ripening) and stage 99 (harvest) are the closest stages to EOS50 timing. The mean difference between the timing of the BBCH stage and that of the LSP metric is smaller than 9 days for all crops except for sugar beet for which the time of EOS50 anticipates the harvest time (BBCH = 99) by about one month for NDVI and two months for CR. For this crop, box plots show that the dispersion of the distribution of the timing of BBCH stage (99, harvest) is large and it is even larger for EOS50 (compare variability of sugar beet in Fig. 14 with variability of maize in Fig. 13), indicating large variability of observed harvesting time and in satellite derived timing of end of season.

**Fig. 15:**
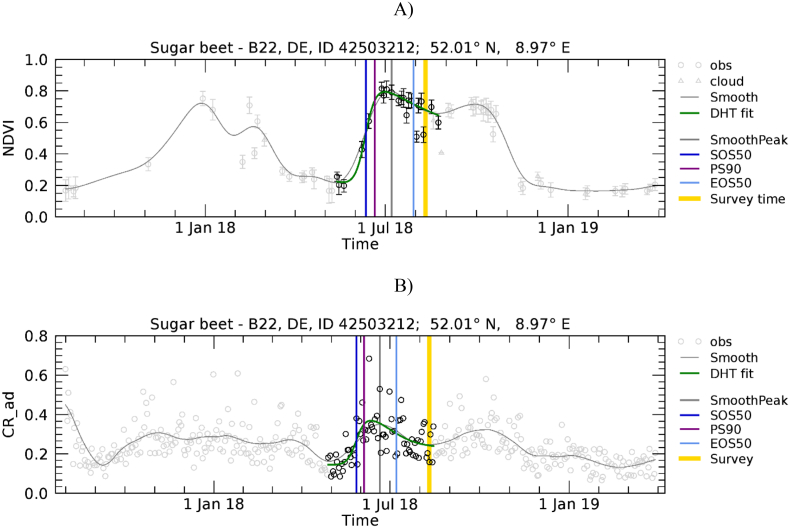
Temporal profiles of NDVI (A) and CR (B) extracted for sugar beet polygon in Germany (*n* = 30.6). For a description of graph elements refer to Fig. 3.

Temporal profiles for sugar beet in Germany reveal that a number of them show a clear VI (both NDVI and CR) reduction in August (see Fig. 15 for an example), obviously unrelated to harvest and instead due to a temporary VI reduction due to the severe summer drought that affected the region.

## 5 Discussion

### 5.1 Agreement between NDVI and CR temporal profiles

The polygon-level average CR profiles (Fig. 4B and Fig. 5B) show less temporal consistency (i.e. smoothness) as compared to NDVI. Although this could potentially be explained by the use of different incidence angles (originating from the use of ascending and descending orbits), we did not detect any improvement in the smoothness of the profile by considering only ascending or descending orbits (see for example the decomposition of the profile of Fig. 5B into single-orbit profiles reported in Supplementary Fig. S7). This confirms that the use of VH/VV ratio is effective in normalizing observations gathered from multiple view angles as suggested by Schlund and Erasmi (2020). Nonetheless, fully quantifying the impact on LSP agreement of separating S1 orbits and considering local incidence angles could be an interesting avenue for further research. A potentially second source of noise in S1 polygon-level averages may be related to the coarser spatial resolution of S1, resulting in a stronger edge effect compared to S2 (i.e. larger sampling of neighbouring and potentially different land cover types).

Despite this noise, a distinct seasonality is observed for CR. This finding is in agreement with the observed crop temporal profiles of Mercier et al. (2020) (wheat and rapeseed), Nasrallah et al. (2019) (wheat) and with Frison et al. (2018) and Proisy et al. (2000) who studied the CR temporal behaviour over mixed temperate forests. In addition, CR temporal evolution coincides with that of NDVI derived from S2, depicting a VI increase in the early and late spring for winter and summer crops, and a sharp decrease in summer. Although the degree of agreement between CR and NDVI varies in our dataset (see Section 4.2), Fig. 4 and Fig. 5 exemplify the potential of CR temporal profiles derived from S1 for phenology retrieval.

### 5.2 Agreement between NDVI- and CR-derived LSP

Our results show potential for the use of all-weather S1 observations to retrieve LSP, as illustrated by the correlation between CR S1- and NDVI S2-derived LSP (Table 3). This observation agrees with Mercier et al. (2020) who found that CR and NDVI, among various features extracted from S1 and S2 data, importantly contributed to obtaining accurate classifications of phenological stages of selected fields of wheat and rapeseed.

We note that the agreement between the two LSP datasets is crop-type dependent and in general larger for winter crops compared to summer crops. Therefore, not for all crops CR-derived phenology may simply be used as a substitute for NDVI-derived phenology.

Differences in NDVI and CR phenological timings are partially related to intrinsic differences in the measured signal. While NDVI is a measure of greenness and thus a combination of canopy cover, biomass amount and leaf chlorophyll concentration, CR is sensitive to standing biomass and the 3D structure of the canopy-soil complex. Backscattering of the canopy is primarily determined by the spatial distribution of leave angles and the size of the leaves, relative to the incidence microwave wavelength (56 mm for C-band Sentinel-1), and their water content (Ulaby et al. 1990). For the soil component, soil moisture and surface roughness regulate backscattering. Before crop canopies close, multiple scattering effects between the soil and the canopy structure contribute significantly to the total backscatter (Bouman and van Kasteren, 1990). This can lead to directional effects, especially for row cropping systems or in canopies that never fully close (e.g. sunflowers). Direct surface scattering effects are typically strongest in the co-polarized channel (VV), whereas multiple volume scattering effects of canopy structure are more important in the cross-polarized channel (VH) (McNairn and Brisco 2004).

Changes in leaf area and leaf chlorophyll content are detected by NDVI and are likely to influence CR as well because these changes are typically associated with changes in leaf water content, changes in the soil cover and structural plant development. However, the timing at which chlorophyll, water content, and structure vary (in response to crop development, weather events or senescence) may not perfectly match, causing differences in the shape or synchronicity of the temporal profile of the two VIs. This can partially explain the observed delay in CR SOS50, as NDVI is sensitive to initial green leaf development, whereas vegetation of limited height hardly affects the SAR backscatter. This was also observed by Veloso et al. (2017) for cereals. In other words, NDVI may respond to the initial increase of green biomass that is not accompanied by synchronous structural development of the crop, occurring later and thus detected later by CR. Similarly, at the end of the crop cycle, NDVI would decrease in response to the drying-up of the crop while CR would still respond to the standing structure of the crop. When NDVI abruptly decreases for those crops that are harvested when still relatively green (e.g. maize), CR may still respond to crop residues standing in the field such as maize stubble (Veloso et al. 2017). Our findings are in agreement with those of Schlund and Erasmi (2020) who reported that CR of selected winter wheat fields did not decrease substantially in all fields because the stubble potentially results in a similar signal compared to the ripe crops, depending on the residual cover.

This (lack of) synchronicity between greenness and structural development is crop-dependent, thus explaining why LSP are at times similar, and other times not when analysing NDVI and CR profiles. For sunflower in particular, the results reveal large differences, indicating either that CR Sentinel-1 data are not suited for retrieving its phenology, or at least that they do not capture the same SOS50 and EOS50 as NDVI. The relatively open 3D structure of this crop is likely the primary explanation of this mismatch. For this crop, CR is not synchronous with photosynthetic development as seen by NDVI (Fig. 9), in agreement with Veloso et al. (2017) who, comparing NDVI and CR in southwest France, found large discrepancies in their temporal profiles and explained it by the large increase of VV backscatter in July caused by flowering.

For winter crops we observed a systematic SOS50 disagreement due the presence of a more or less pronounced VI reduction between the winter and spring green-up phases. This transition between winter dormancy and spring green-up is poorly modelled by LSP extraction methods, typically assuming that the vegetation cycle is composed of a single phase with a consistent green-up. This is also true for our model fit approach that uses one double hyperbolic tangent function to describe the seasonality. However, this function may not always appropriately represent the development of winter crops, which are at times characterised by an initial NDVI green-up when plants emerge from the ground and begin to tiller (e.g. in October – November), before entering into a dormancy period during the darkest and coldest months (December – February). During winter dormancy crop development stops, and crops may experience a reduction of greenness. Depending on latitude and local climate, crops can also be covered by snow during this period. Afterwards, when favourable temperature and photoperiod conditions return, crop growth continues, stems elongate, and thus a second green-up begins (around March). Because the initial green-up generally results in sparse vegetation with limited plant height, this vegetation still has a limited effect on SAR backscatter, resulting in stable low CR levels or a less pronounced increase as compared to NDVI. Fig. 11 shows a range of examples characterised by different shape of VI evolution during the winter to spring transition and its effect on LSP retrieval.

The temporal domain of the mono-modal NDVI profile of Fig. 11A is correctly determined and both the NDVI and CR profiles are nicely fitted by the DHT model. As a result, the various LSP timings roughly coincide. It is noted that here SOS50 refers to the spring green-up for both NDVI and CR, has a value larger than DOY 90, and consequently is found close to the 1: 1 line of Fig. 10A. Fig. 11C and E show NDVI temporal profiles presenting a green-up starting roughly in November, a relatively flat period between January and March and a second green-up in April – May. In both cases CR detects the spring growing cycle only. On the contrary, the current model-fit approach applied to NDVI data yields different results depending on the “depth” of the dormancy period. When the dormancy period results in a clear minimum of NDVI between the two growth times (Fig. 11C), it detects two separate NDVI cycles and focuses on the larger one starting in spring. When the dormancy period is less evident (Fig. 11E), it detects one larger cycle covering both growth times. As a result we observe an agreement between NDVI- and CR-derived SOS50 for Fig. 11C and D, but a mismatch for Fig. 11E,F and G,H. These last two samples would thus be placed in Fig. 10A in the region where CR shows substantially later dates than NDVI SOS50 (data points above the 1:1 line with NDVI SOS50 smaller than DOY 90). This behaviour explains the systematic deviation between the SOS50 observed when the start of the winter growth can be detected by NDVI (Fig. 10A and B) and suggests potential for using both VIs concurrently for a full characterization of the growing cycle, with NDVI and CR supporting the detection of the start of the growth in winter and spring, respectively.

Cases of EOS50 mismatch as those reported in Fig. 12 can also be explained by the difficulty of the current algorithm in interpreting the complex shape of field-level temporal profiles and in focusing on the same time segment when analysing NDVI or CR. For both common wheat polygons presented in Fig. 12, we observe that the time domain identified on the CR profile does not correspond to the one of NDVI. The CR profile for the first polygon presents a minimum in May when the crop is approximately in the heading stage (Fig. 12B) that cannot be appreciated on NDVI (Fig. 12A). CR may thus more strongly respond to this structural change. For the second polygon, CR presents a shallower minimum in June (Fig. 12D) as compared to NDVI (Fig. 12C) that decreases abruptly in response to harvest. As a result, the crop cycle identified by CR is shorter for the first polygon and longer for the second polygon.

While the consistent application of the relatively straightforward asymmetric curve fitting approach could reveal relevant information on LSP of crops across Europe, it may not always be effective in describing the more complex temporal profiles observed, as shown in the examples of Fig. 11 and Fig. 12. Although we acknowledge that in specific cases, we note that other approaches that do not impose a predefined shape, as for instance the one used by Bolton et al. (2020), would similarly face problems in correctly identifying the same principal crop cycles in both NDVI and CR profiles.

### 5.3 Comparison of LSP with DWD phenology

SOS50, PS90 and EOS50 timings derived from both NDVI and CR were correctly associated with early, intermediate and late stages of observed phenological development (Table 4). Deviations of S1-derived SOS50 and EOS50 timings for wheat from BBCH 31 (early stem elongation, SOS50 delayed of 4.7 days) and BBCH 87 (late ripening, EOS50 delayed of 5.7 days) are comparable to those observed by Schlund and Erasmi (2020) for the same phenological stages using S1 CR and a different retrieval methods and to those of Nasrallah et al. (2019) for the germination and harvesting stages using CR and a multiple-Gaussian model-fit approach. Using RADARSAT-2 and TerraSAR-X SAR data with a dynamic filtering framework to estimate BBCH stages of canola, McNairn et al. (2018) obtained a similar error magnitude during vegetative stages but reported higher accuracies for inflorescence emergence and flowering.

An exception is the SOS50 of rapeseed for which the intermediate BBCH stage 51 is selected for NDVI-SOS50 (inflorescence emergence, “green bud” visible) and BBCH 61 (beginning of flowering) for CR-SOS50. Despite the fact that these stages occur later than the BBCH 31 stage that best linked with SOS50 for wheat, the time difference is small: according to the DWD database for rapeseed, on average stage 51 and 61 happen with a delay of 7 and 19 days with respect to stage 31 (early stem elongation). The observed sensitivity of S1 to rapeseed flowering agrees with the results of d'Andrimont et al. (2020c) who used Sentinel-1 backscatter data to detect the flowering period. The use of input features derived from S1 data (combined with S2-derived input features) was also found useful to increase the accuracy of the classification of rapeseed phenological stages by Mercier et al. (2020).

EOS50 derived from NDVI tends to be associated with earlier BBCH stages as compared to EOS50 from CR (Table 4 and the example for maize of Fig. 13). This difference is observed for various crops and may be due to the fact that CR is more sensitive to vegetation structure and hence declines later when the crop gets harvested. On the contrary, NDVI is also sensitive to leaf chlorophyll concentration, which reduces earlier during ripening of the crop, as already discussed in Section 5.2.

Sugar beet is a notable exception to the general agreement between EOS50 and late phenological stages; EOS50 shows large variability and anticipates the harvest time by about one month for CR and two months for NDVI (Fig. 14). An explanation for this variability and the large shift between observed harvest and the LSP EOS50 metric can be related to the peculiar weather conditions during summer 2018. First, among the considered crops, sugar beet was harvested the latest, between September and November in 2018 according to DWD. During 2018, Germany (as well as large parts of northern and western Europe) suffered from a prolonged drought that extended from summer to autumn, likely resulting in an anticipated senescence and earlier EOS50 (with earlier retrievals for NDVI, which is sensitive to greenness decrease, as compared to CR sensitive to structure). However, despite anticipated senescence, observed harvest time occurred later because farmers have delayed the start of the harvest campaign in the driest zones (JRC-MARS, 2018) waiting for precipitation to increase soil moisture, which facilitates the harvest of the roots. These two factors may explain the large difference between the reported harvest dates and satellite EOS for sugar beet. Second, in a number of cases we observed that the sugar beet temporal profile shows a clear early VI (both NDVI and CR) reduction in August (Fig. 15) due to the severe drought. When this reduction is large, as in the case of Fig. 15, the overall profiles show two peaks and the current algorithm set-up interprets it as two separate seasons with the August reduction detected as EOS of the first season. The presence of such cases makes the dispersion of EOS50 large and contributes further to the observed anticipation of EOS50 with respect to reported date of harvest. We note that both the late harvest and the presence of the August minimum are due to the specific drought conditions in 2018. For operational applications, once this specific artefact has been recognised, we can envisage in future to incorporate expert knowledge on the expected timing/start of the crop harvest to avoid undesired retrievals induced by specific weather conditions, such as those that occurred in 2018. Finally, Table 4 shows that the link of PS90 with BBCH is less interpretable compared to that of SOS50 and EOS50. PS90 derived from both NDVI and CR is associated to BBCH principal stages 3, 5, and 6 (stem elongation, inflorescence emergence, beginning of flowering). For two out of six cases, PS90 is associated with the same the BBCH stage of SOS50 (i.e. wheat and sugar beet). This is due to the fact that PS90 is often close to SOS50, especially when the green-up phase is fast. In all the other cases, PS90 is correctly associated to an intermediate BBCH stage, i.e. between the early ones associated with SOS50 and the late ones associated with EOS50.

## 6 Conclusion and way forward

In this study, crop-specific land surface phenology was retrieved by exploiting Sentinel-1 and -2 time series and extensive field information from the Copernicus component of the EU-wide LUCAS survey. We applied the same retrieval procedure to both multispectral and SAR data to extract the phenology of major European crops growing under heterogeneous climatic and management conditions. We showed that extracted LSP timings are comparable, but that this depends on crop type. This dependence relates mostly to the crop-specific synchronicity between greenness and structural development. For winter crops we observed that SAR is able to detect the start of the spring growth while multispectral data is sensitive to the crop growth occurring before and during winter. The CR (i.e., the cross-polarization ratio VH/VV ratio) computed from S1 data is therefore suitable to estimate the timing of spring green-up of winter crops on a regular basis, inclusive of regions characterised by persistent cloud cover. On average, the correlation between LSP metrics derived from Sentinel-1 and -2 is poorer for summer crops compared to winter crops, although for some summer crop/metric combinations (e.g. SOS50 for maize and sugar beet) this is also due to the limited spatial variability, as mean absolute error and bias could nonetheless be low. Only for a single crop out of the nine crops considered, i.e. sunflower, we conclude that CR is unsuitable for NDVI-like LSP retrieval.

This study clearly shows that Sentinel-1 data have potential for phenology estimation. LSP retrieved using CR reasonably agrees with the typical NDVI-derived LSP, adding evidence that CR enhances the sensitivity of the SAR signal to canopy biomass while minimising other effects, such as background roughness and soil moisture. For crops like dry pulses or barley, NDVI and CR series may be used interchangeably resulting in similar LSP retrievals, while for other crops both signals may provide complementary information. Common wheat is a clear example, where for some samples NDVI-derived SOS50 detects the pre-winter greening, and CR-derived SOS50 the vegetative development in spring. How to best utilize the complementarity of both data streams in refining crop phenological characterization at field and regional levels remains a topic of further research.

The use of ground information from a phenology observation network enabled us to assess if our phenological metrics have a clear link with crop development stages. Although we found this link at an aggregate level for Germany, linking in-situ observations and satellite-derived phenological metrics at field level would be required to better understand the specific advantages of the use of NDVI and CR. In addition, a field-level link would allow crop-specific tuning of the numerical thresholds used to determine SOS and EOS in order to provide the best match between them and the relevant BBCH stages. Given that 2018 was a peculiar year with summer drought affecting significant parts of Europe, a repetition of the same analysis for a more ‘normal’ year may reveal a stronger link with ground information, due to fewer erroneous interpretations of mid-season reductions in greenness.

Finally, we acknowledge that retrieval of crop phenology is not trivial at this fine spatial scale. In fact, despite obtaining crop-specific temporal profiles, we observed that these can be quite complex, showing multiple peaks due to crop dormancy for winter crops and drought-related mid-season reductions in greenness. In such a context, a curve fitting approach that describes a single peak may be suboptimal to represent the complex cases, and further comparisons with applying alternative retrieval approaches to Sentinel-1 and -2 series may be envisaged. In addition, given the continuous secured stream of both data sources, more research is needed to understand how to optimally combine both by tuning the retrieval method to SAR data or by using Sentinel-1 and -2 observations simultaneously. While scope exists for further research, our study has shown that both Sentinel-1 and -2 can play an important role and improved location-specific characterization of crop development stages. Given the importance of such information for crop monitoring, our results are expected to help improve existing crop monitoring systems.

## Credit author statement

MM, RD, A Vrieling, and GL conceived the idea for the study. MM developed the code for Land Surface Phenology (LSP), carried out the LSP retrieval and subsequent data analysis. DF provided contributions in the mathematical development of the algorithm. RD analysed LUCAS-Copernicus data and constructed the crop-specific polygons. RD, GL and A Verhegghen carried out the satellite image processing on GEE and extracted polygon profiles. LS prepared the DWD data and contributed to their analysis. A Vrieling and FR provided inputs for the definition of the analysis and result interpretation. With MM as the core author, all authors contributed to the writing of the manuscript.

## Declaration of Competing Interest

The authors declare that they have no known competing financial interests or personal relationships that could have appeared to influence the work reported in this paper.

## Appendix A Supplementary data

Supplementary Material Image 1
