# Supplementary material for: Comparing land surface phenology of major European crops as derived from SAR and multispectral data of Sentinel-1 and -2
Source: Remote Sens Environ. 2021 Feb;253:112232. doi: 10.1016/j.rse.2020.112232 (PMC7841528; doi:10.1016/j.rse.2020.112232)
Supplement: Supplementary file 1 — Supplementary Material [file mmc1.docx]

**Supplementary Material**

Table S1. Country codes used.

| **Two-letter code** | **Full name** |
| --- | --- |
| AT | Austria |
| BE | Belgium |
| BG | Bulgaria |
| CY | Cyprus |
| CZ | Czechia |
| DE | Germany |
| DK | Denmark |
| EE | Estonia |
| EL | Greece |
| ES | Spain |
| FI | Finland |
| FR | France |
| HR | Croatia |
| HU | Hungary |
| IE | Ireland |
| IT | Italy |
| LT | Lithuania |
| LV | Latvia |
| NL | Netherlands |
| PL | Poland |
| PT | Portugal |
| RO | Romania |
| SE | Sweden |
| SI | Slovenia |
| SK | Slovakia |
| UK | United Kingdom |

| **A)**  **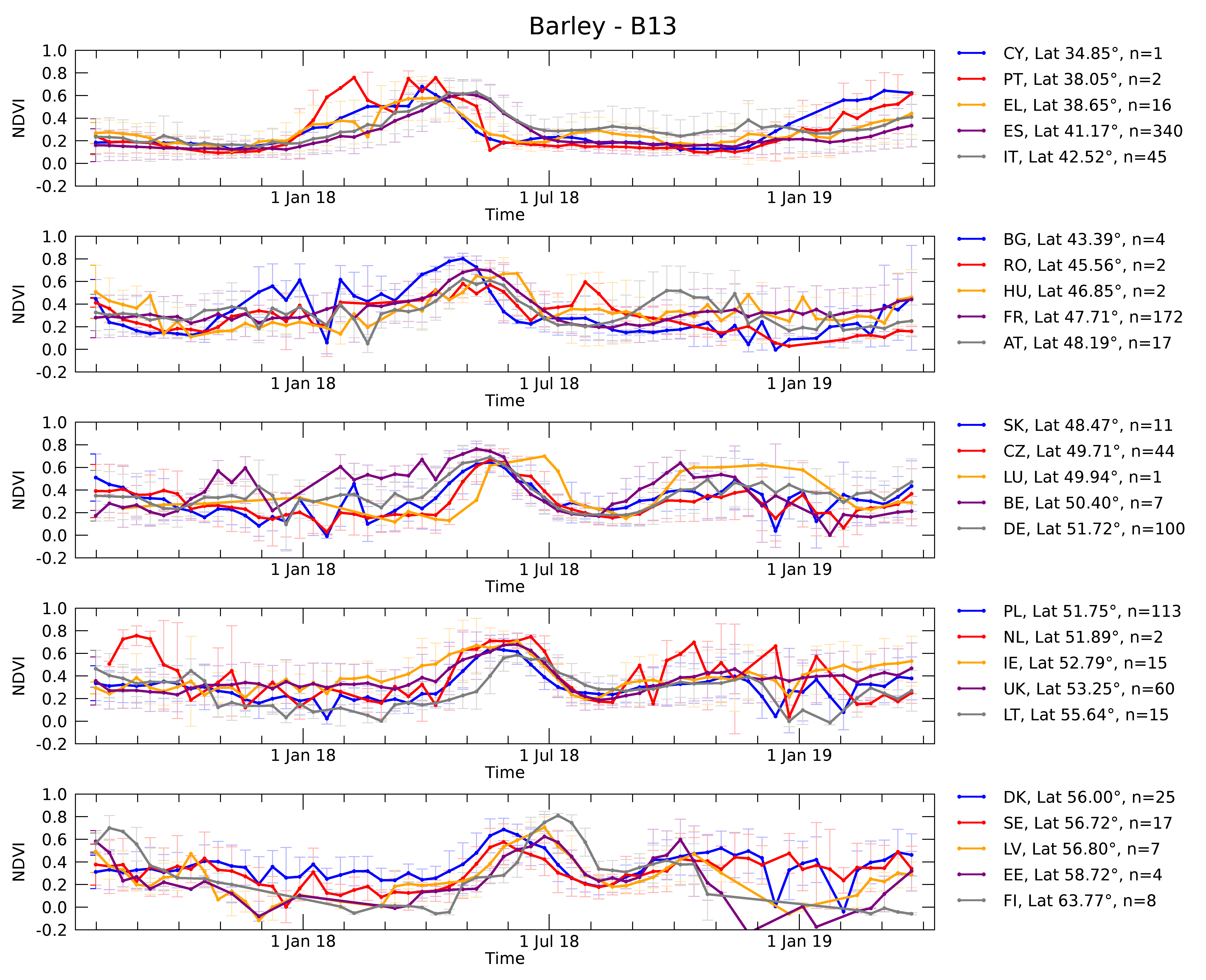** | **B)**  **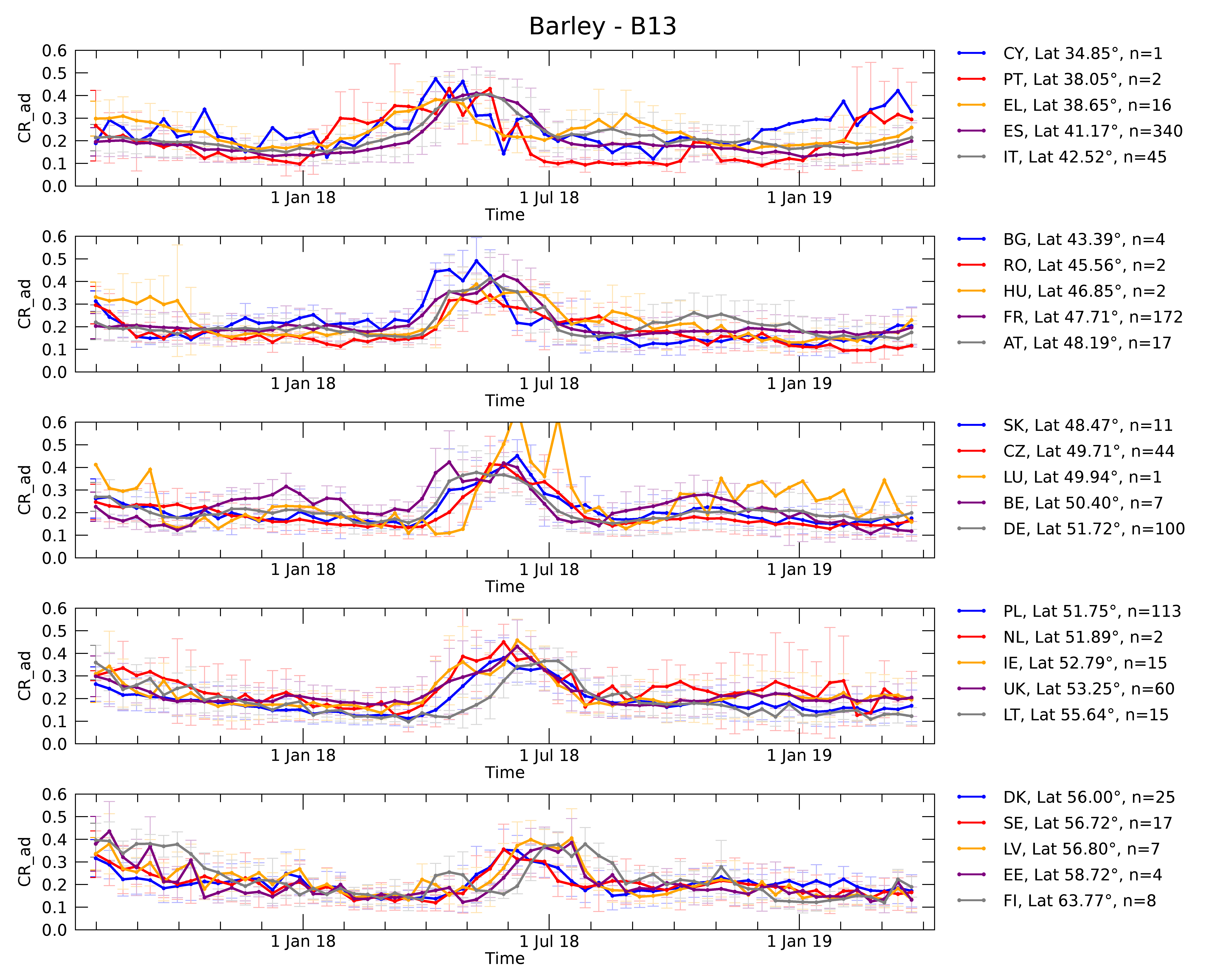** |
| --- | --- |
| **C)**  **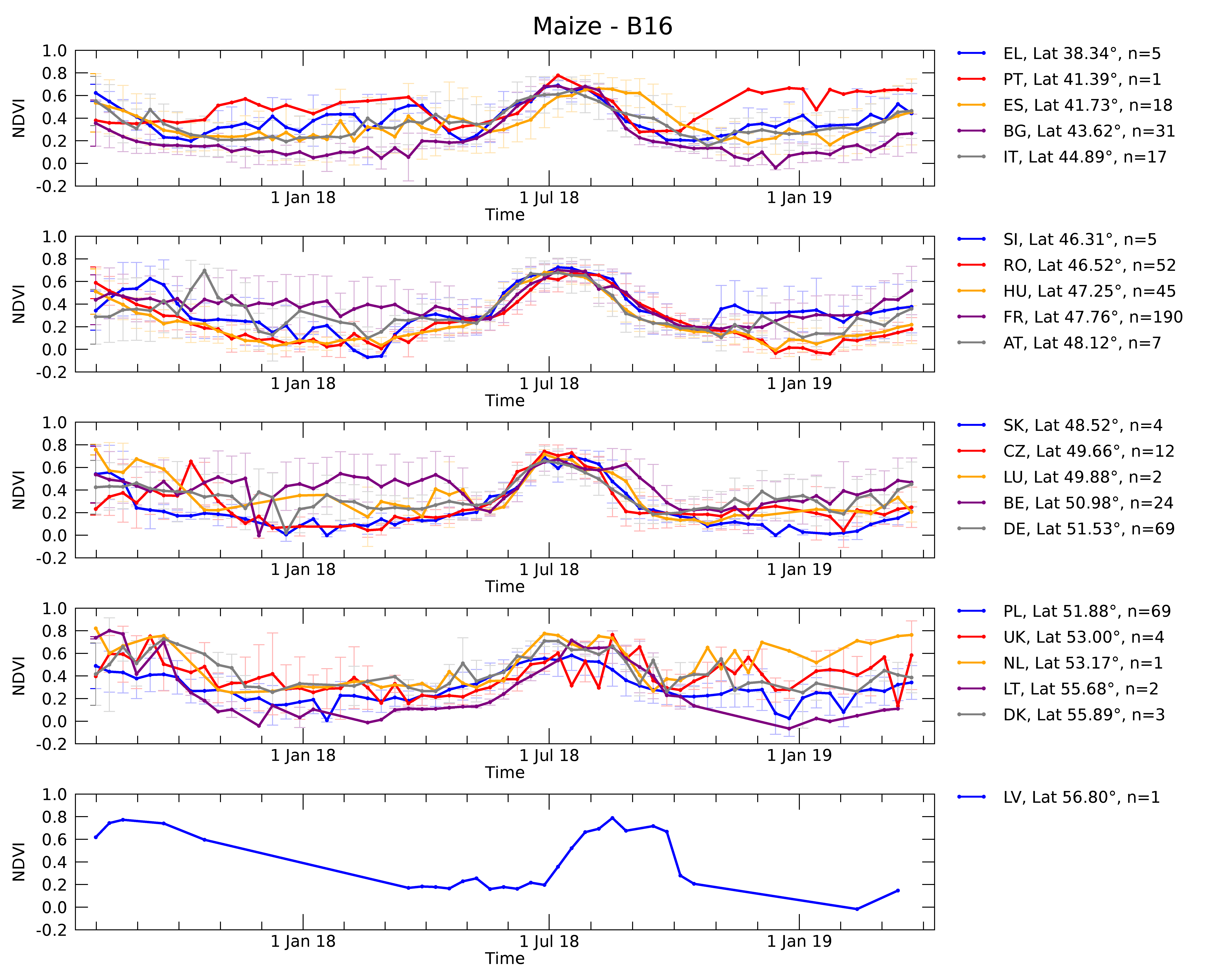** | **D)**  **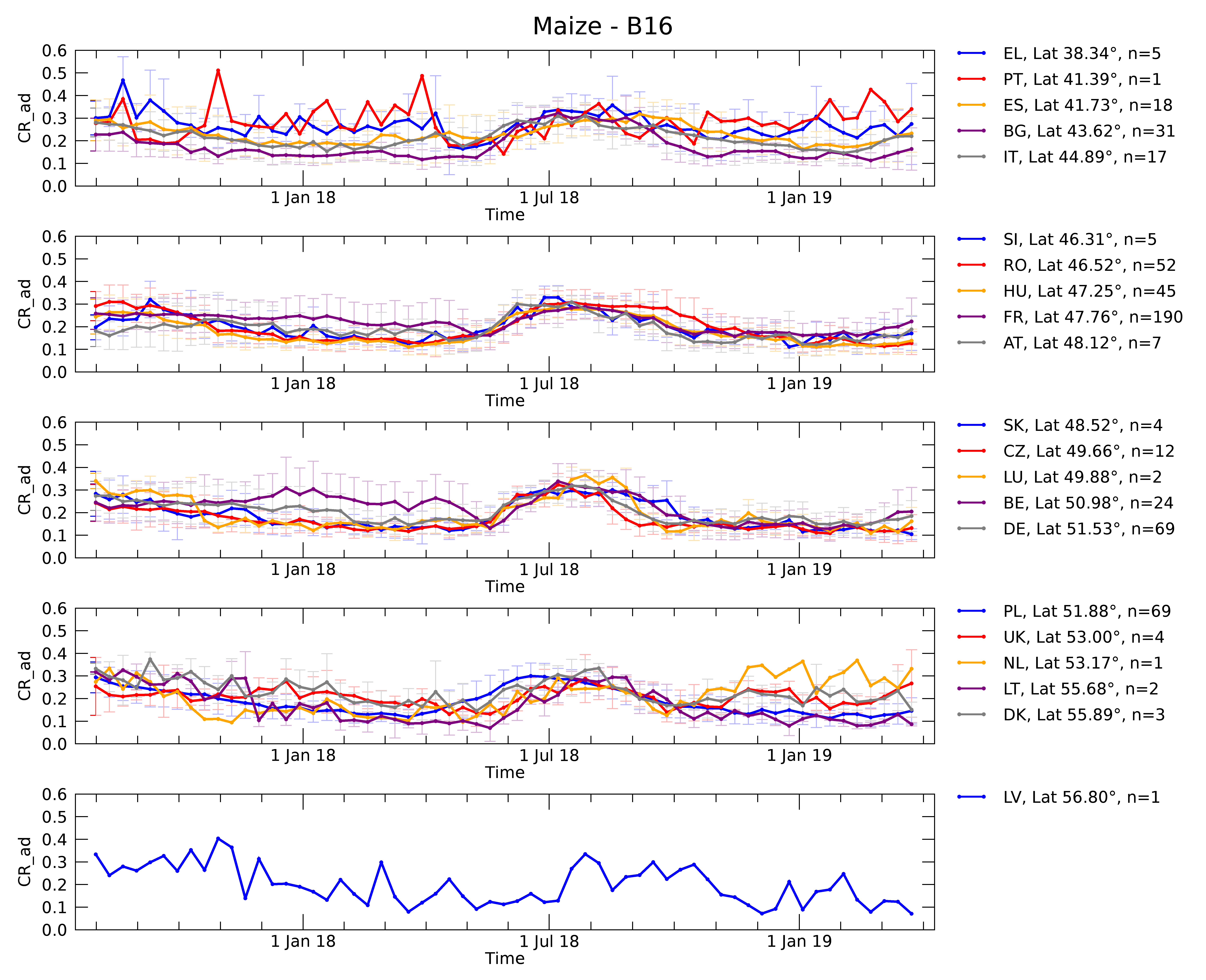** |
| **E)**  **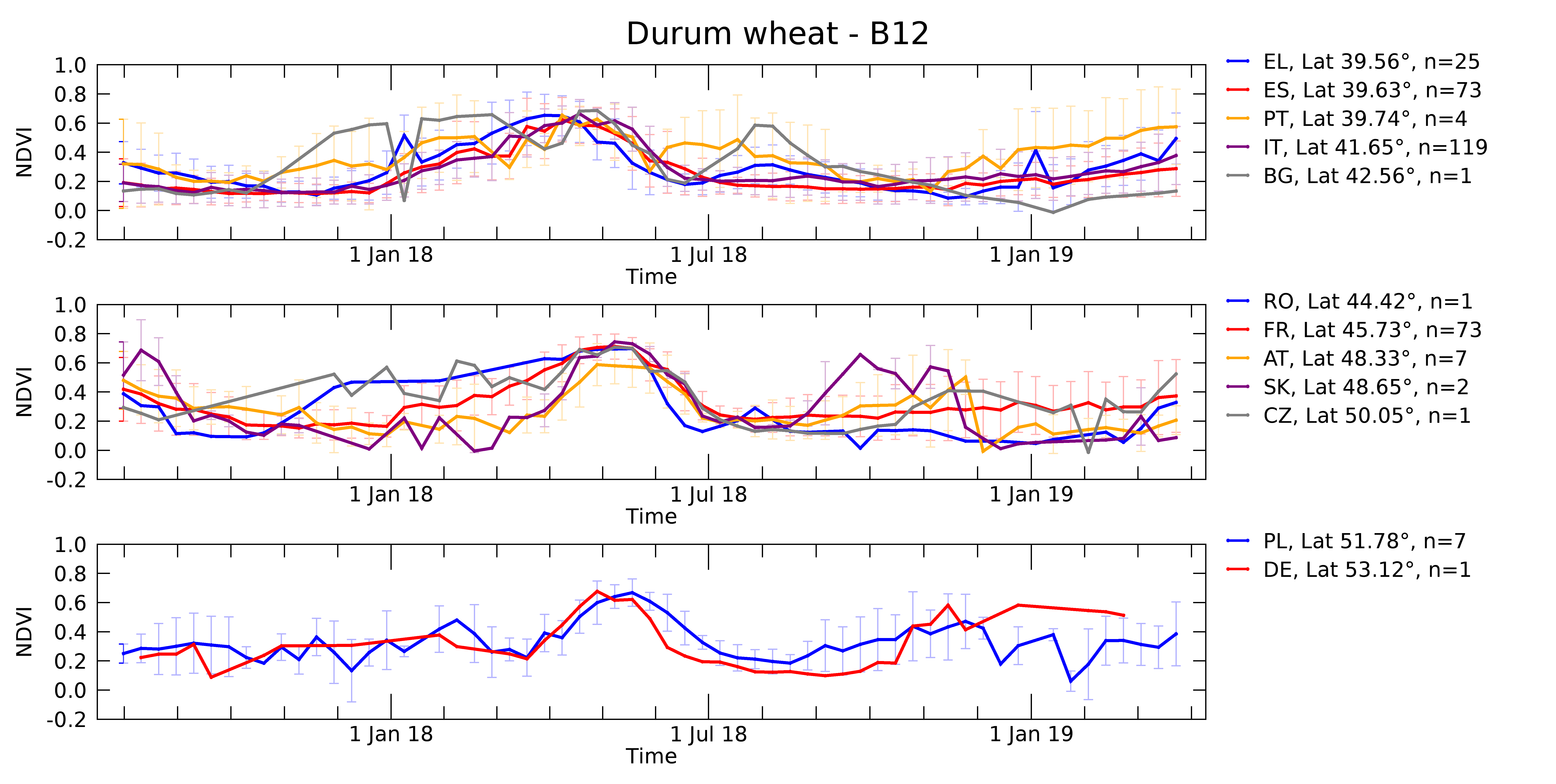** | **F)**  **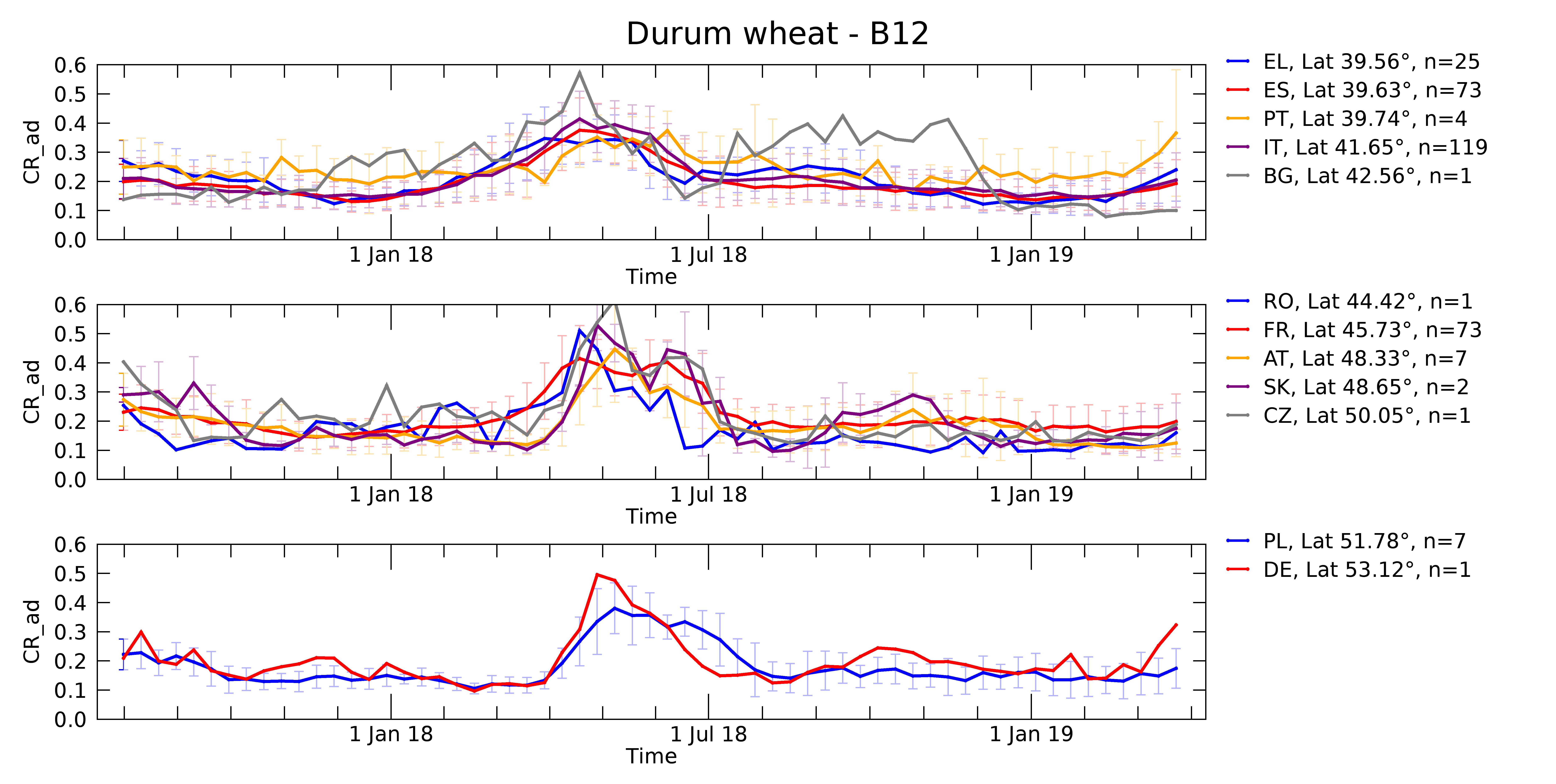** |
| **G)**  **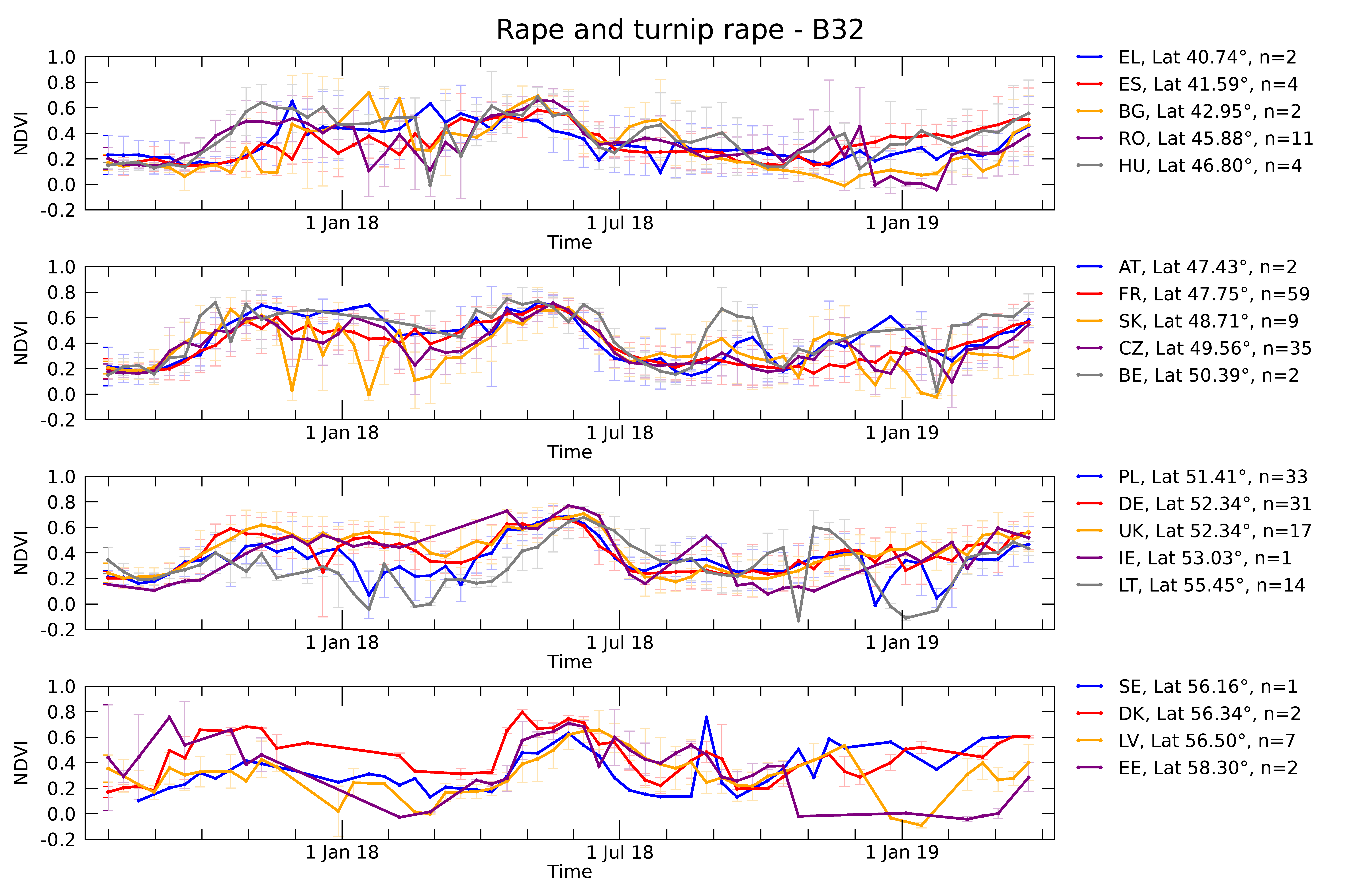** | **H)**  **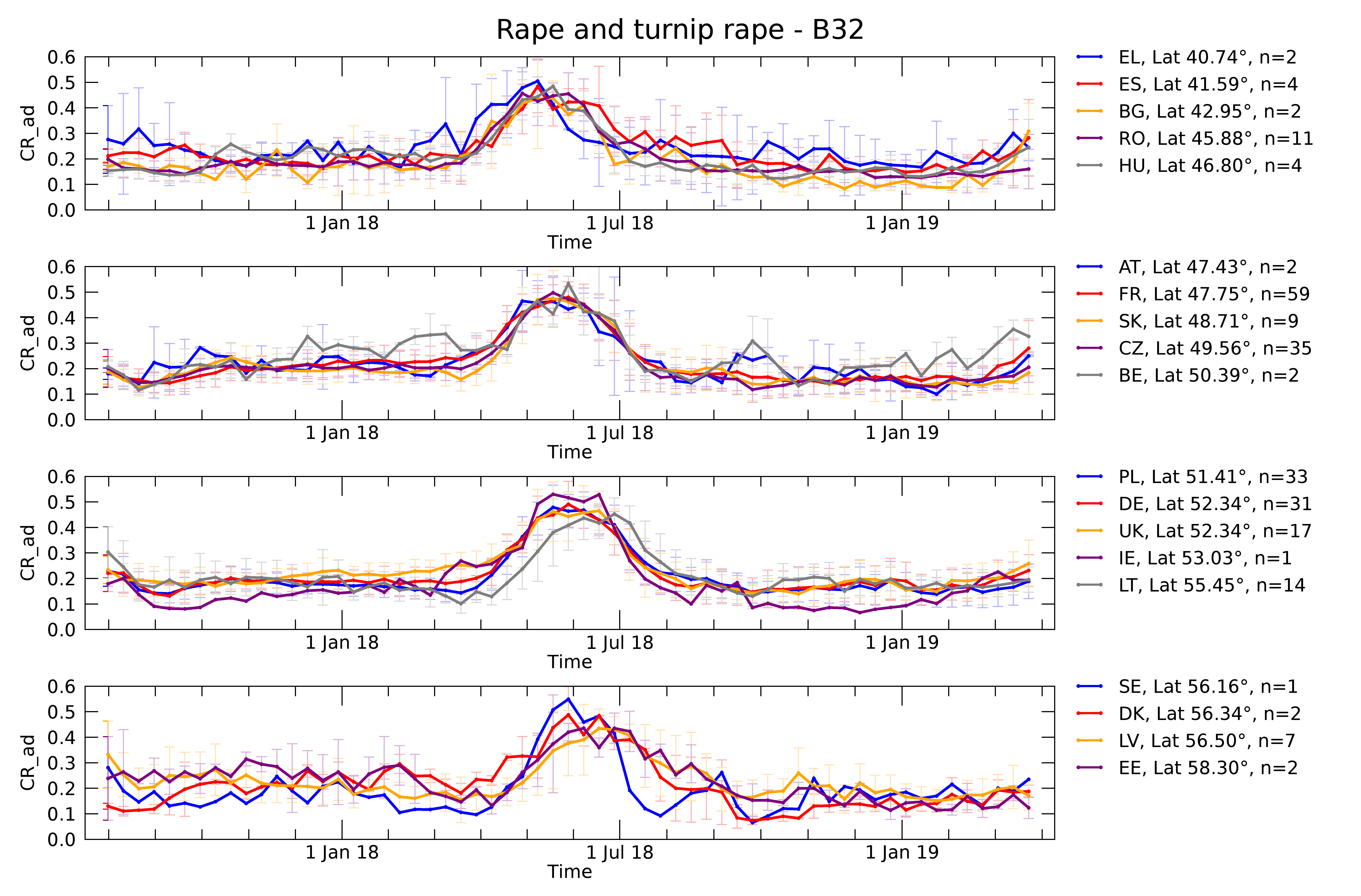** |
| **I)**  **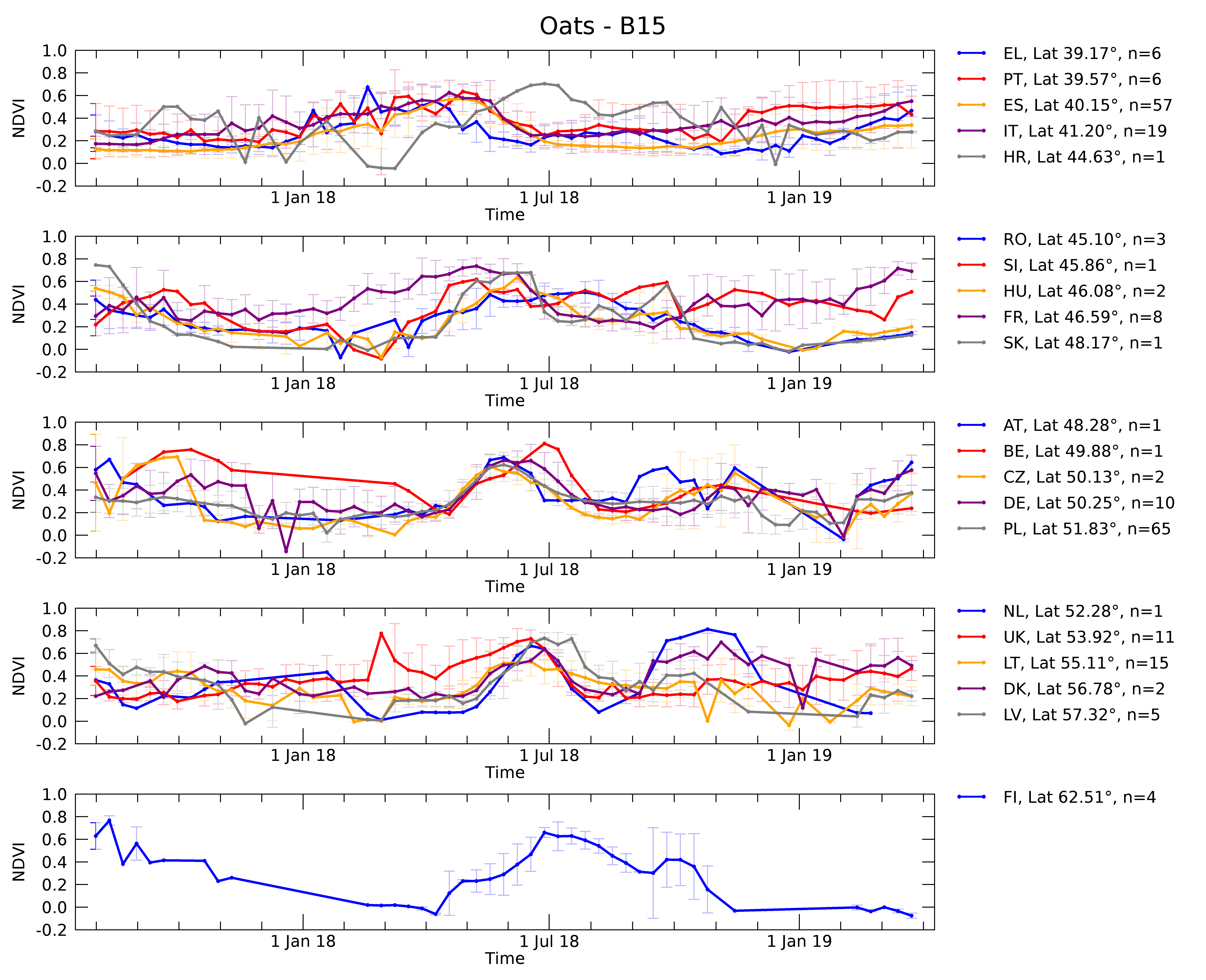** | **J)**  **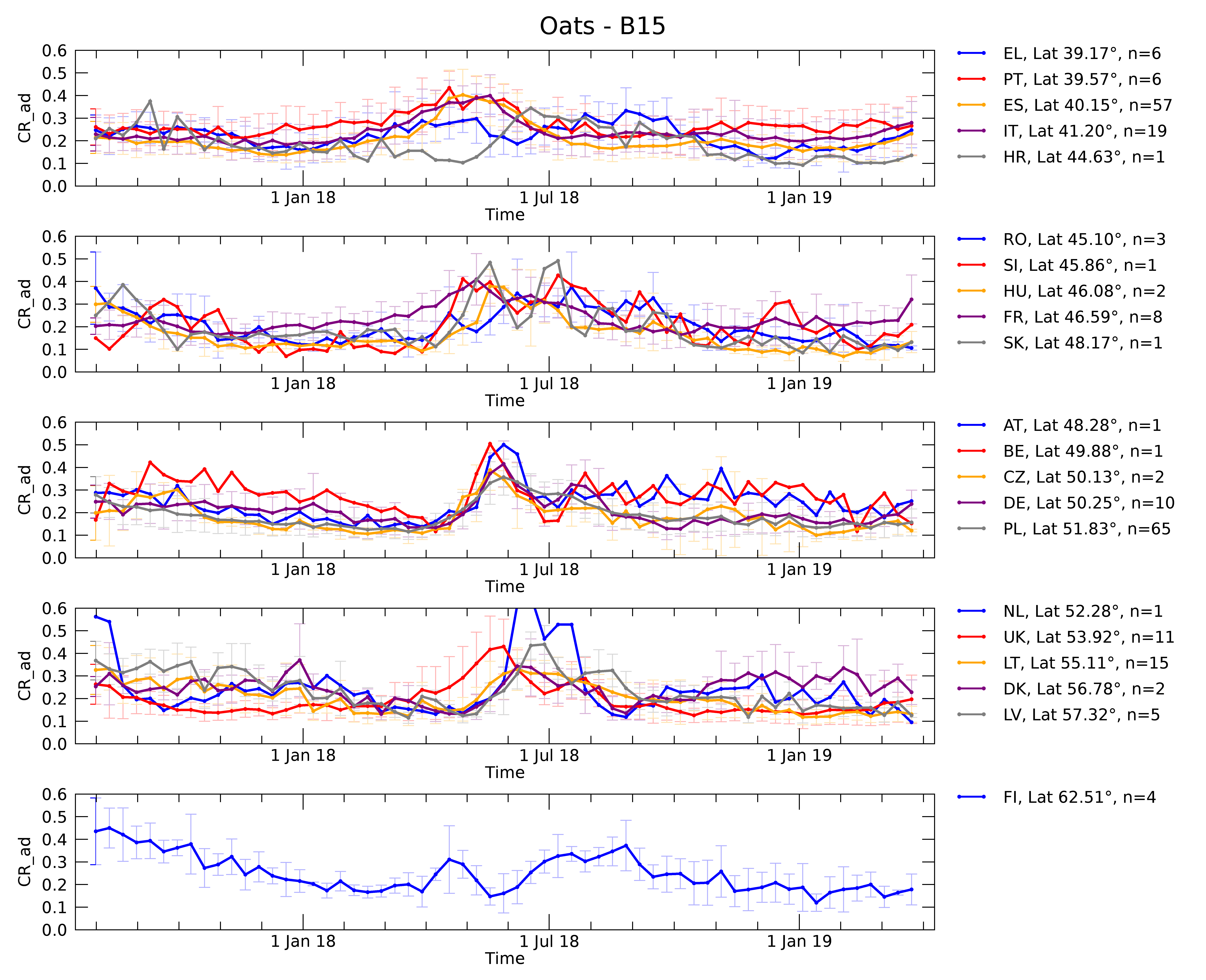** |
| **K)**  **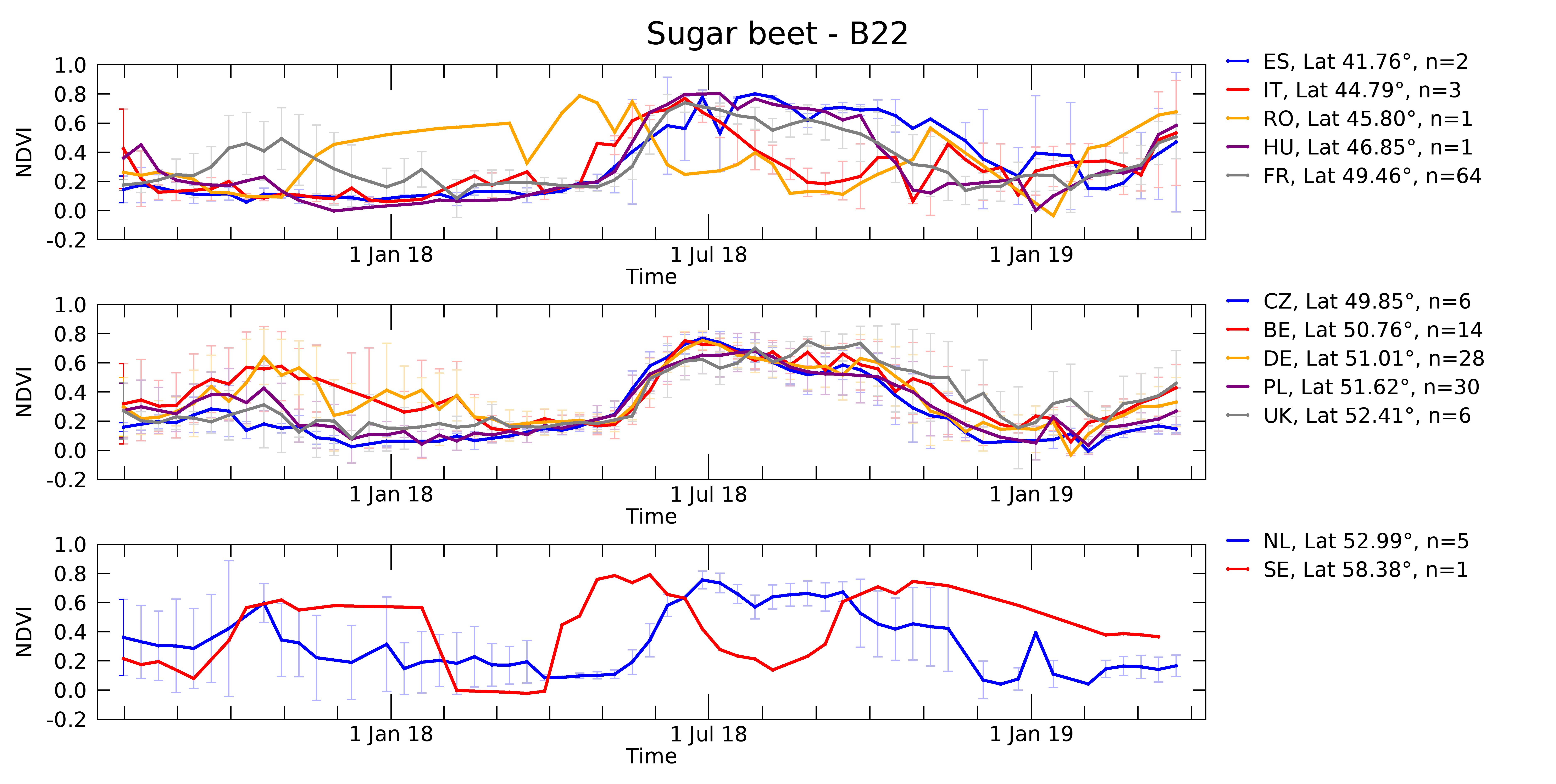** | **L)**  **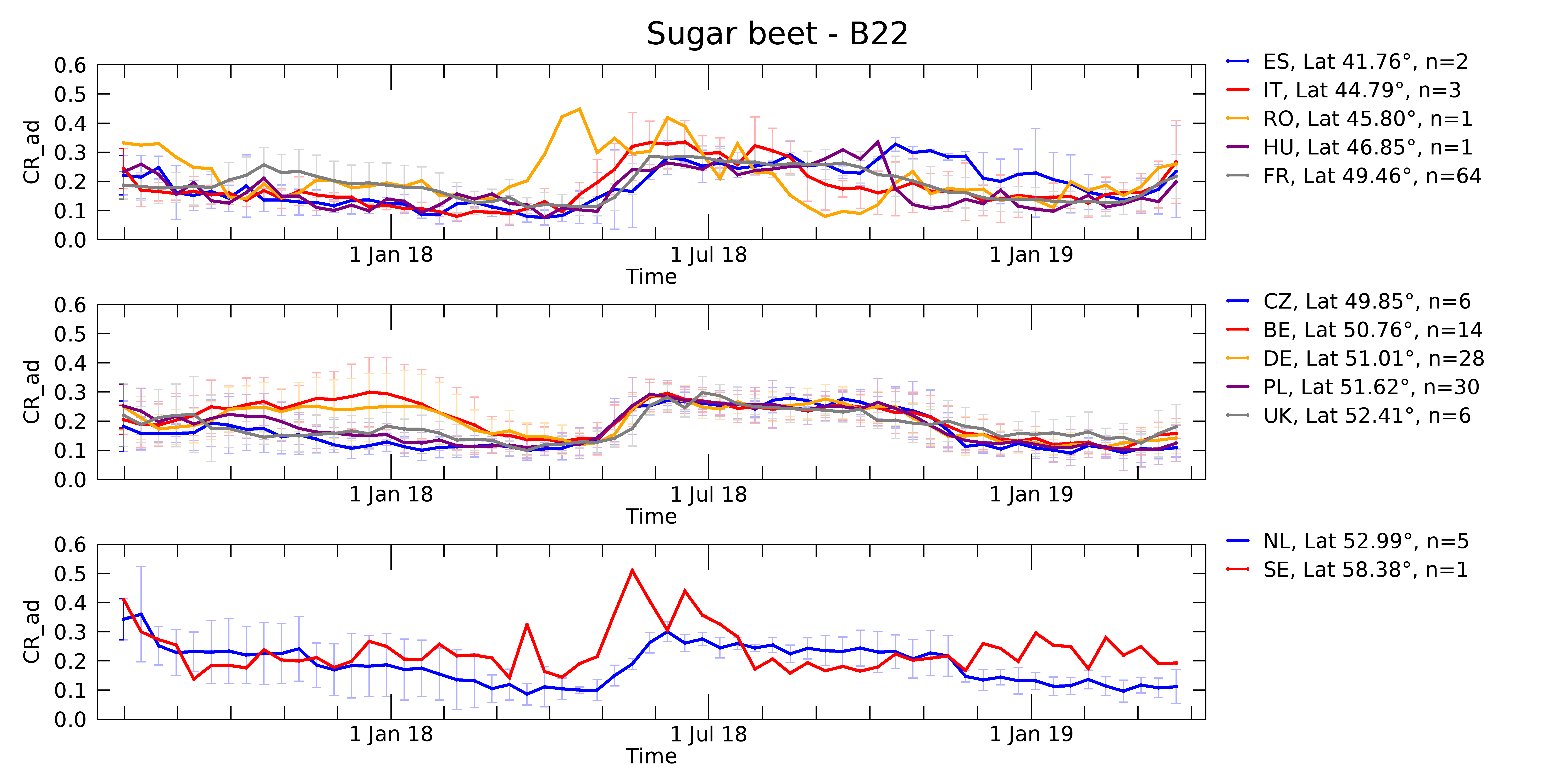** |
| **M)**  **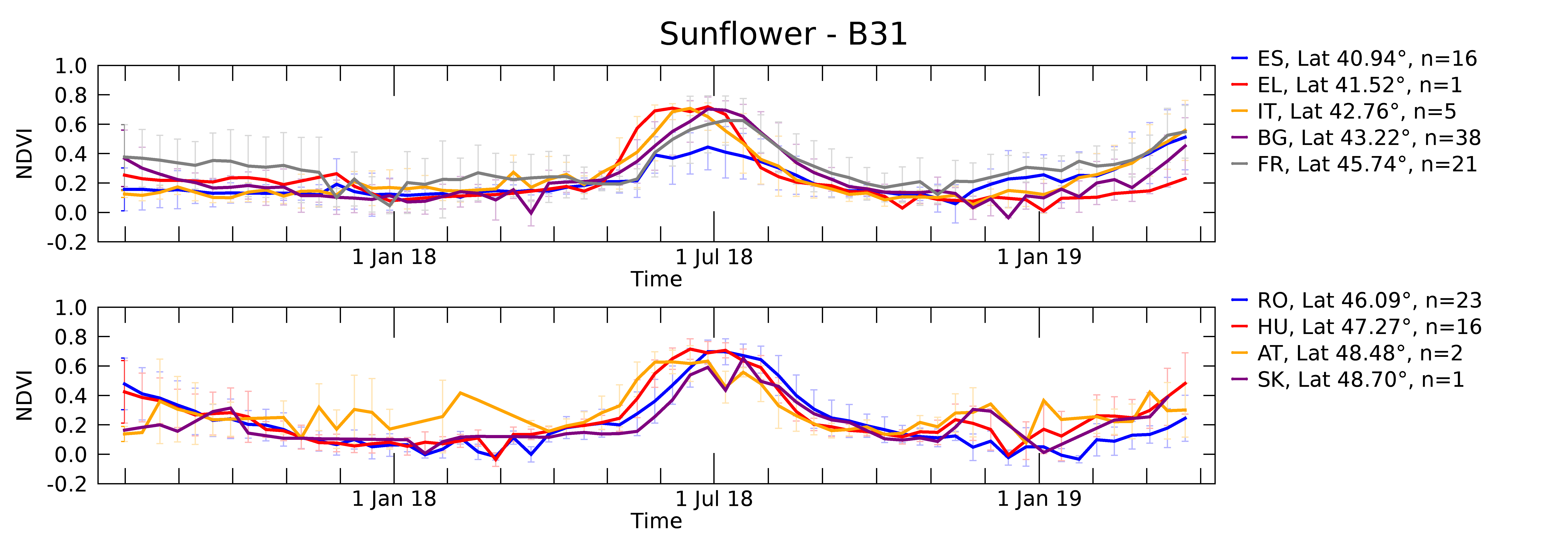** | **N)**  **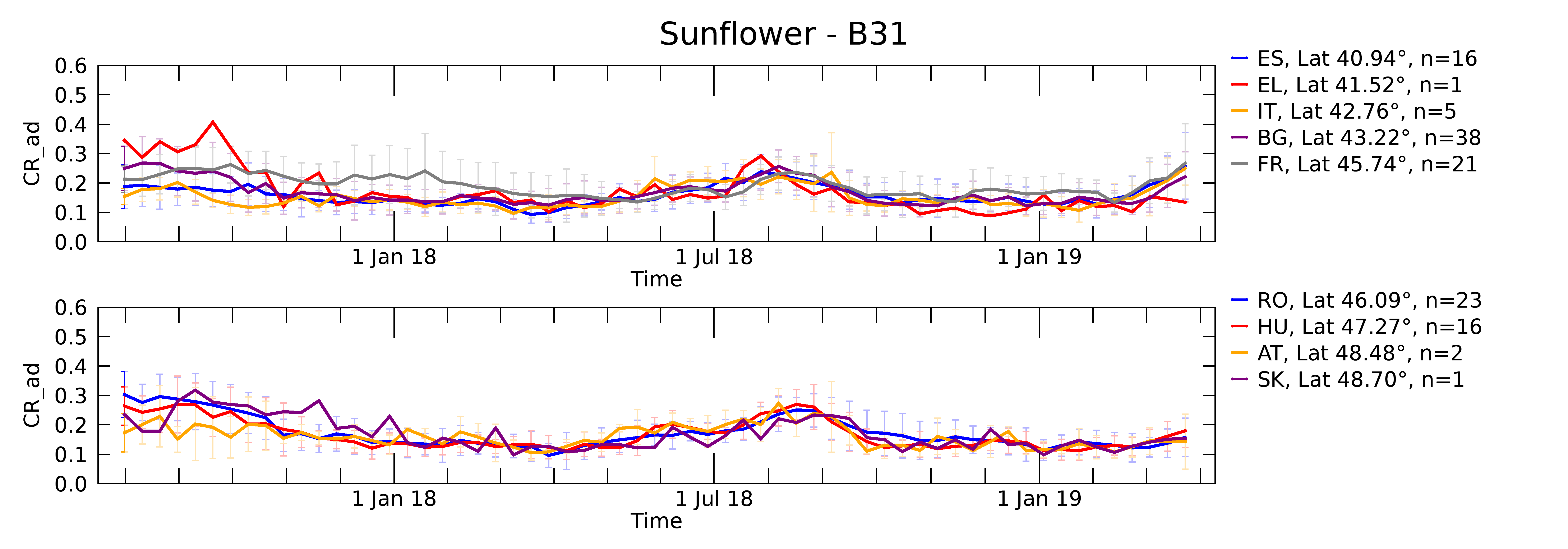** |
| **O)**  **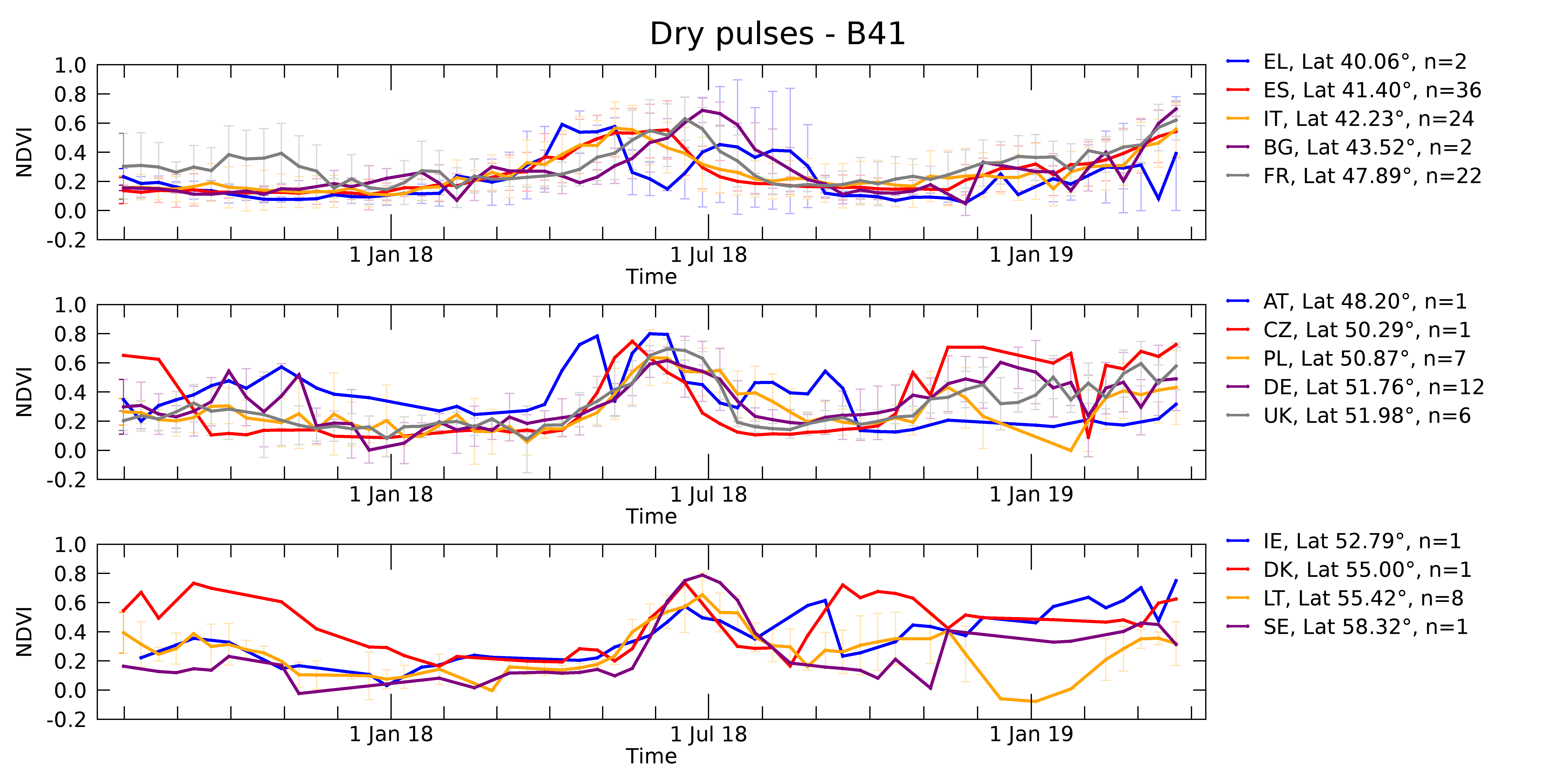** | **P)**  **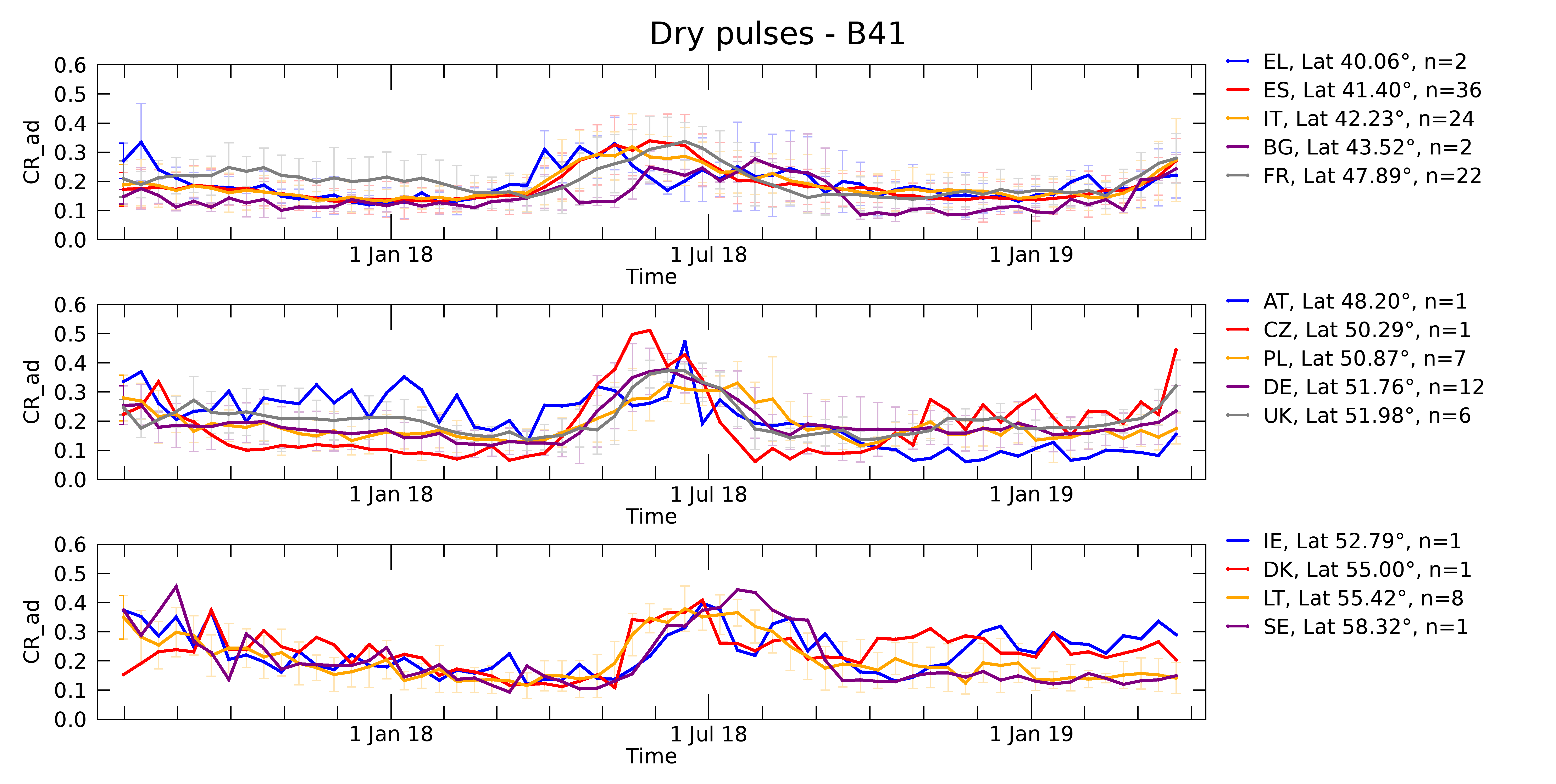** |

*Fig S1. Country-level average temporal profiles of NDVI (left panels) from S2 and CR (right panels) from S1. A, B: barley; C, D: maize; E, F: durum wheat; G, H: rape and turnip rape; I, J: oats; K, L: sugar beet; M, N: sunflowers; and O, P: dry pulses. A single country profile is obtained averaging all polygon-level mean profiles in the country. Mean profiles with irregularly gridded observations were composited at regular 10-day interval using the mean to produce these graphs. Error bars refer ± 1 SD. Countries are ordered by decreasing latitude (i.e. average latitude of all the polygons within the country), reported in the legend with the number of polygons per country.*

| **A)**  **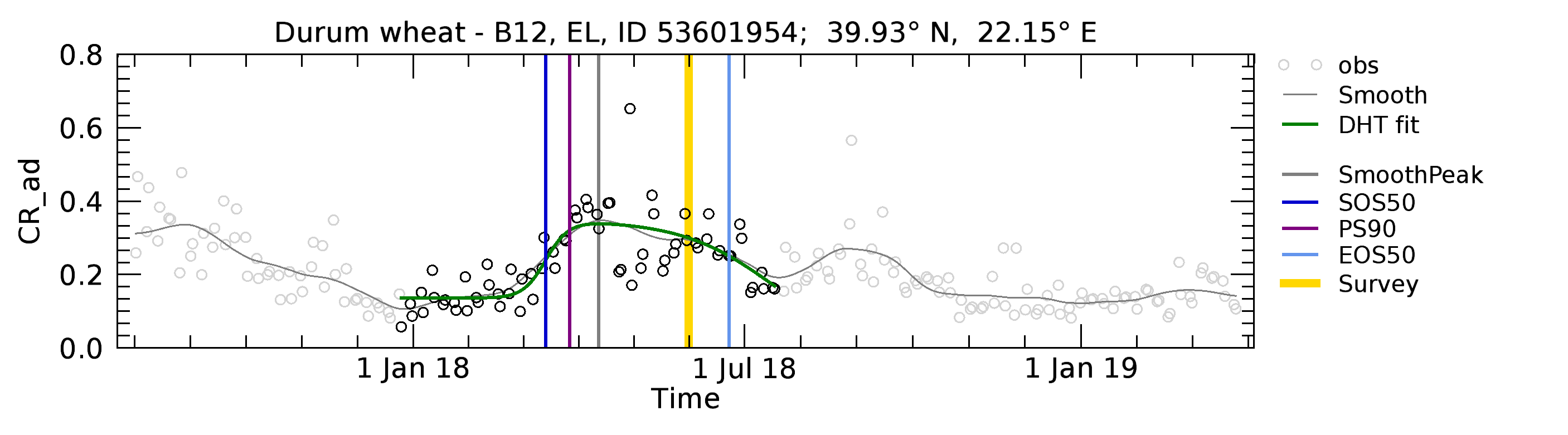** |
| --- |
| **B)**  **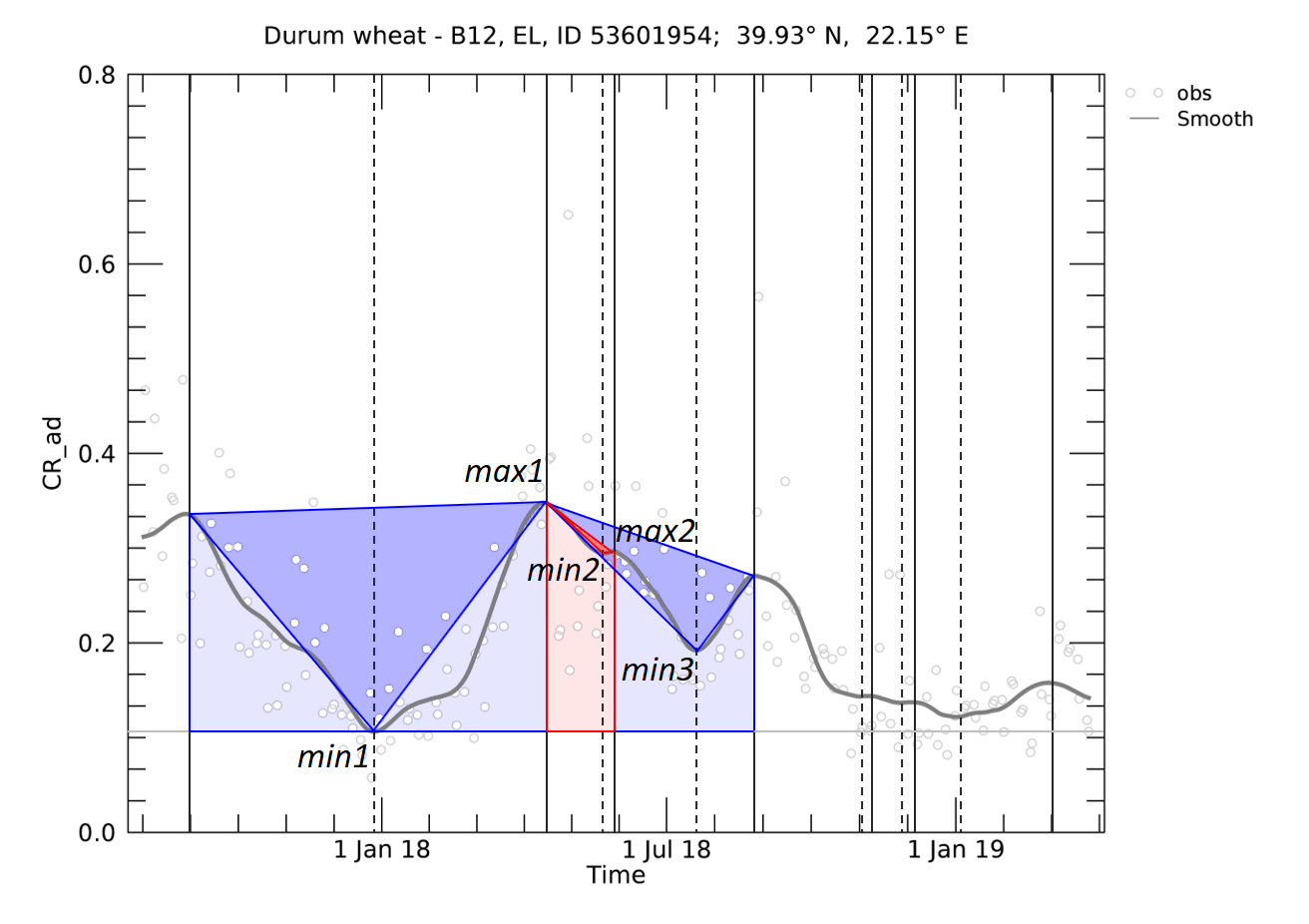** |

*Fig. S2. Example of temporal domain identification. A: CR temporal profile for a durum wheat polygon in Greece, n = 12.6 (for a description of graph elements refer to Fig. 3.). B: CR data points (circles) and smoothed curve (grey line) as in A. Vertical lines identify local extrema of the smoothed curve (solid lines for maxima, dashed lines for minima). As an example of the procedure described in point 4 of Section 3.2.1, the figure shows the process to identify the minima related to the maximum occurring in April (max1). Dark triangles connect two maxima and the minimum in between (area B in Section 3.2.1). The quadrilaterals made by the dark and light areas represent the area below the segment that connects the two maxima and above the overall VI minimum (area A in Section 3.2.1). Blue areas refer to retained minima whereas the red area refers to a discarded minimum. The minimum min1 to the left of max1 is retained because the area ratio B/A is larger than 0.15. On the contrary, min2, the closest minimum to the right of max1, is discarded because the area ratio B/A is smaller than 0.15. As consequence, max2 is discarded as well. Afterwards, min3 is analysed and retained because satisfying the area ratio criterion. As a result, the time period between min1 and min3 is retained as a potential candidate for the time domain and processed further (point 5 and 6 of Section 3.2.1). In this example, the period identified in B is used as temporal domain for fitting the parametric function as shown in A.*

Table S2. Double hyperbolic tangent (DHT) model initial parameters and constraints. When different values are used for NDVI and CR, this is indicated in the table. The smoothed curve refers to the Savitkzy-Golay temporal smoothing of the data points for the seasonal domain of the crop (as described in Section 3.2.1).

| **DHT Parameter** | **Initial value** | **Lower boundary** | **Upper boundary** |
| --- | --- | --- | --- |
| *a_0_* | Minimum VI value before the time of maximum | 0.0 | 0.8 (NDVI), 0.6 (CR) |
| *a_1_* | Amplitude of the ascending phase of the smoothed curve | 0.05 | Twice the initial value |
| *a_4_* | Amplitude of the decay phase of the smoothed curve | 0.05 | Twice the initial value |
| *a_2_* | Time at which the ascending phase of smoothed curve reaches 50% of the amplitude | First day of the optimisation window | Time at which the ascending phase of smoothed curve reaches 90% amplitude |
| *a_5_* | Time at which the decay phase of smoothed curve reaches 50% amplitude | Last day of the optimisation window | Time at which the decay phase of smoothed curve reaches 90% amplitude |
| *a_3_* | 0.02 | 0.00001 | 0.3 |
| *a_6_* | -0.02 | -0.3 | 0.00001 |

| **A)**  **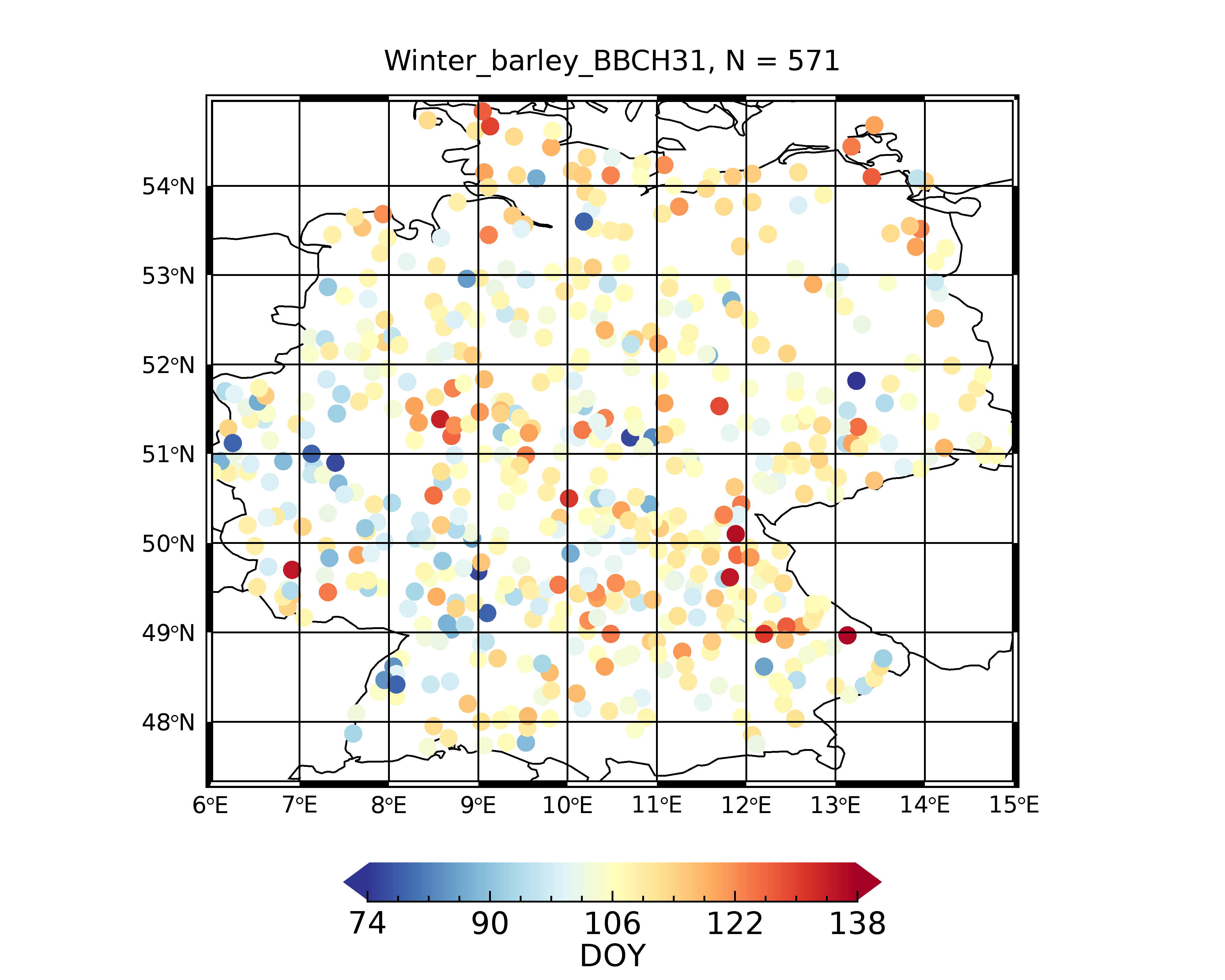** | **B)**  **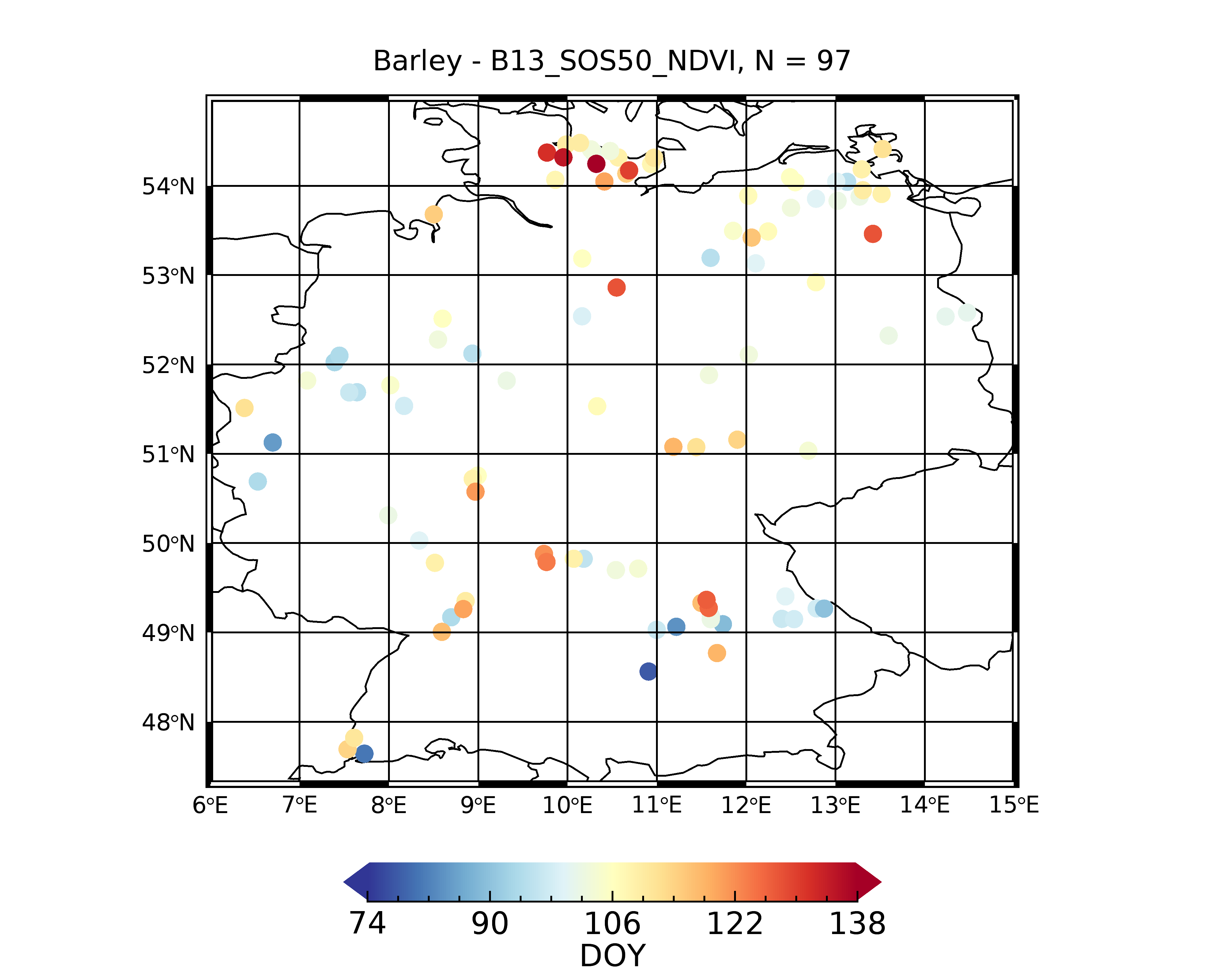** |
| --- | --- |

*Fig. S3. Timing of: A) BBCH stage 31 (early stem elongation) of winter barley according to the DWD phenological database, B) NDVI-retrieved SOS50 for LUCAS-Copernicus barley polygons (thus not differentiating between winter and spring varieties) in Germany.*

*
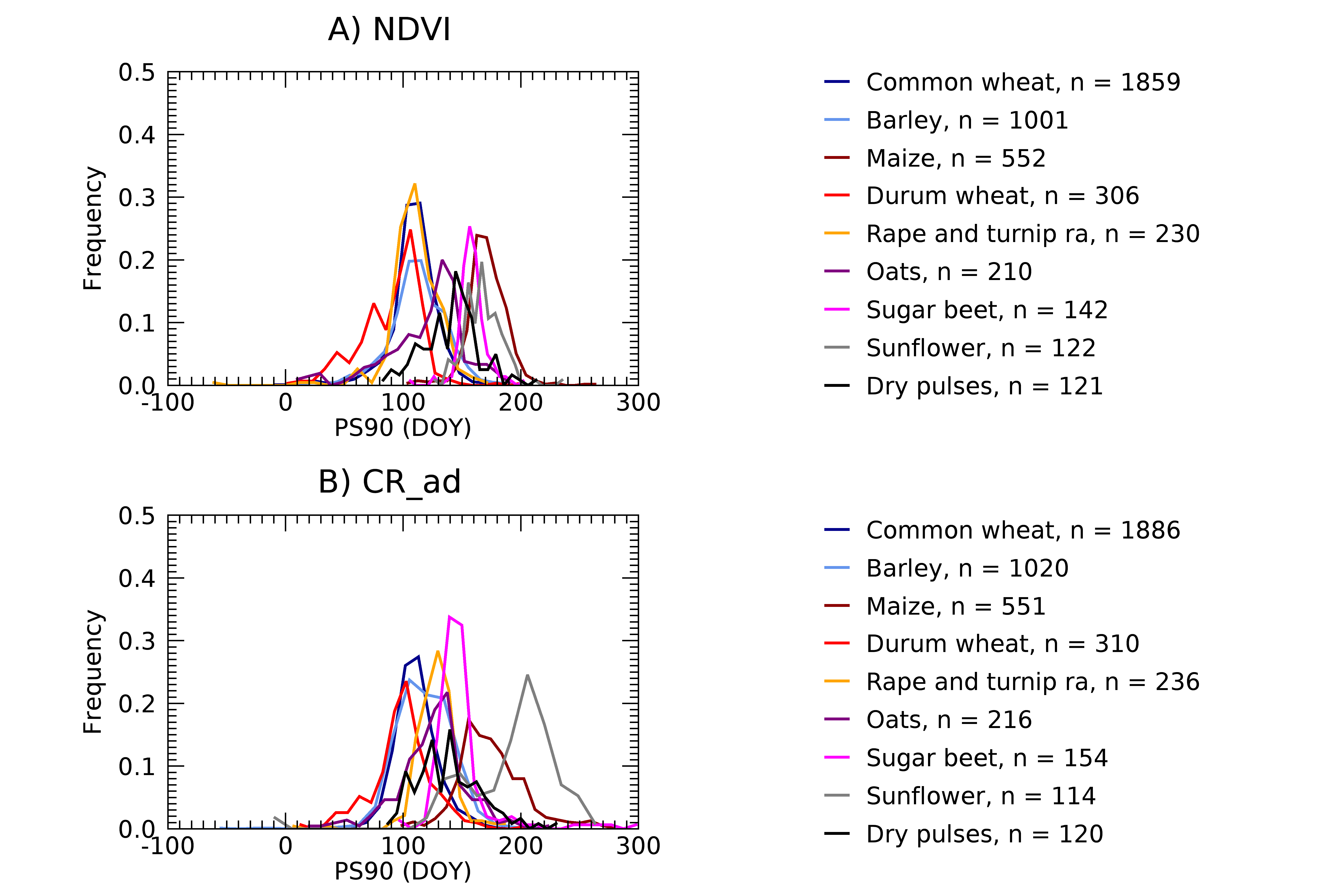
*

*Fig. S4. Frequency histograms of PS90 as derived from NDVI (A) and CR (B). Timings are expressed in day of year (DOY) of 2018, negative values indicate dates in 2017. The sample size (n) per crop type refers to the total number of polygons for which phenology was successfully retrieved using NDVI (A) and CR (B).*

| **Common wheat** | |
| --- | --- |
| **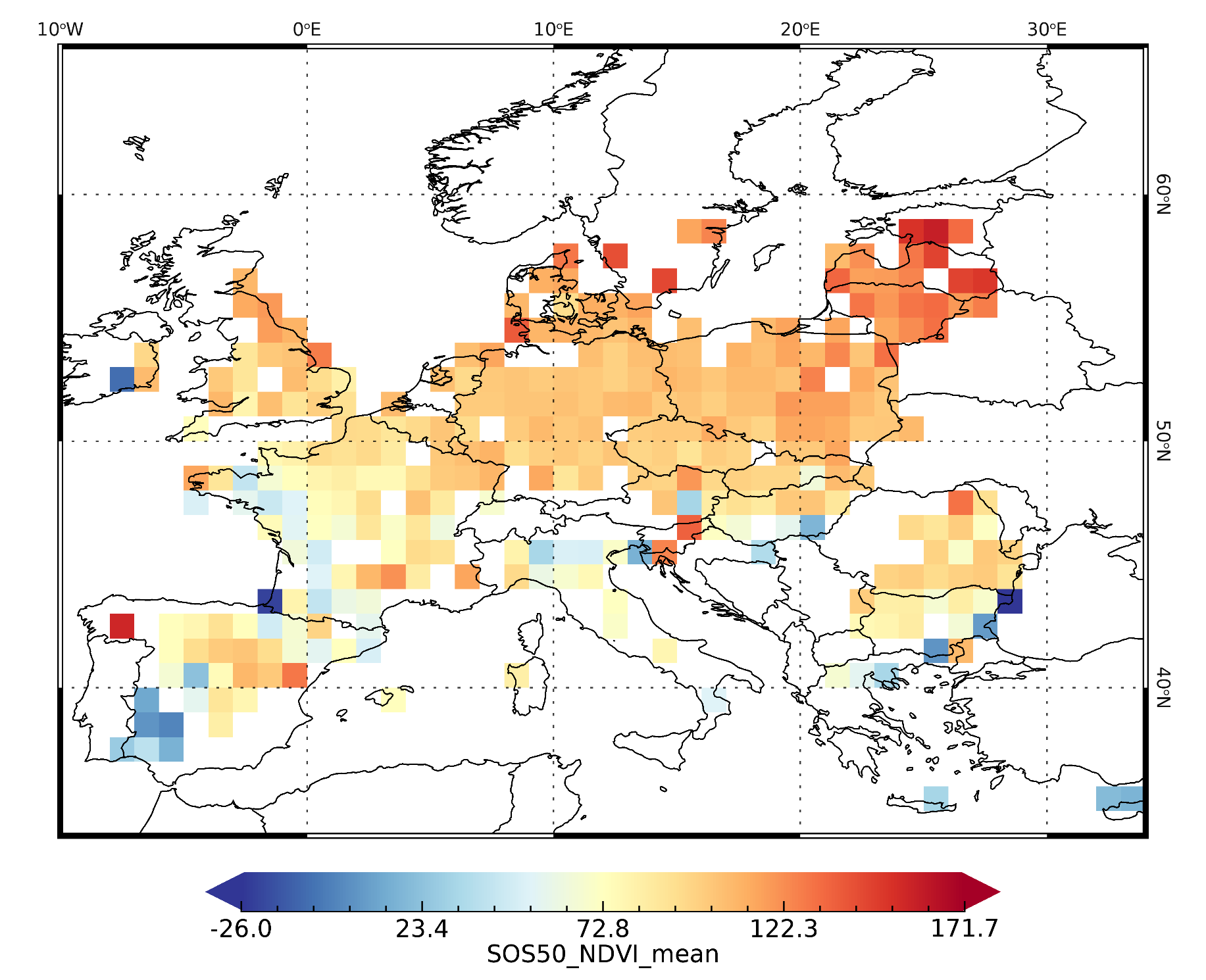** | **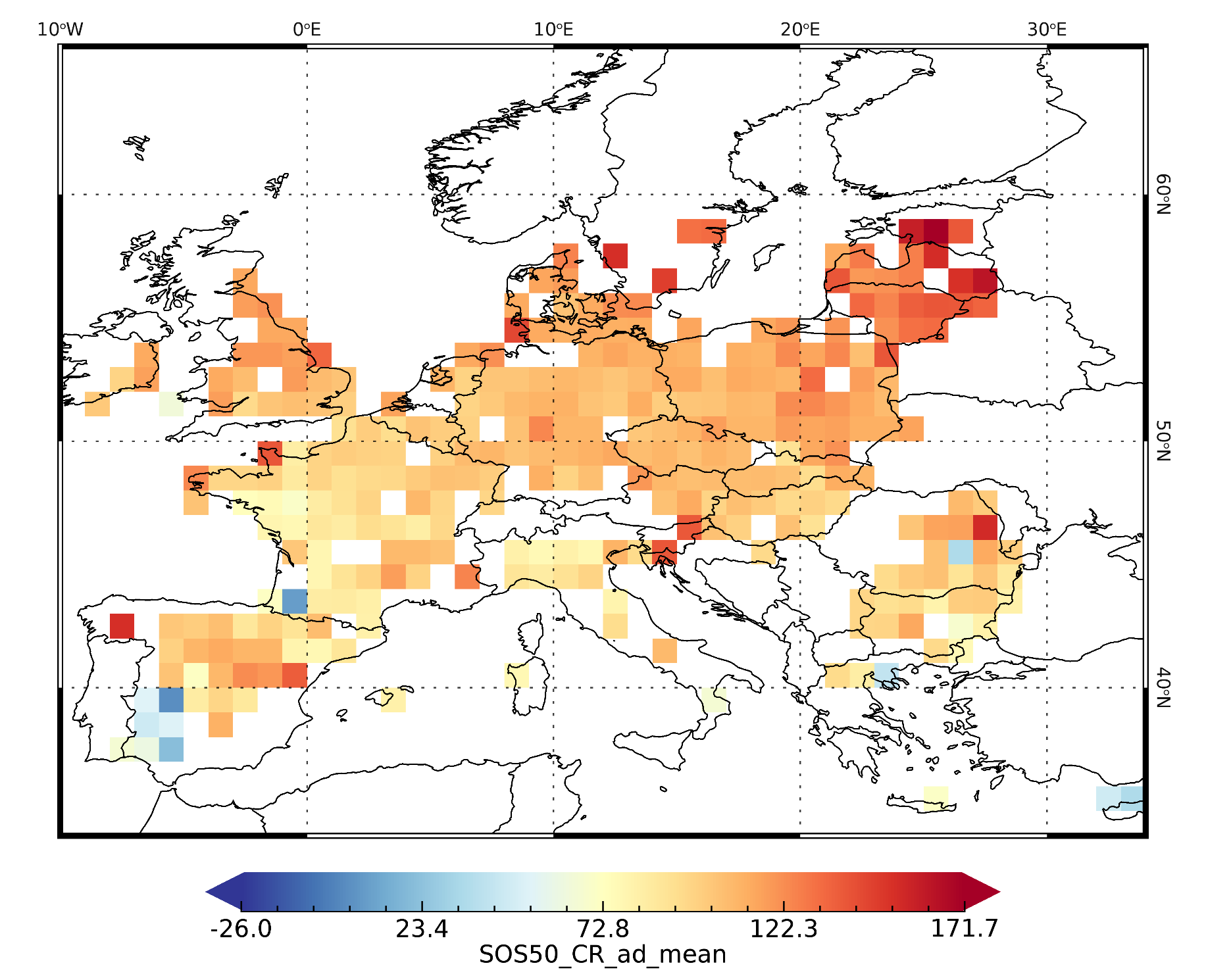** |
| **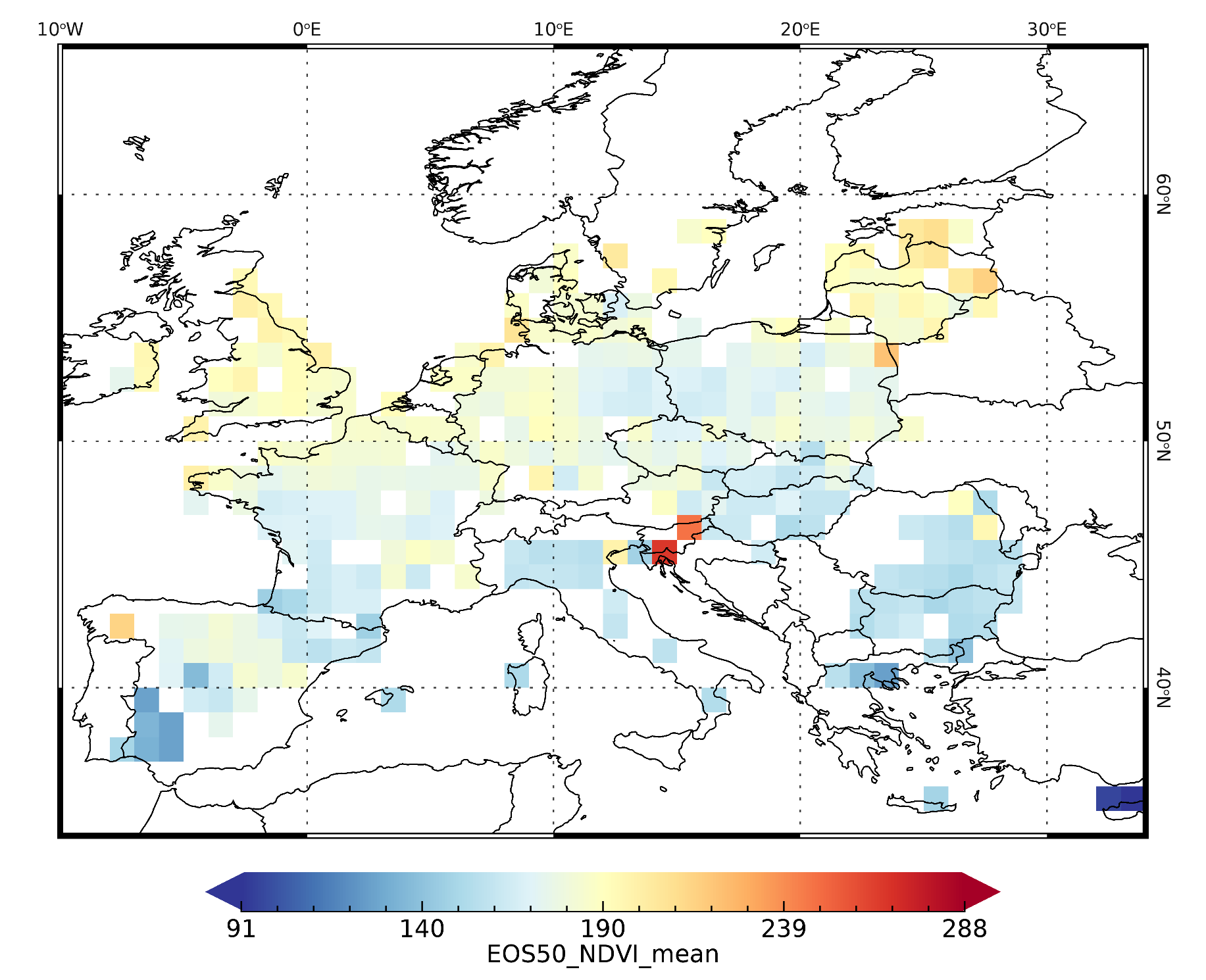** | **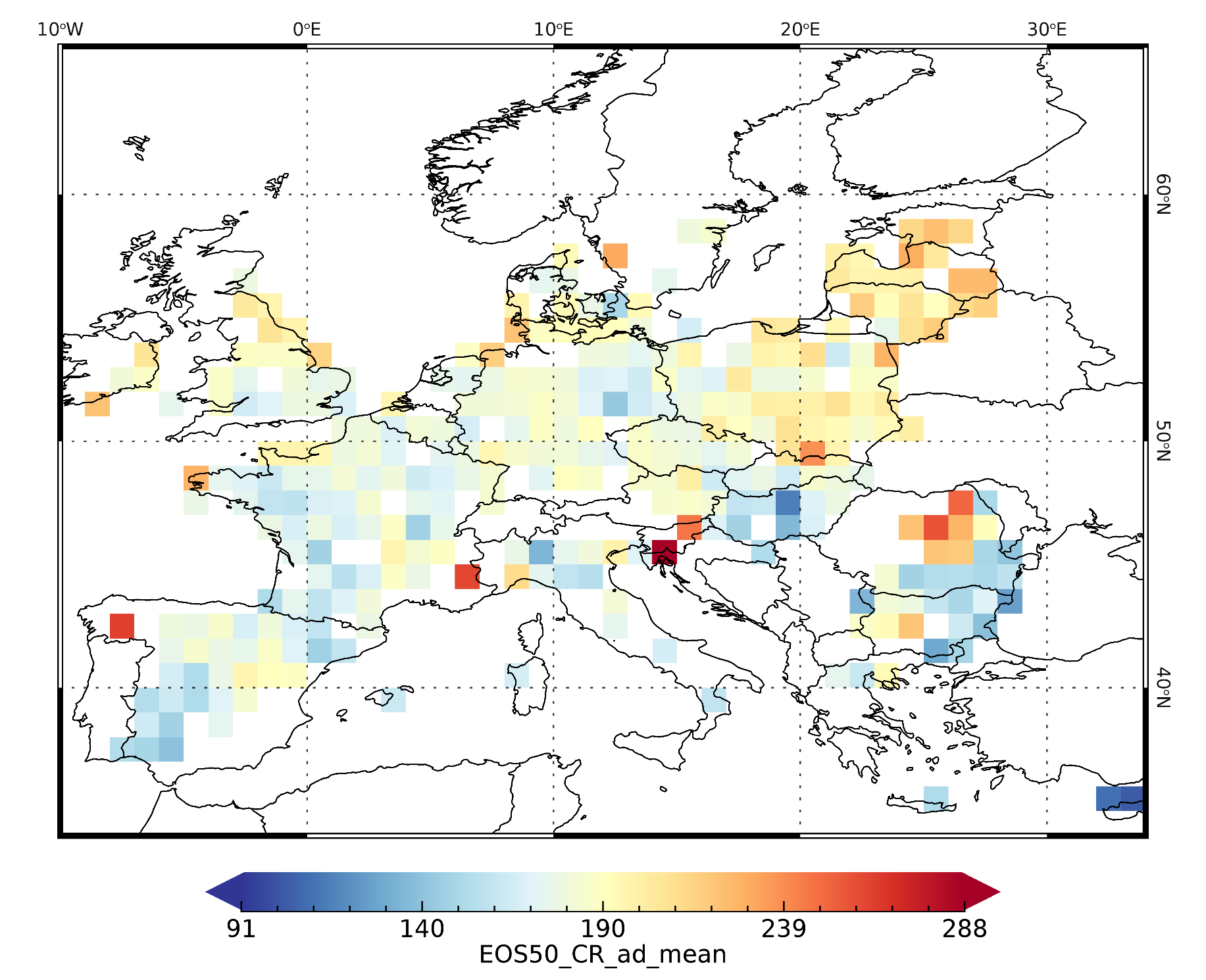** |
| **Maize** | |
| **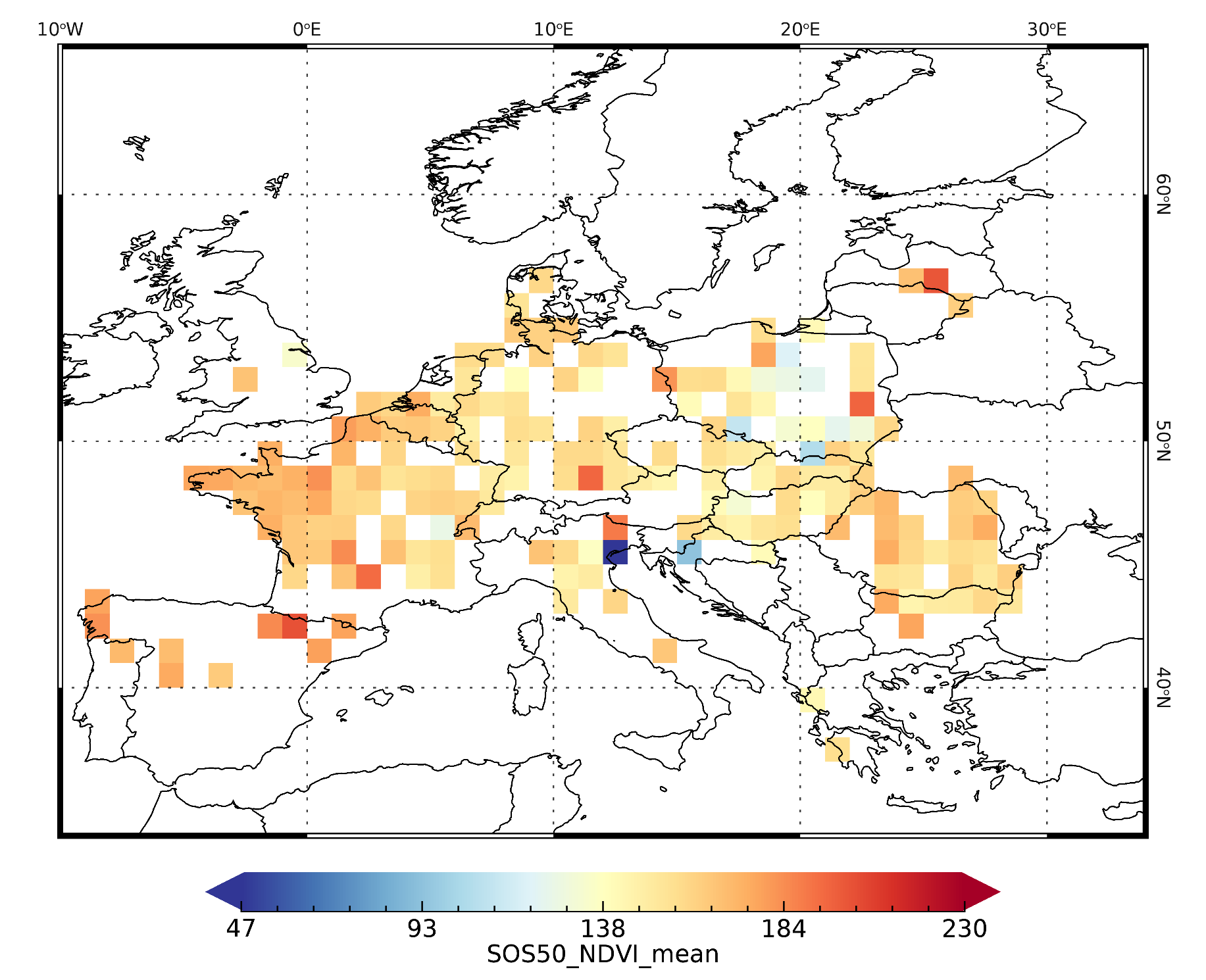** | **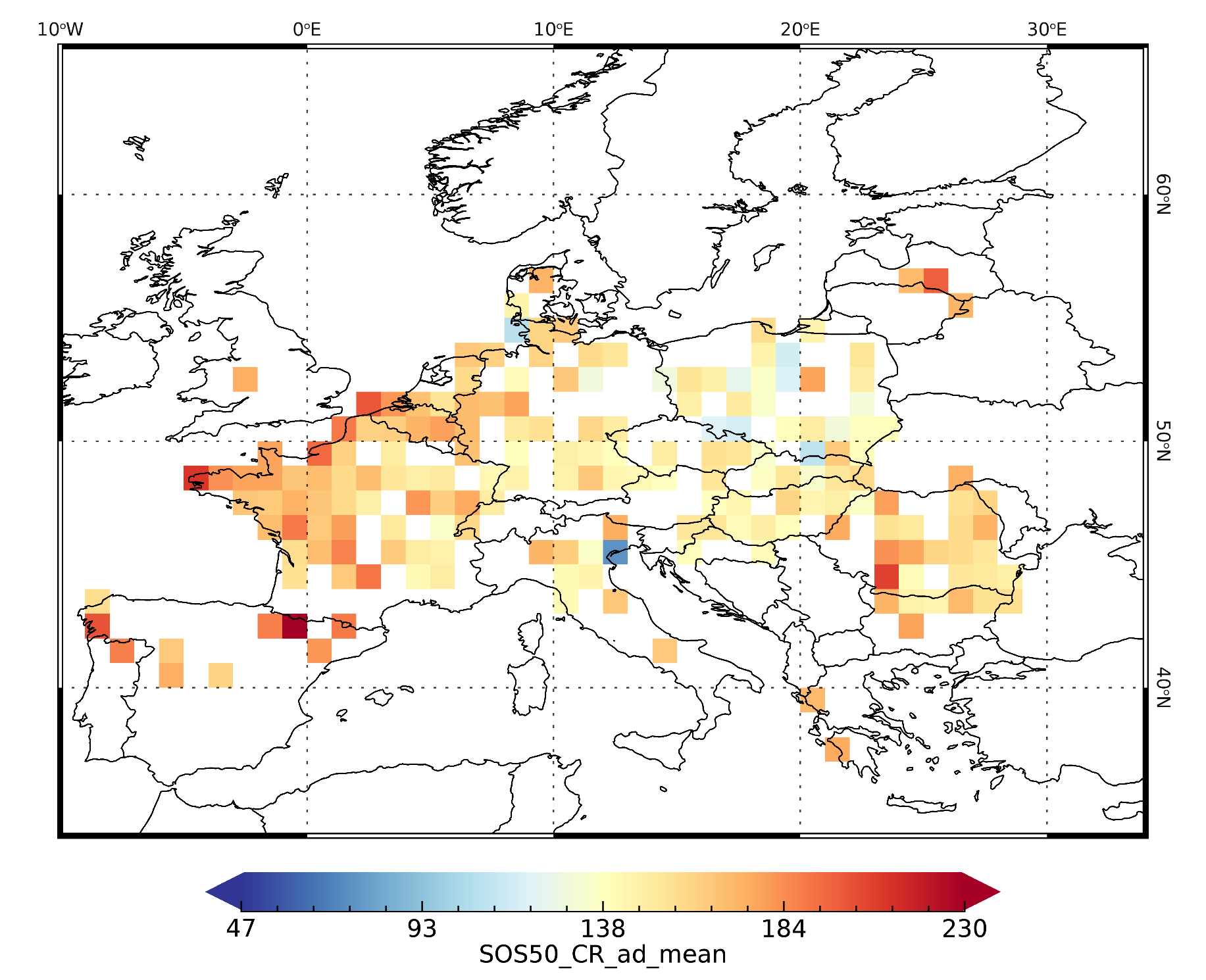** |
| **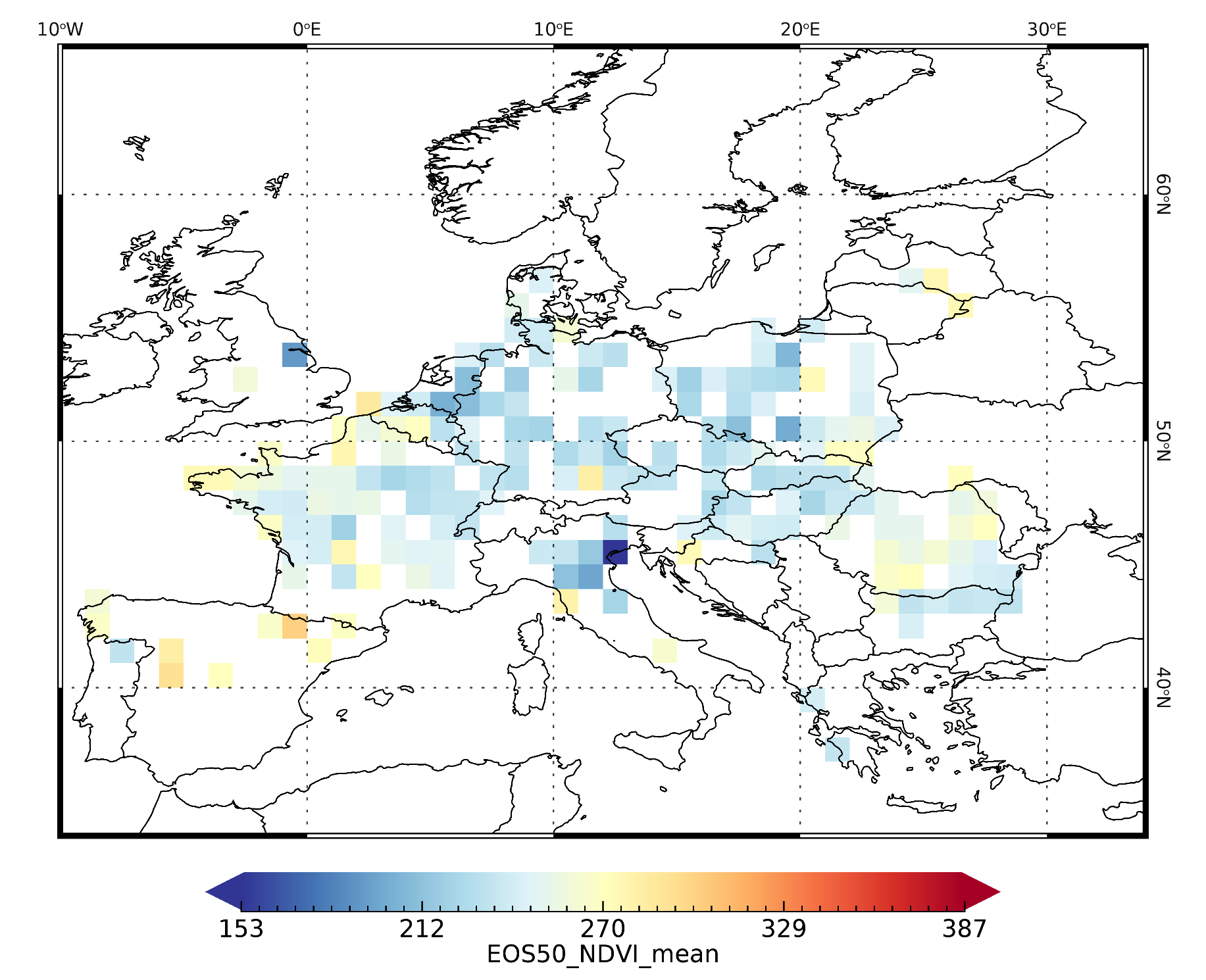** | **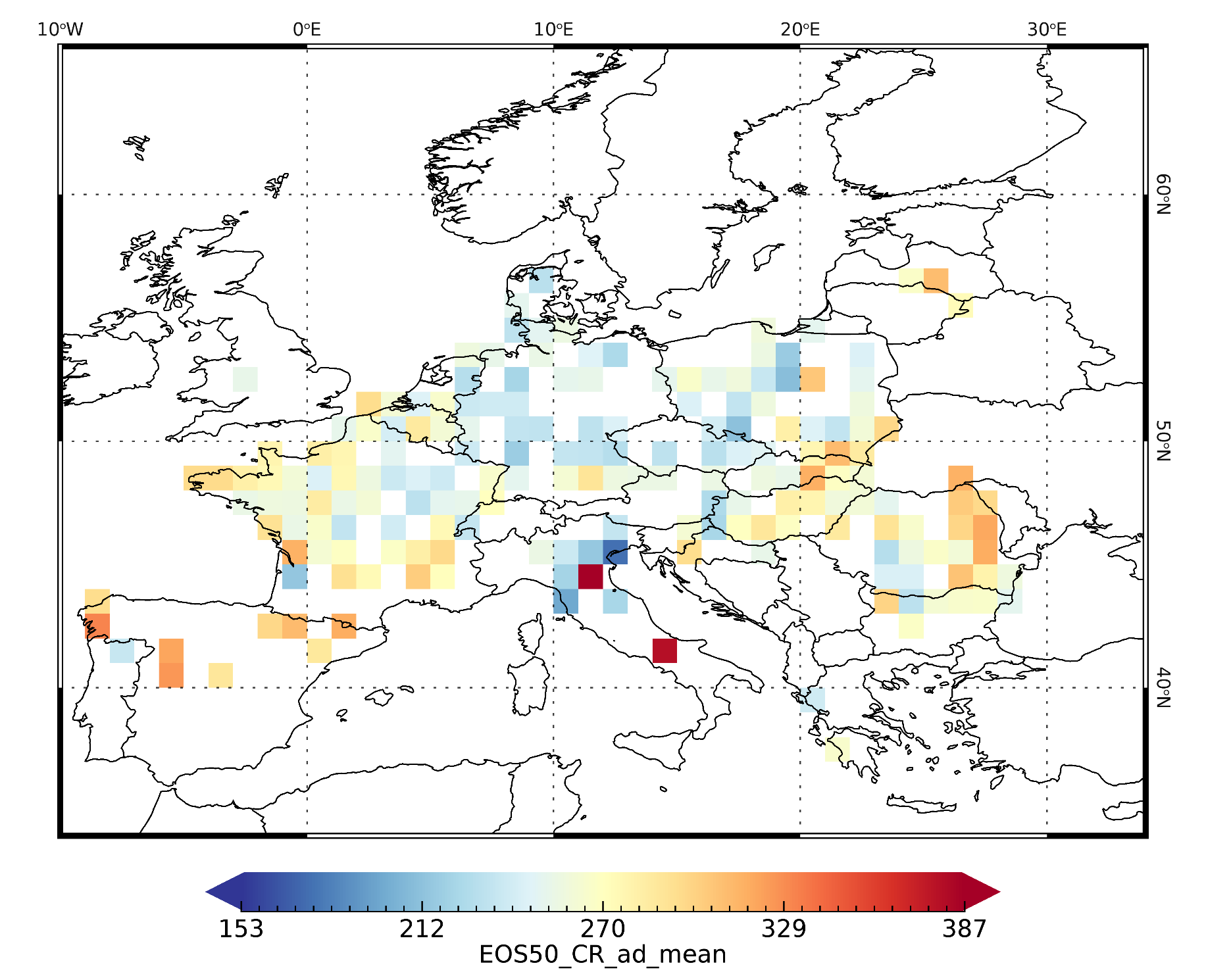** |
| **Durum wheat** | |
| **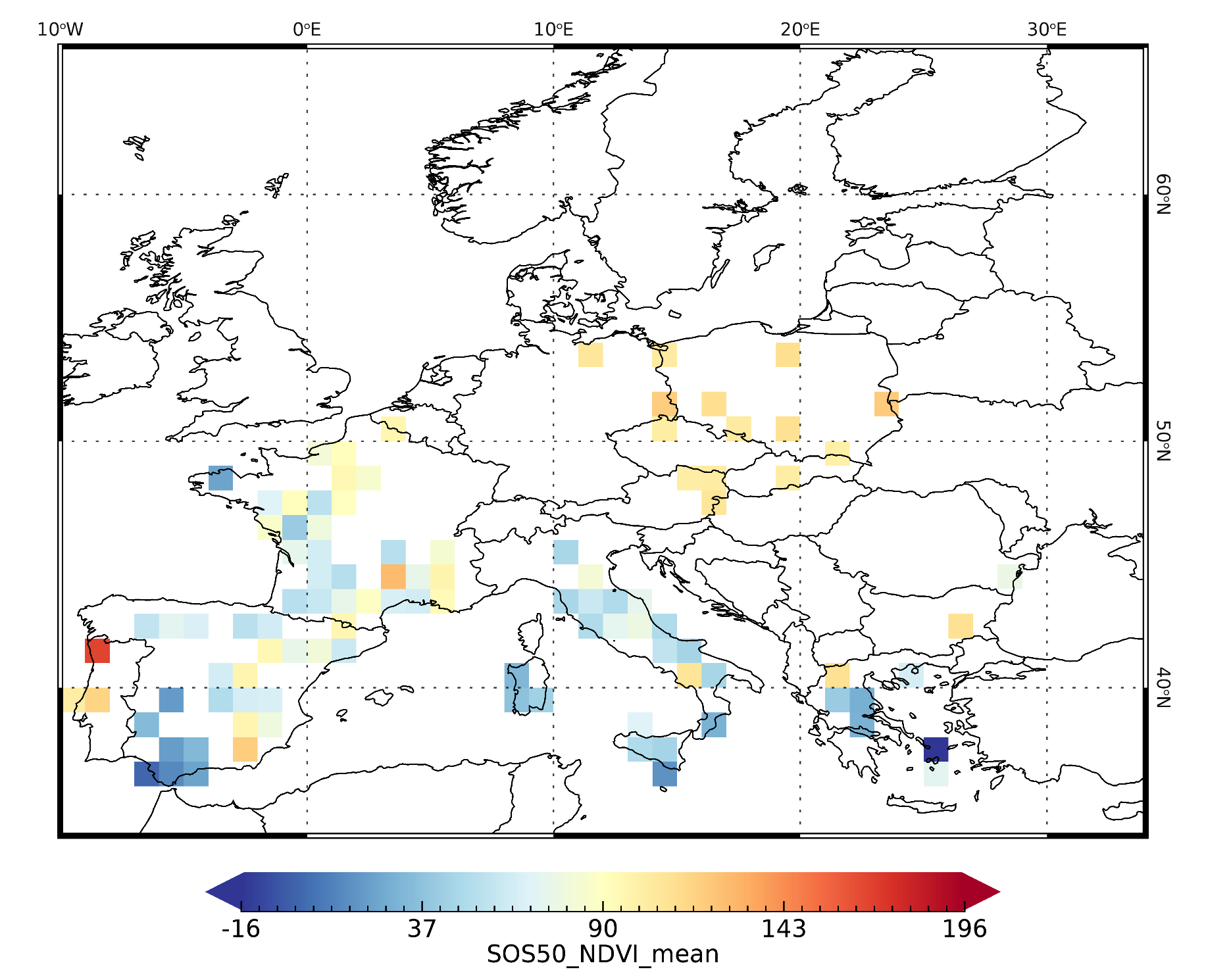** | **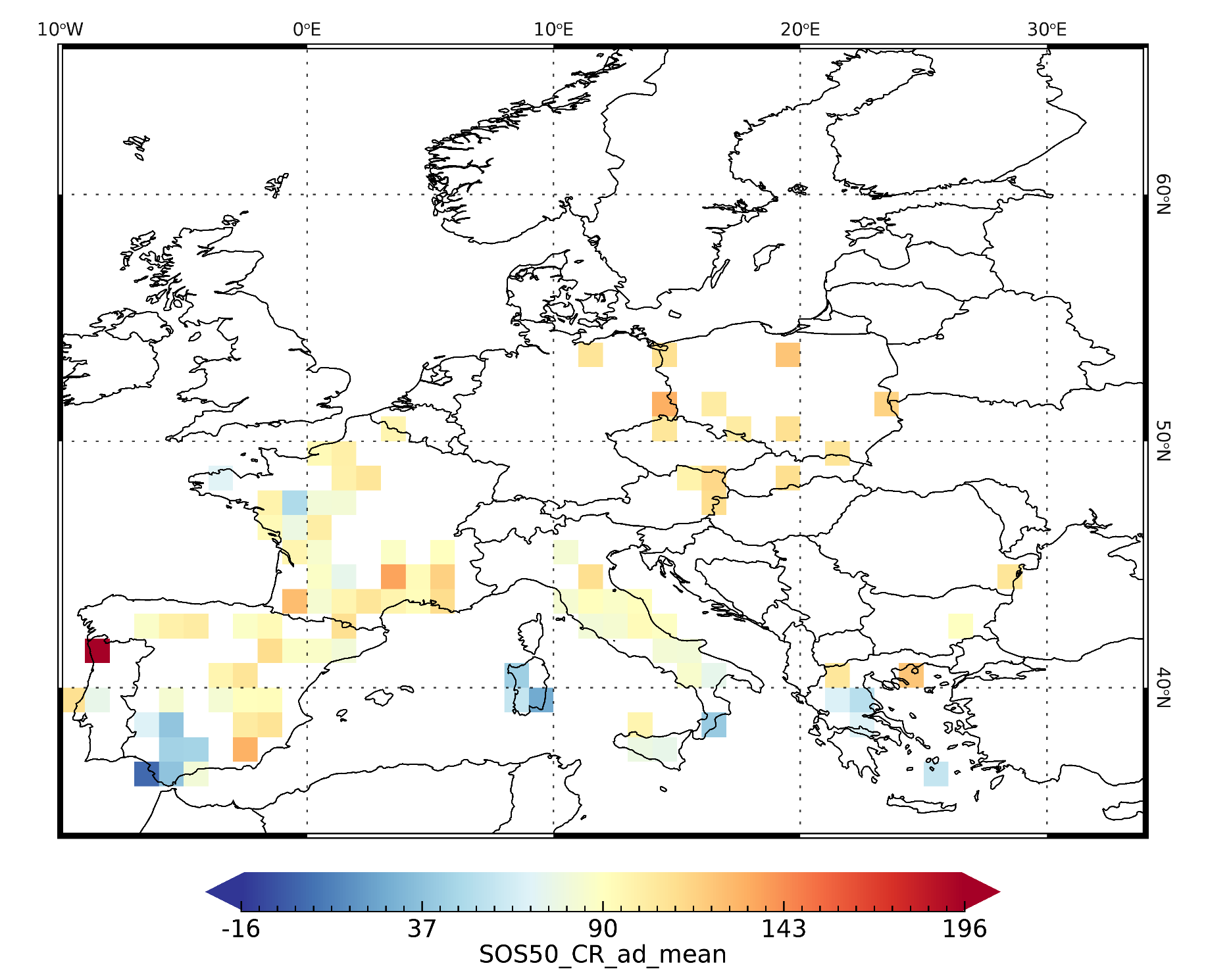** |
| **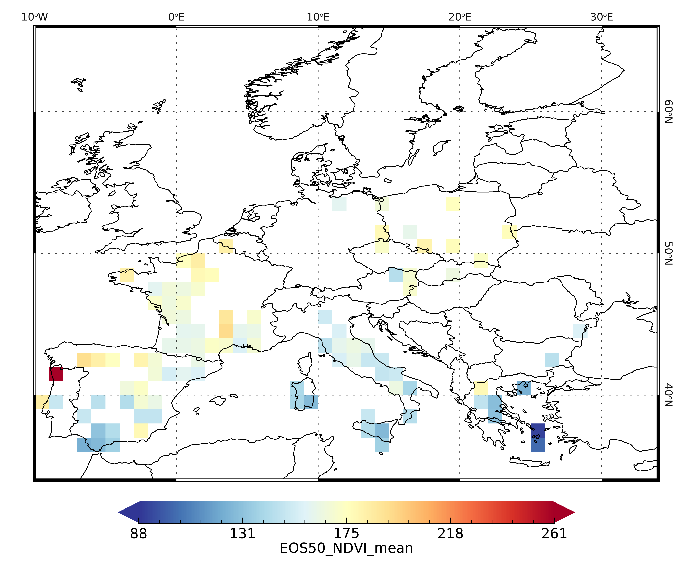** | **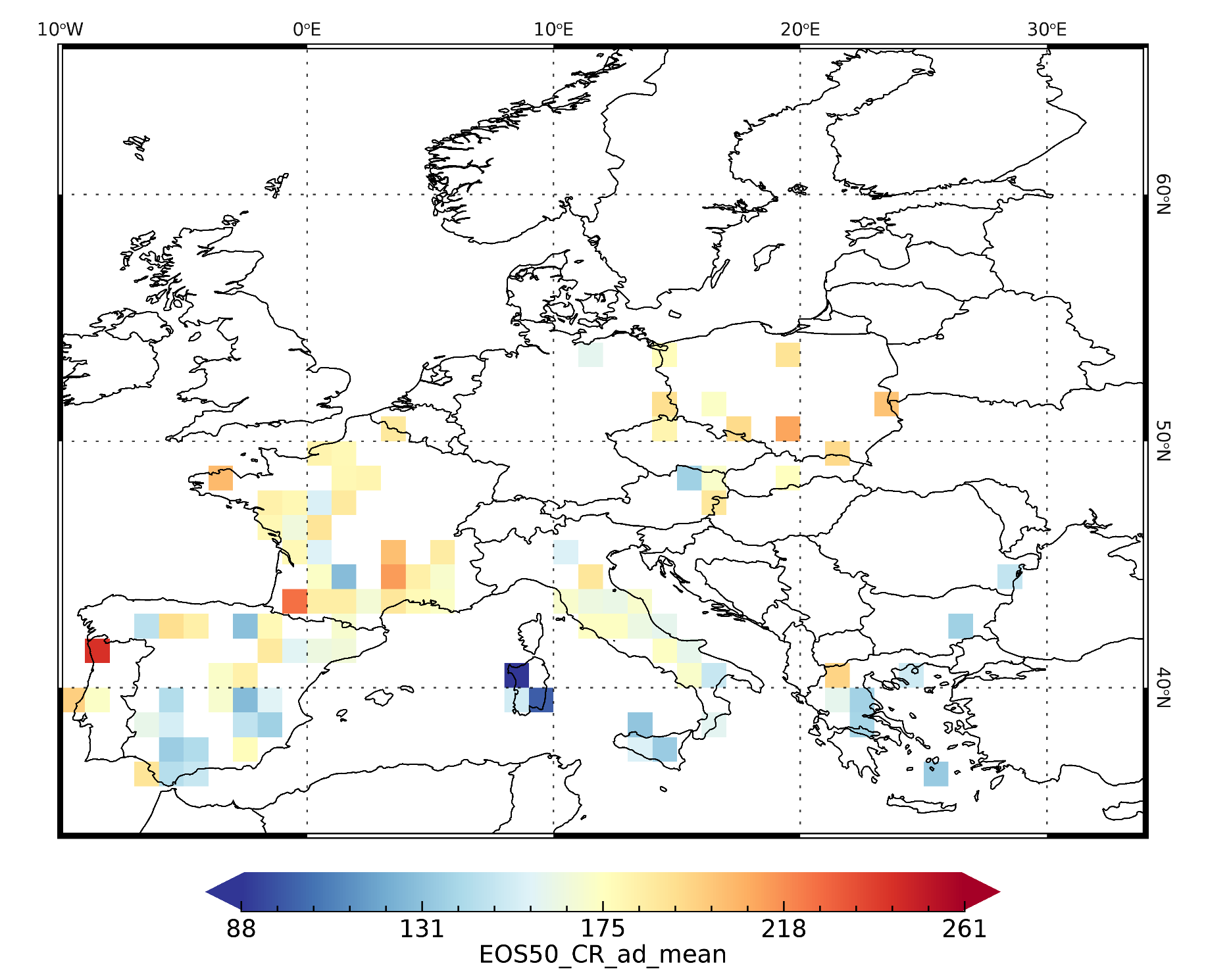** |
| **Rape and turnip rape** | |
| **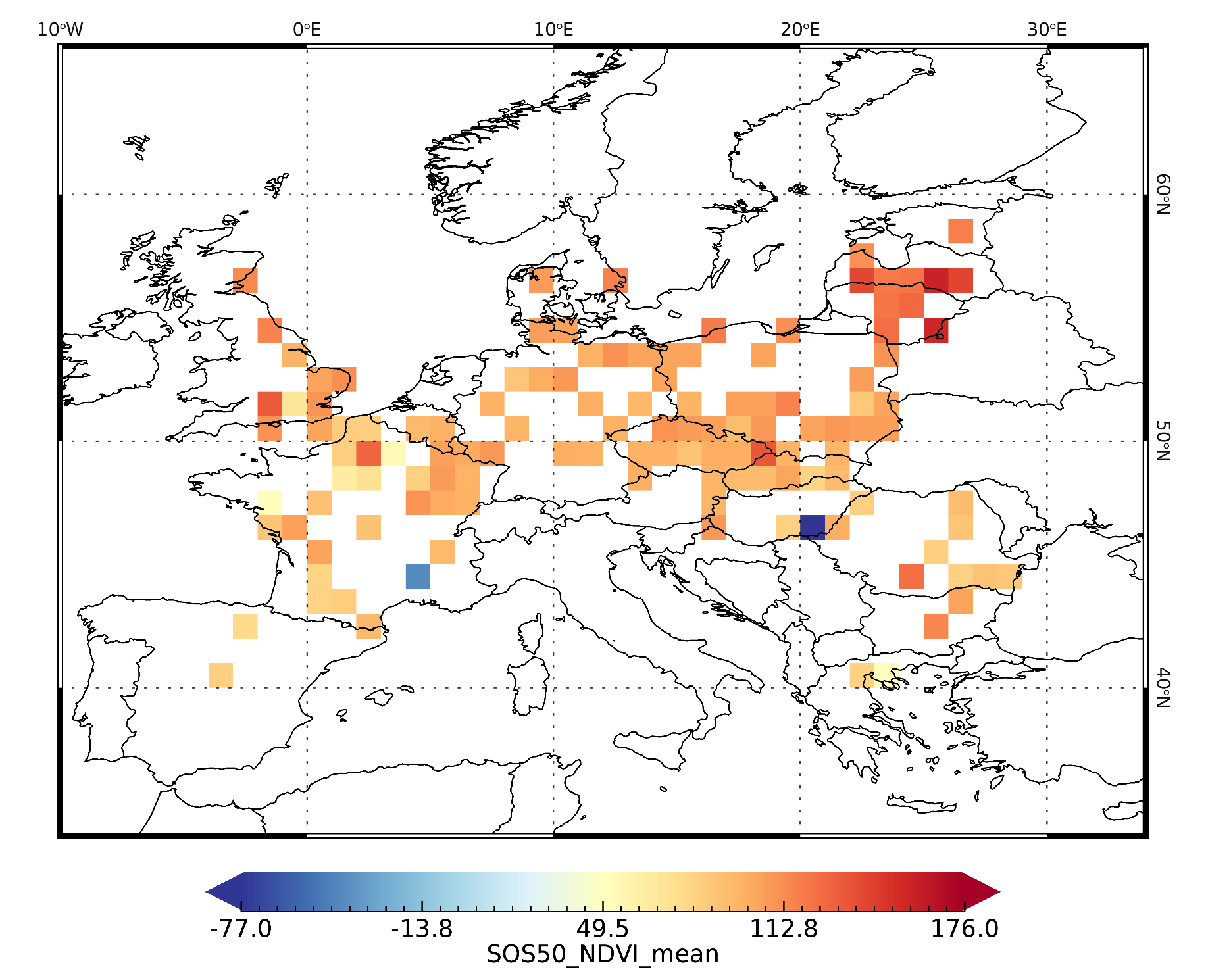** | **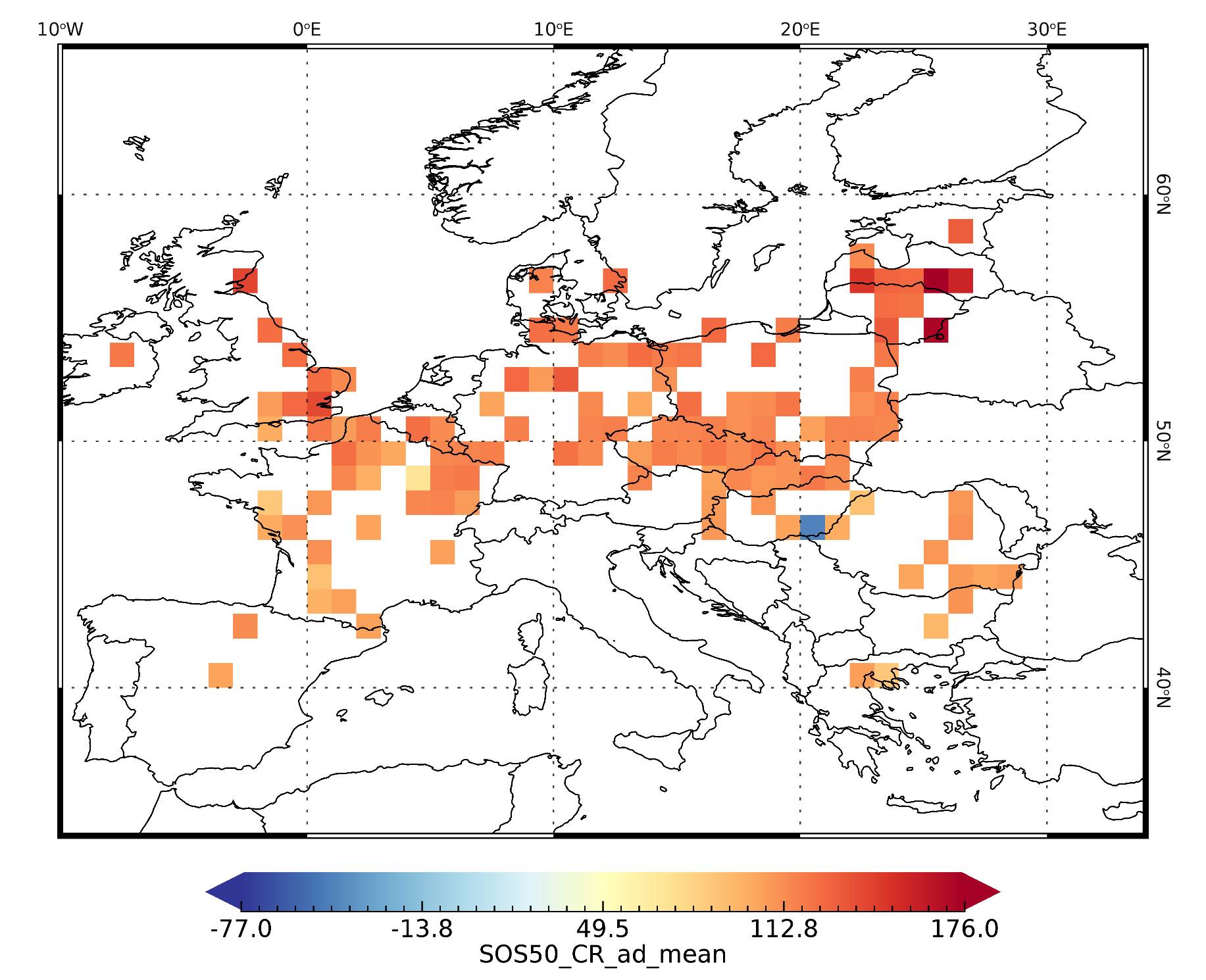** |
| **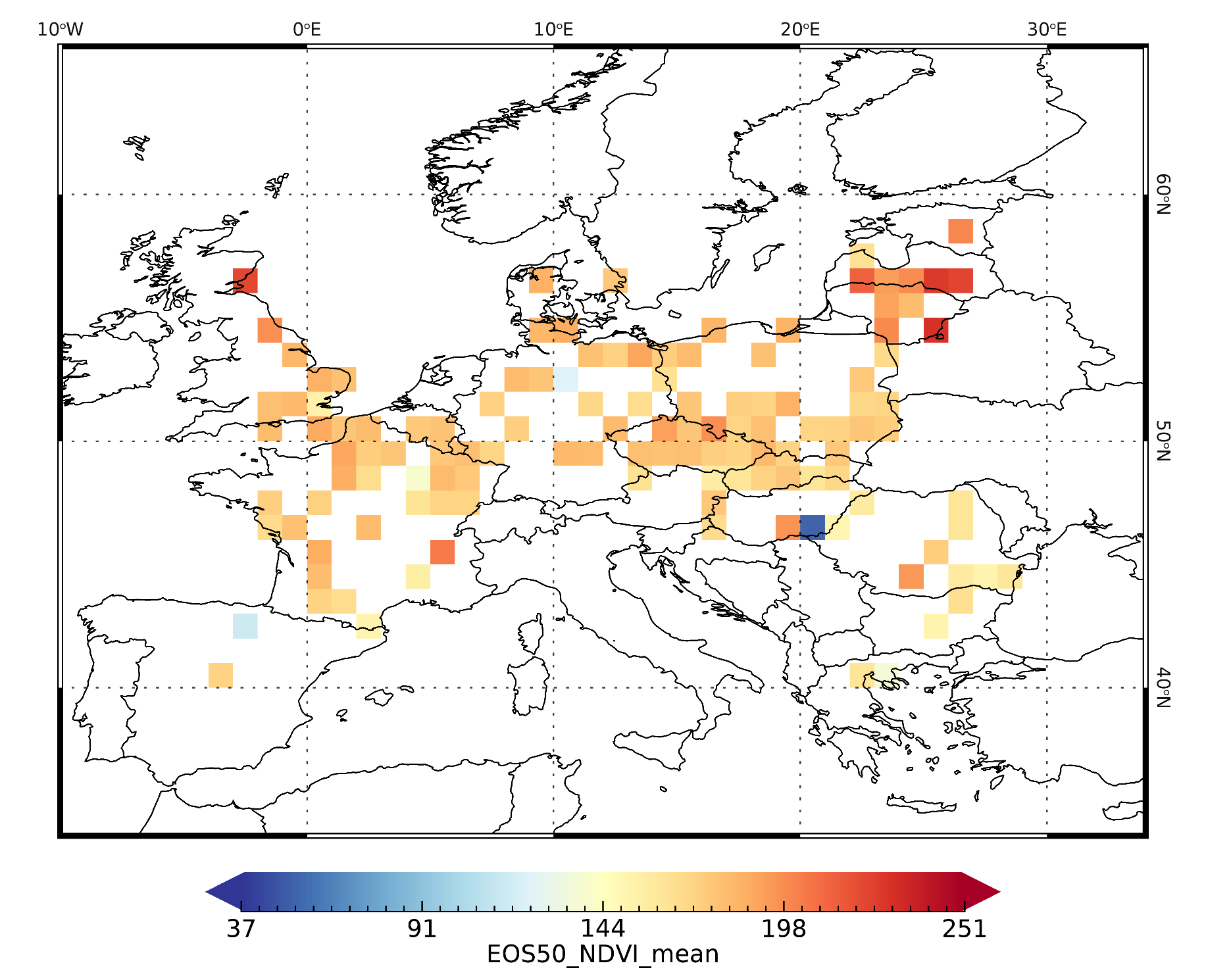** | **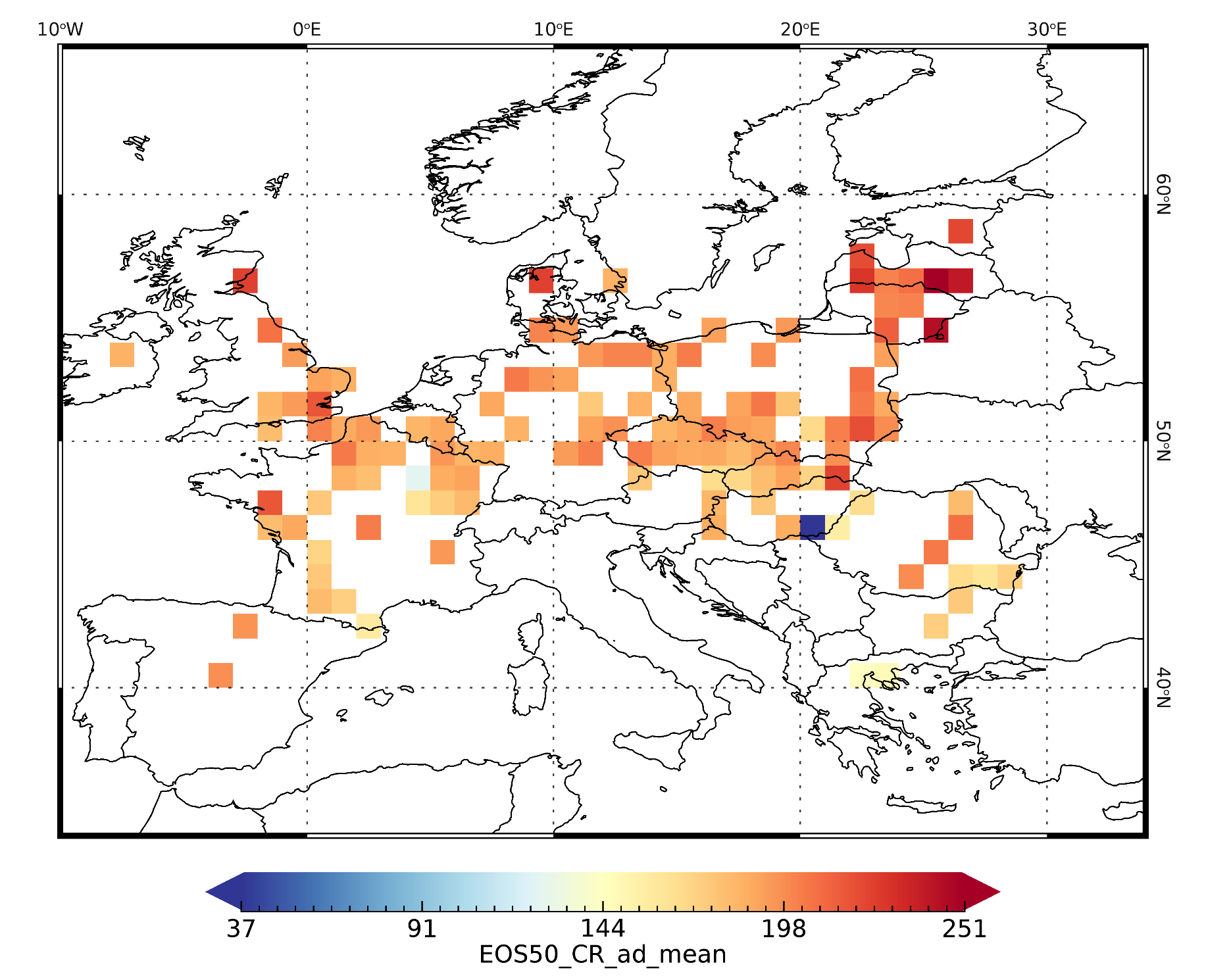** |
| **Oats** | |
| **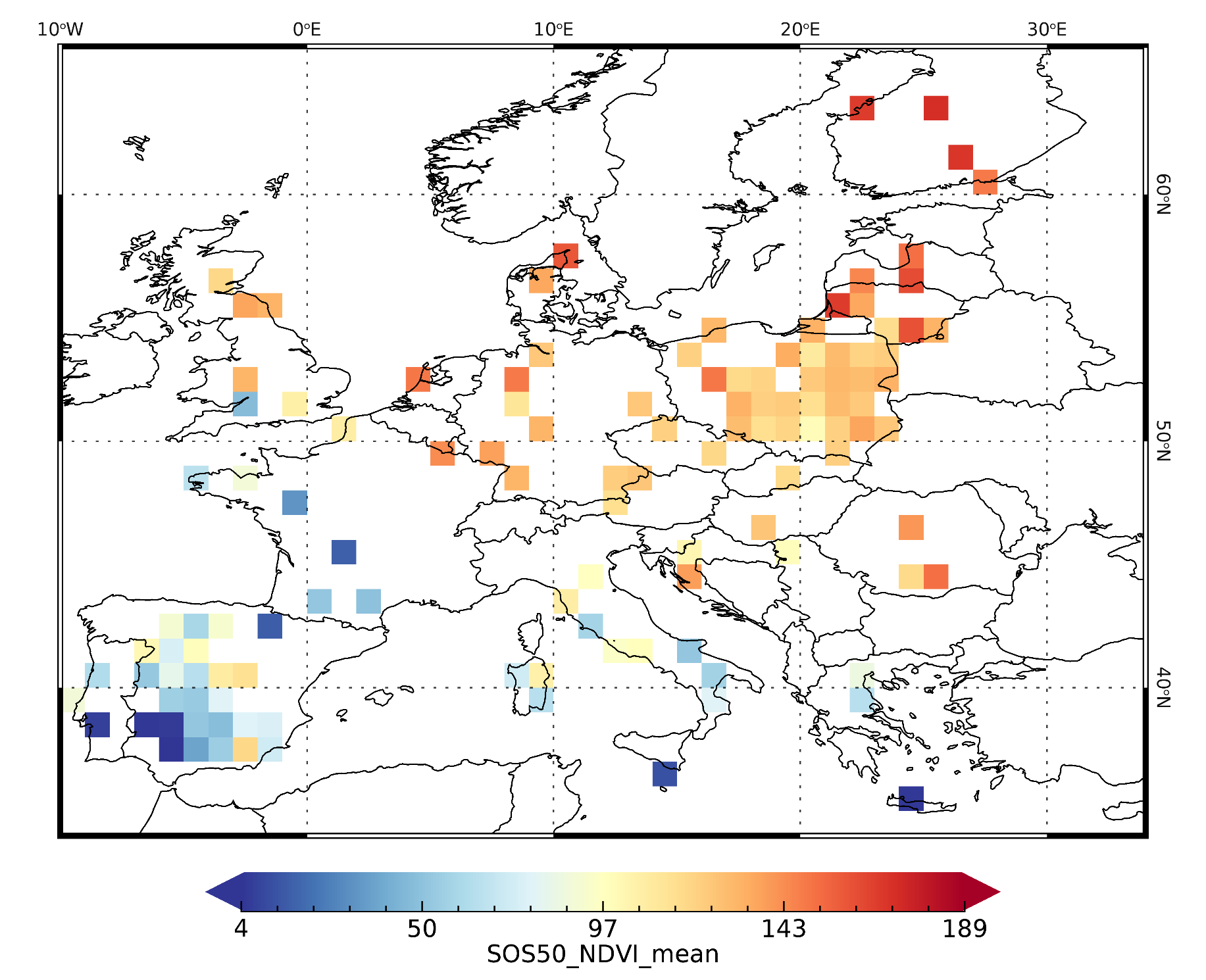** | **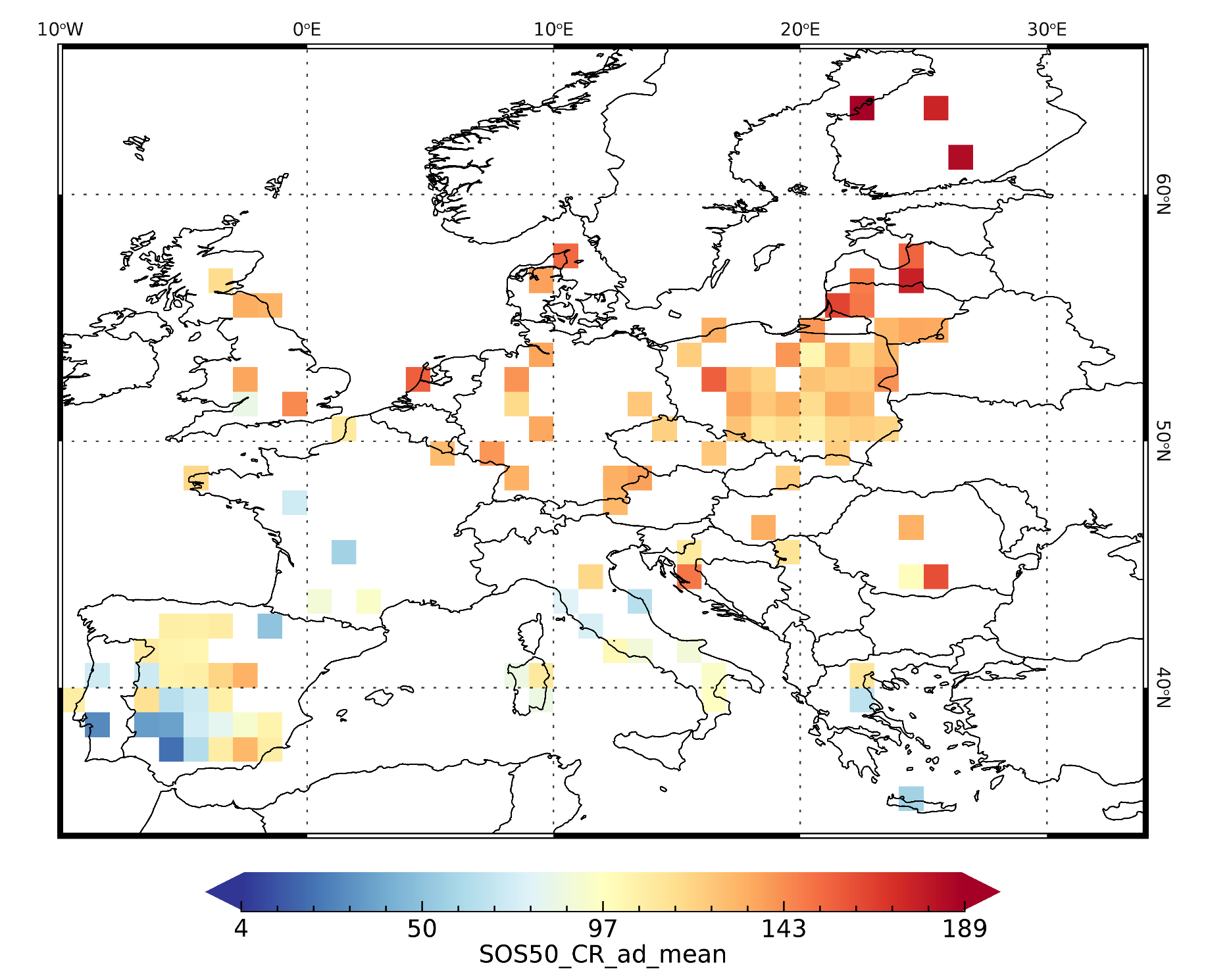** |
| **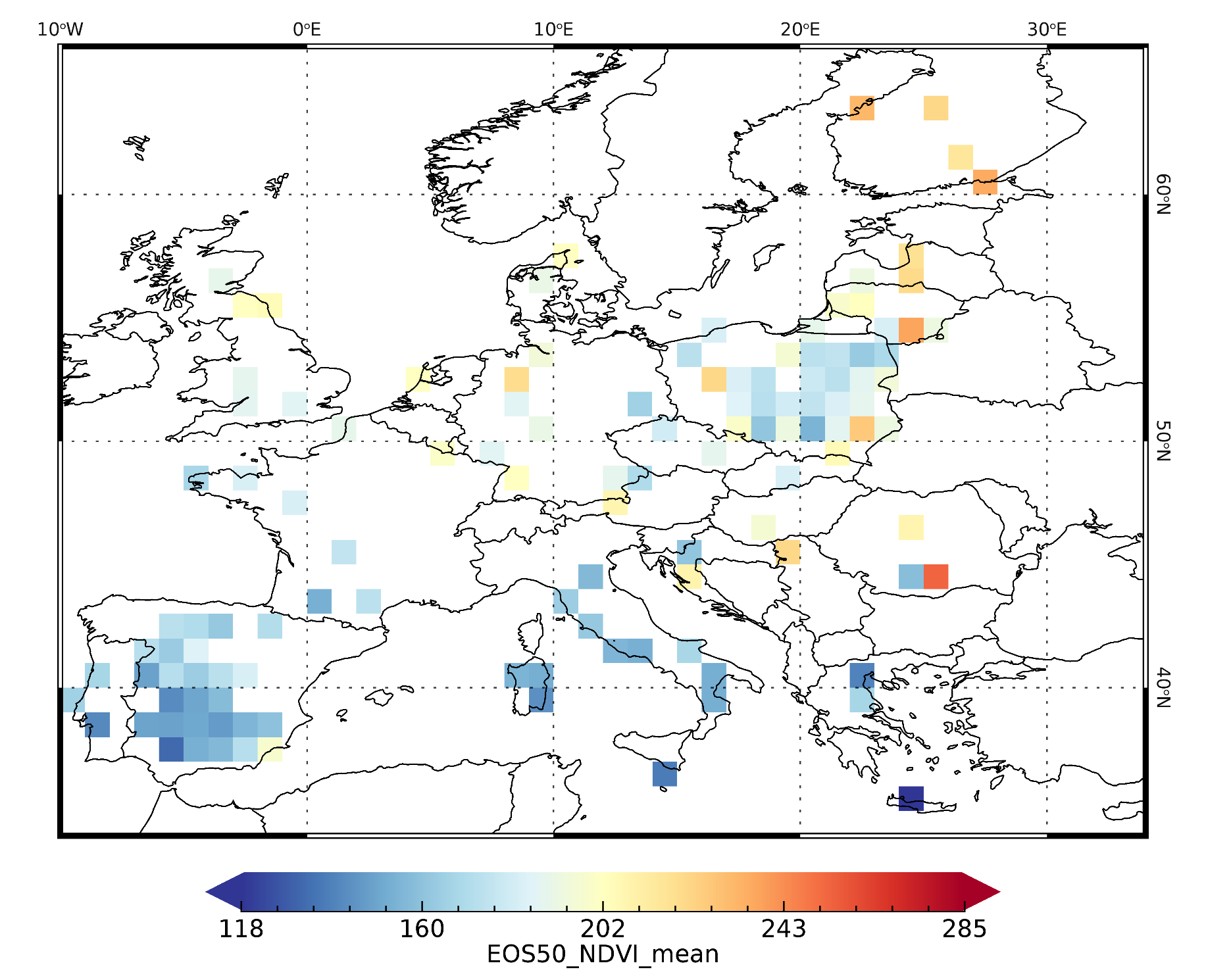** | **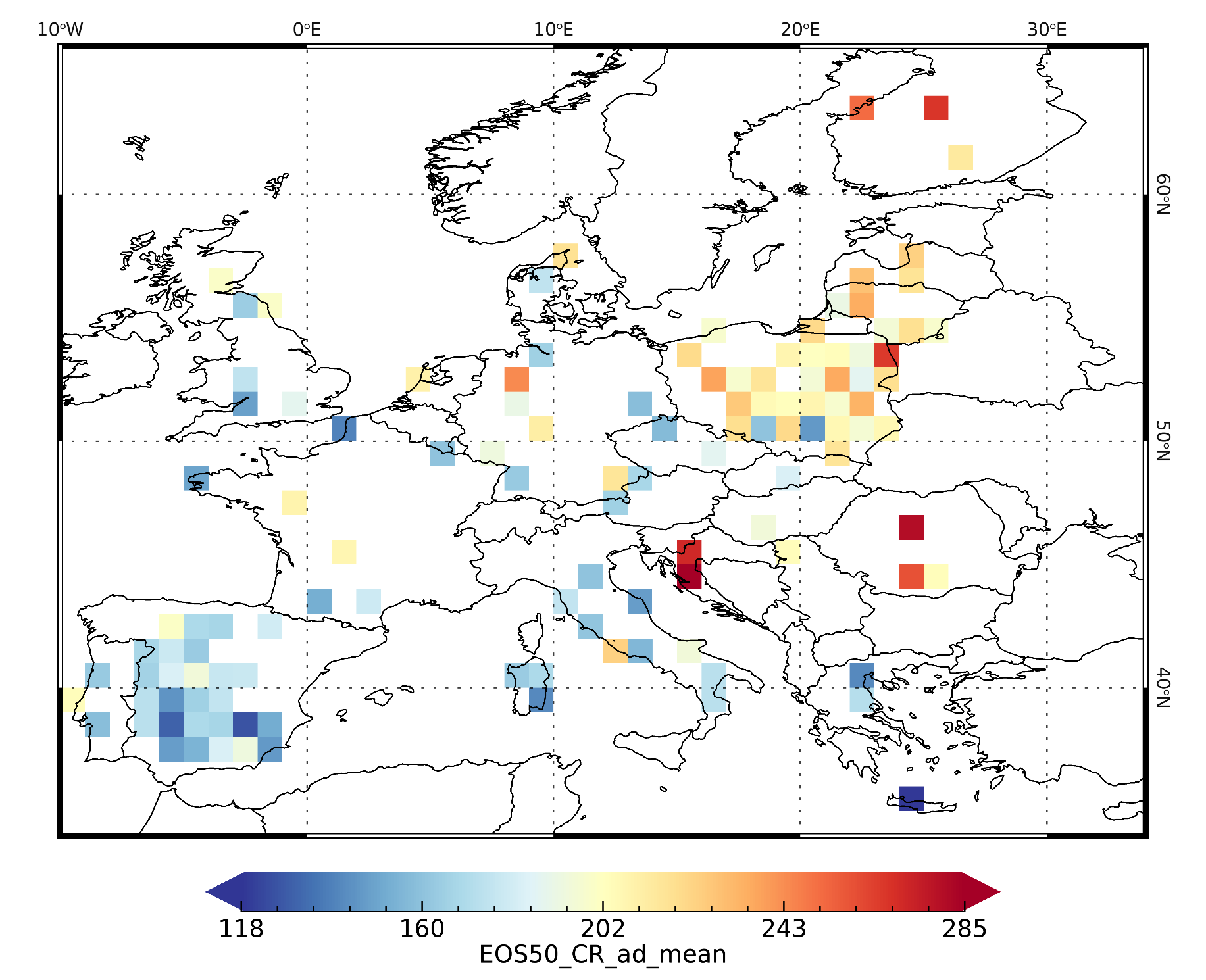** |
| **Sugar beet** | |
| **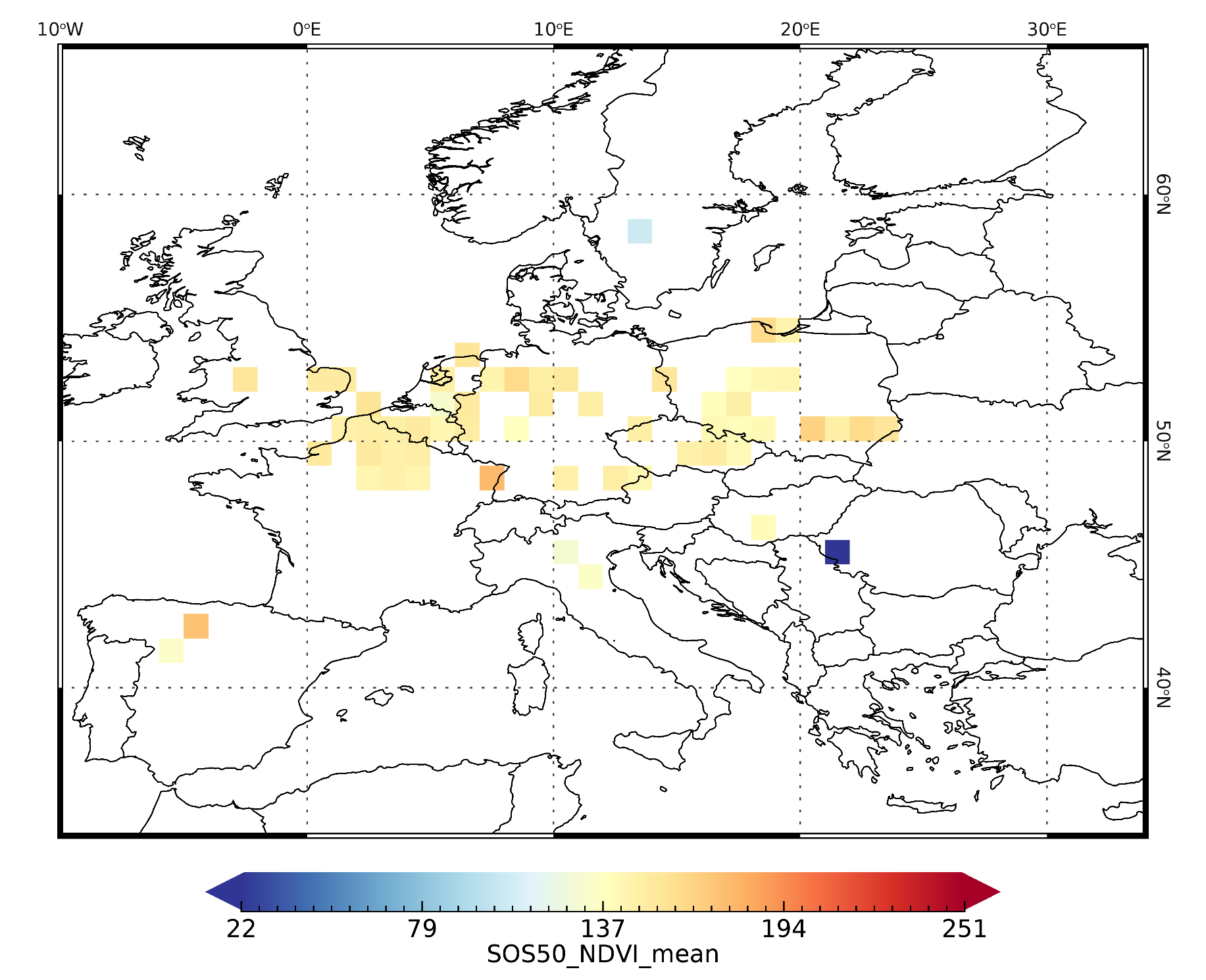** | **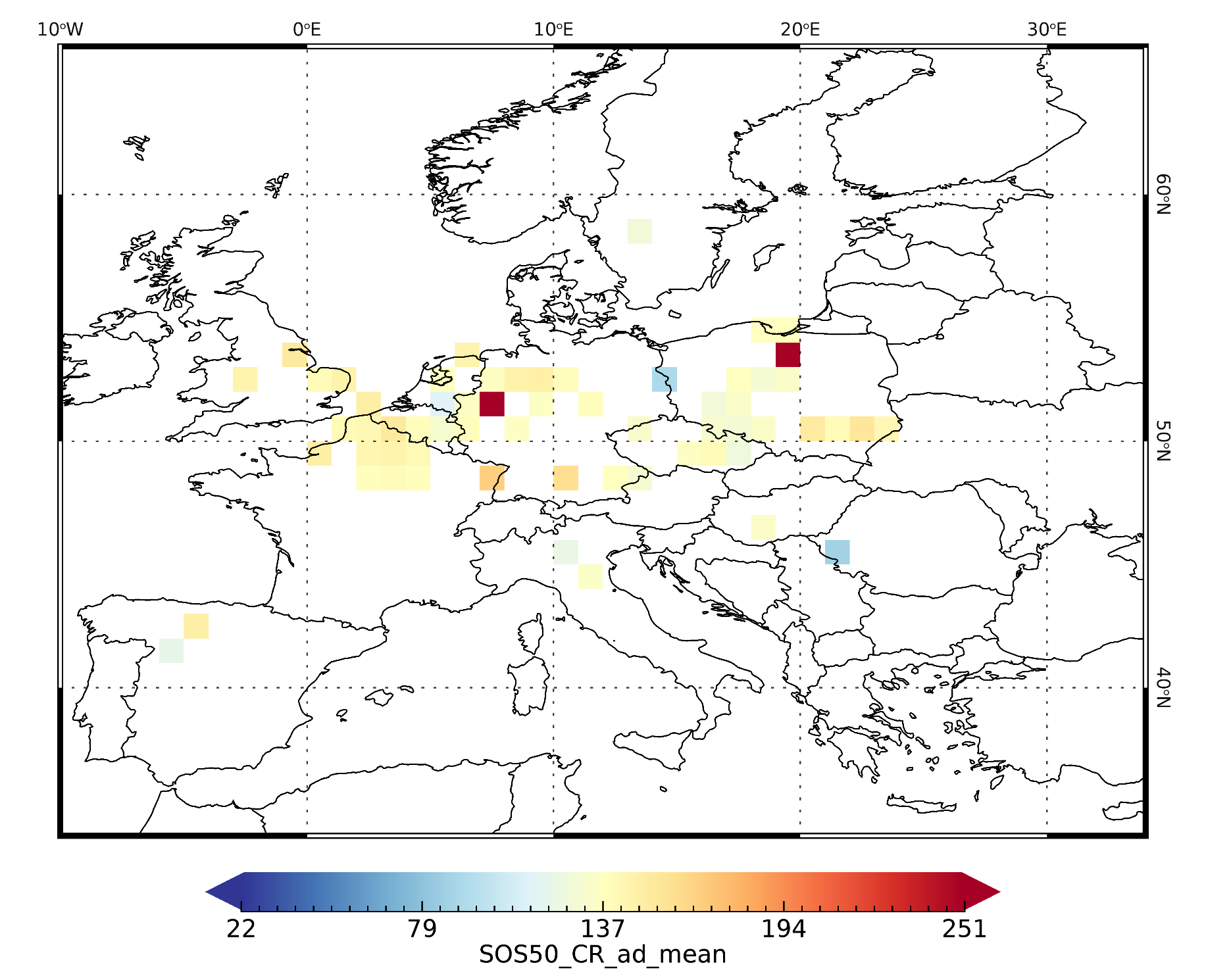** |
| **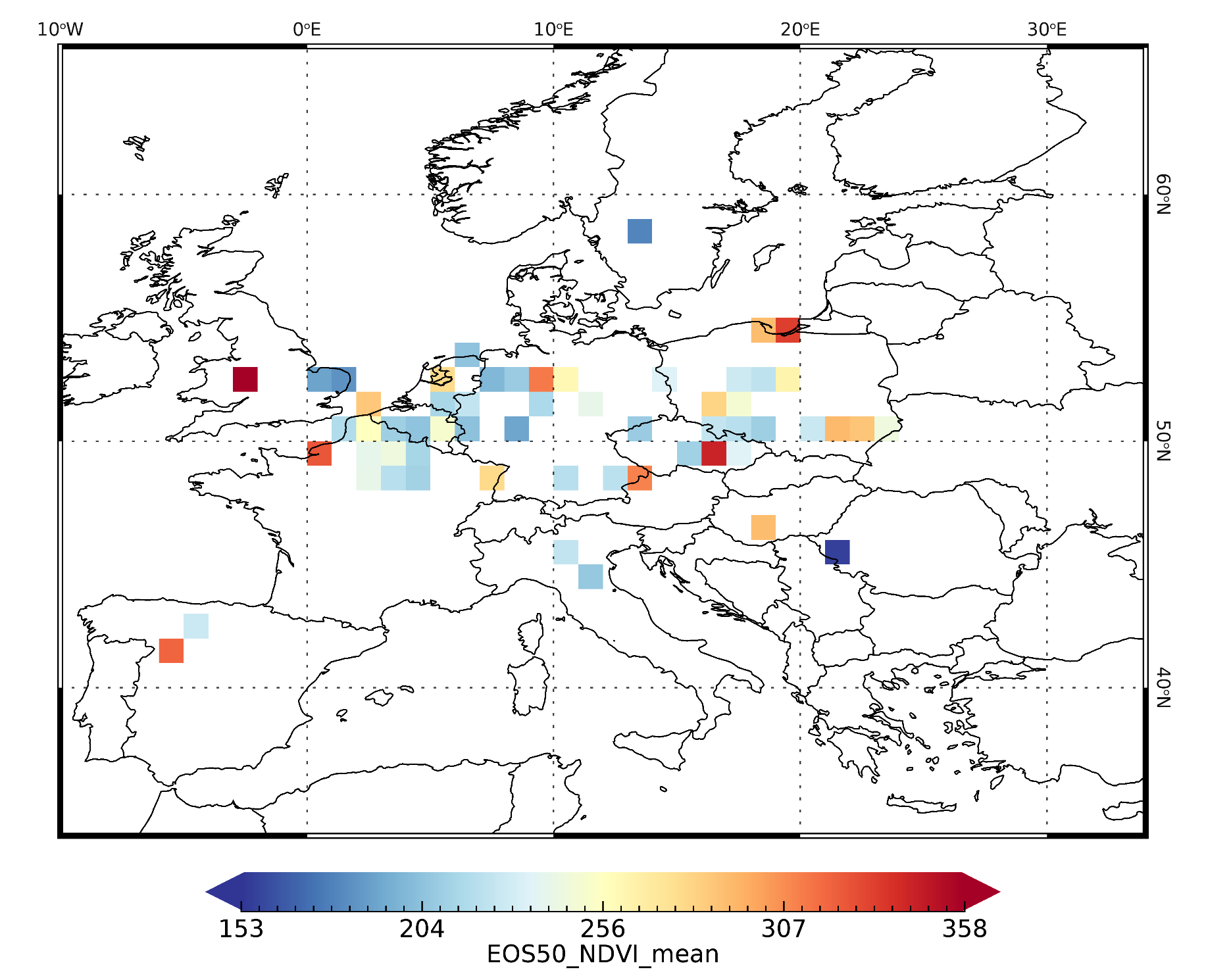** | **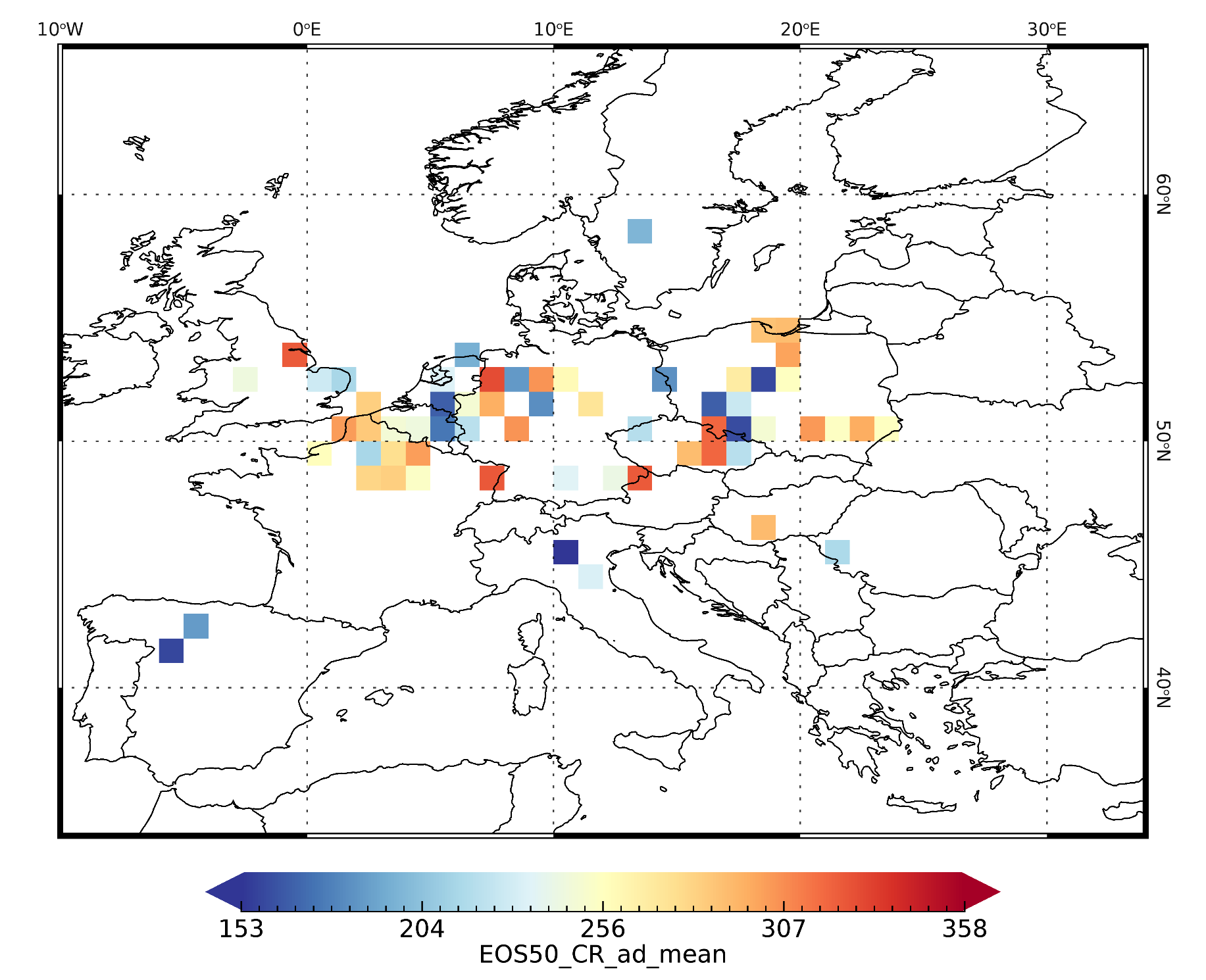** |
| **Sunflower** | |
| **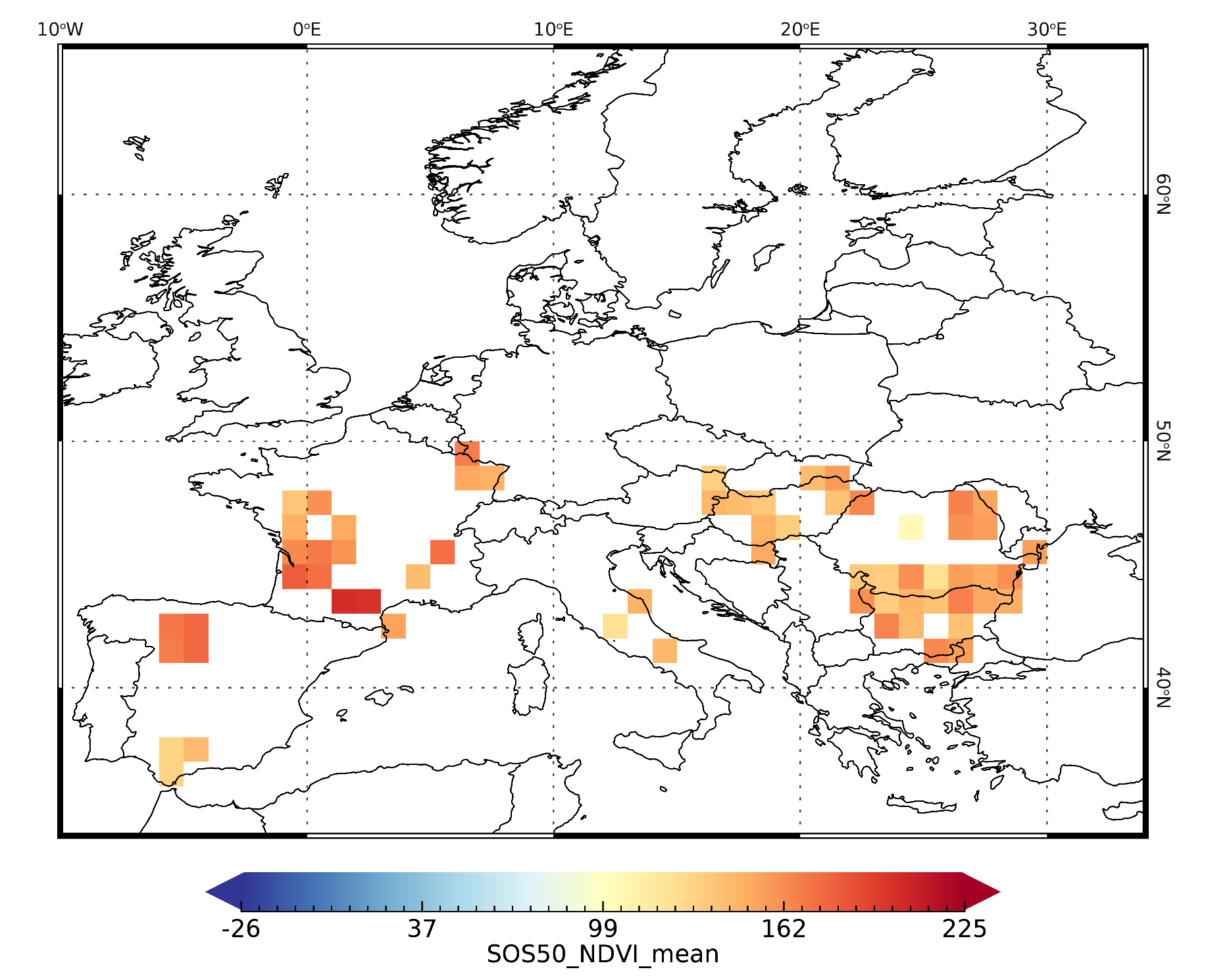** | **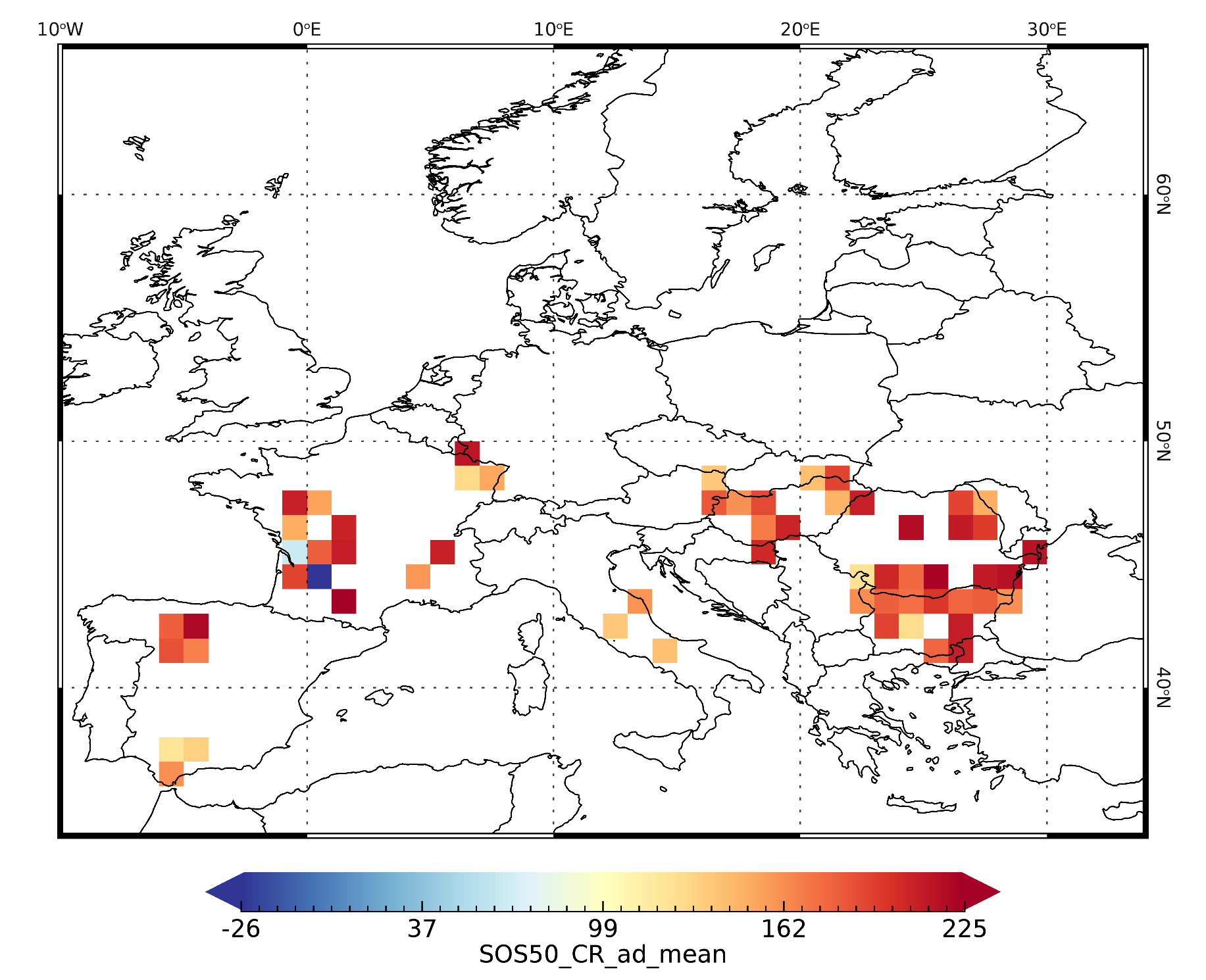** |
| **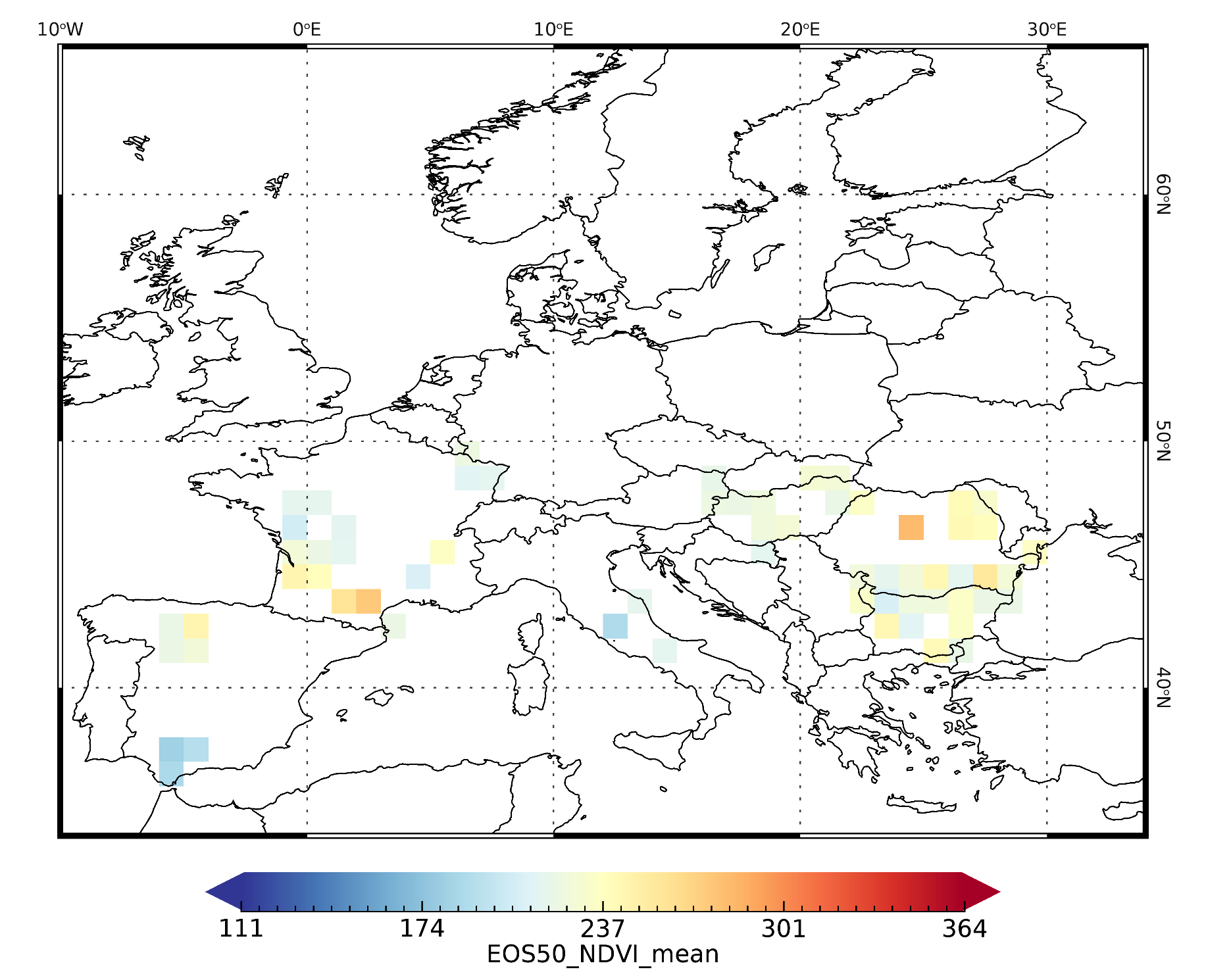** | **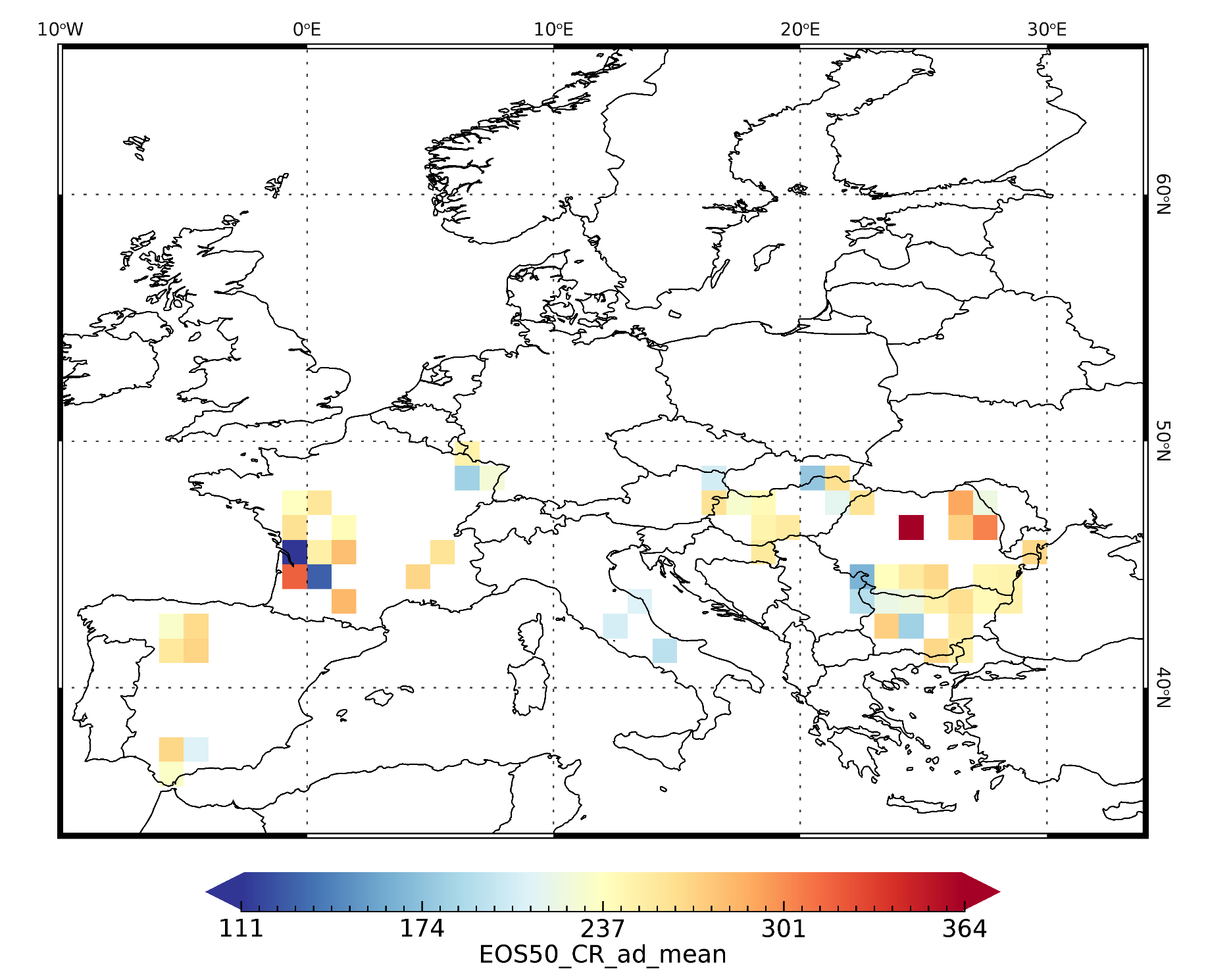** |
| **Dry pulses** | |
| 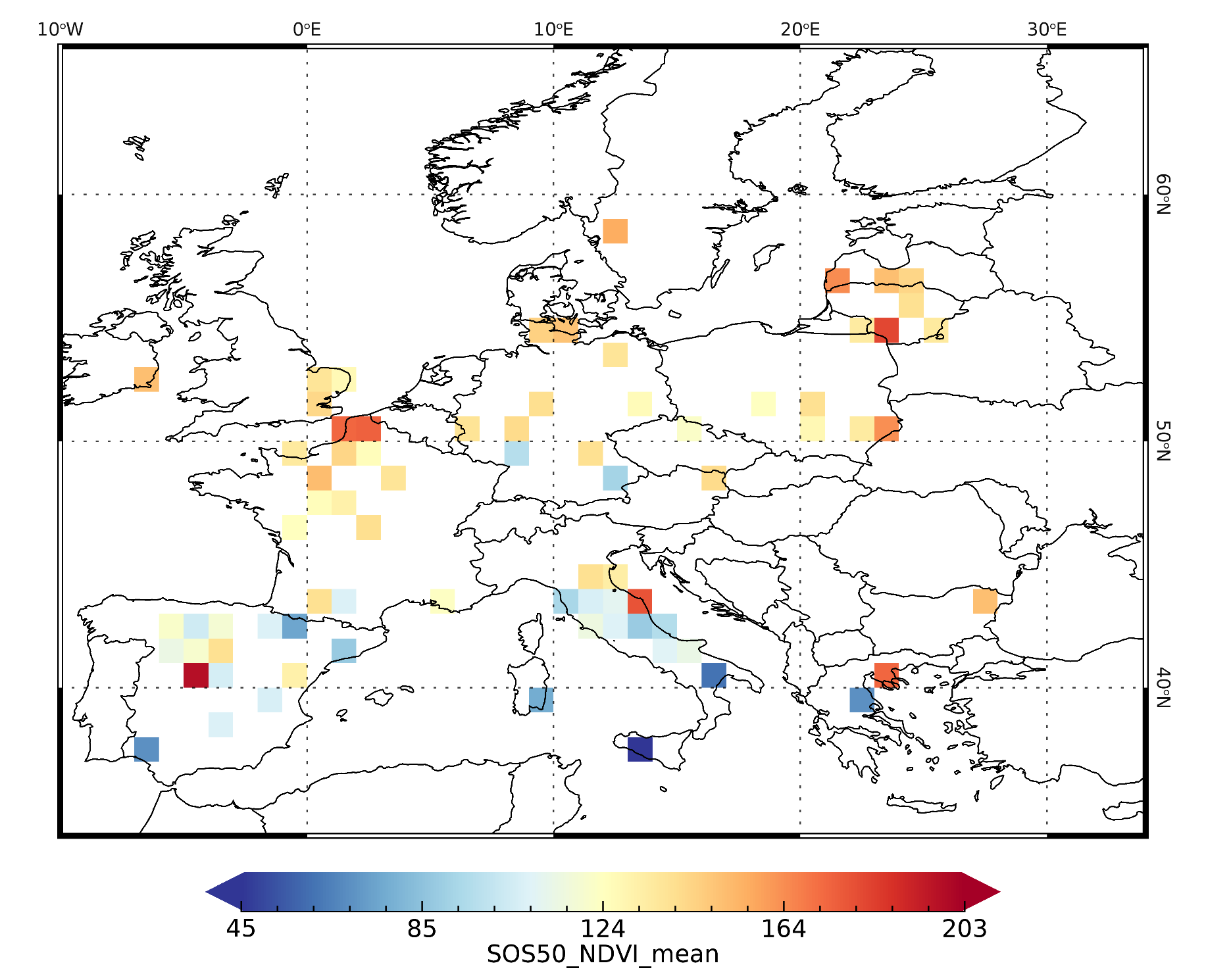 | 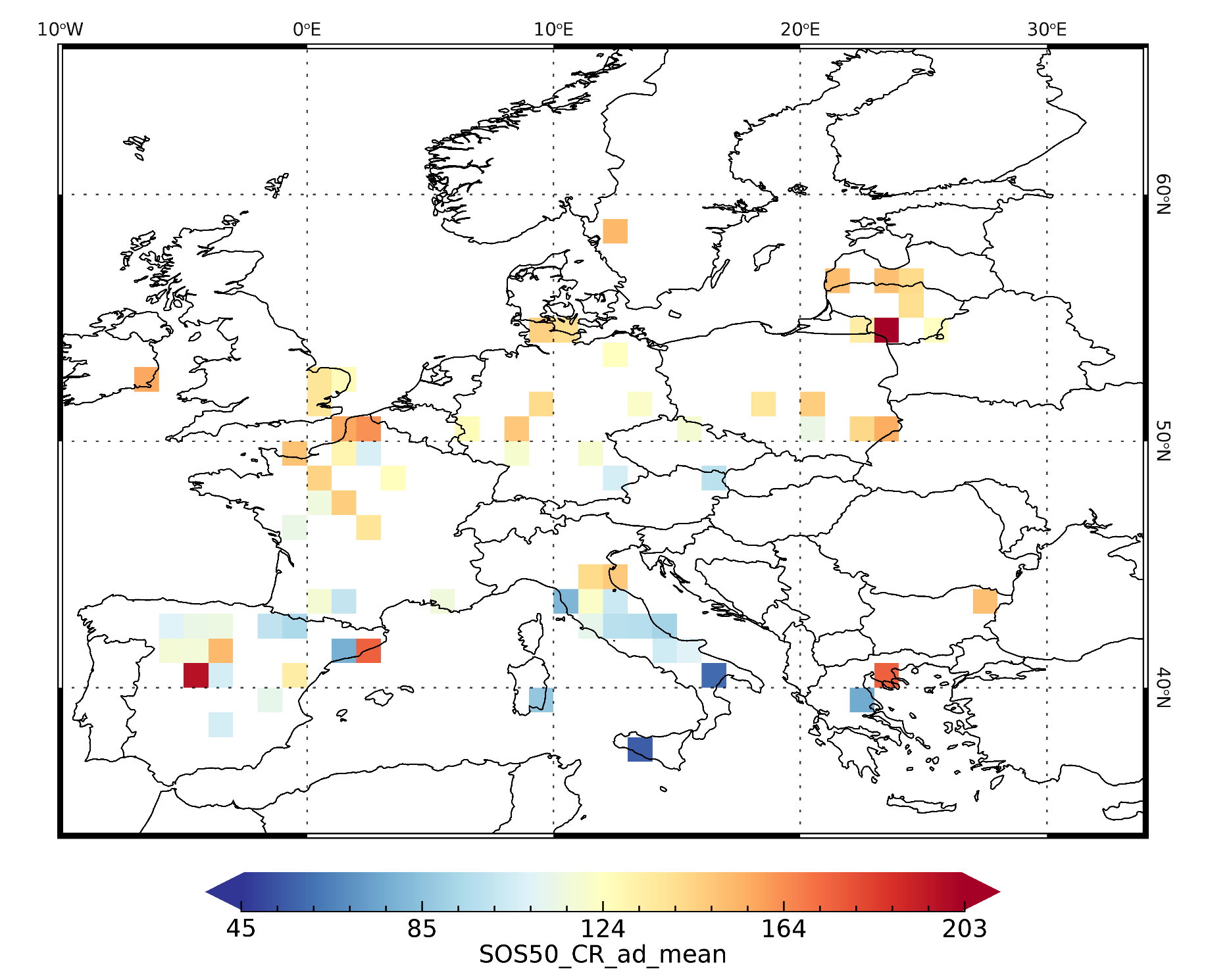 |
| 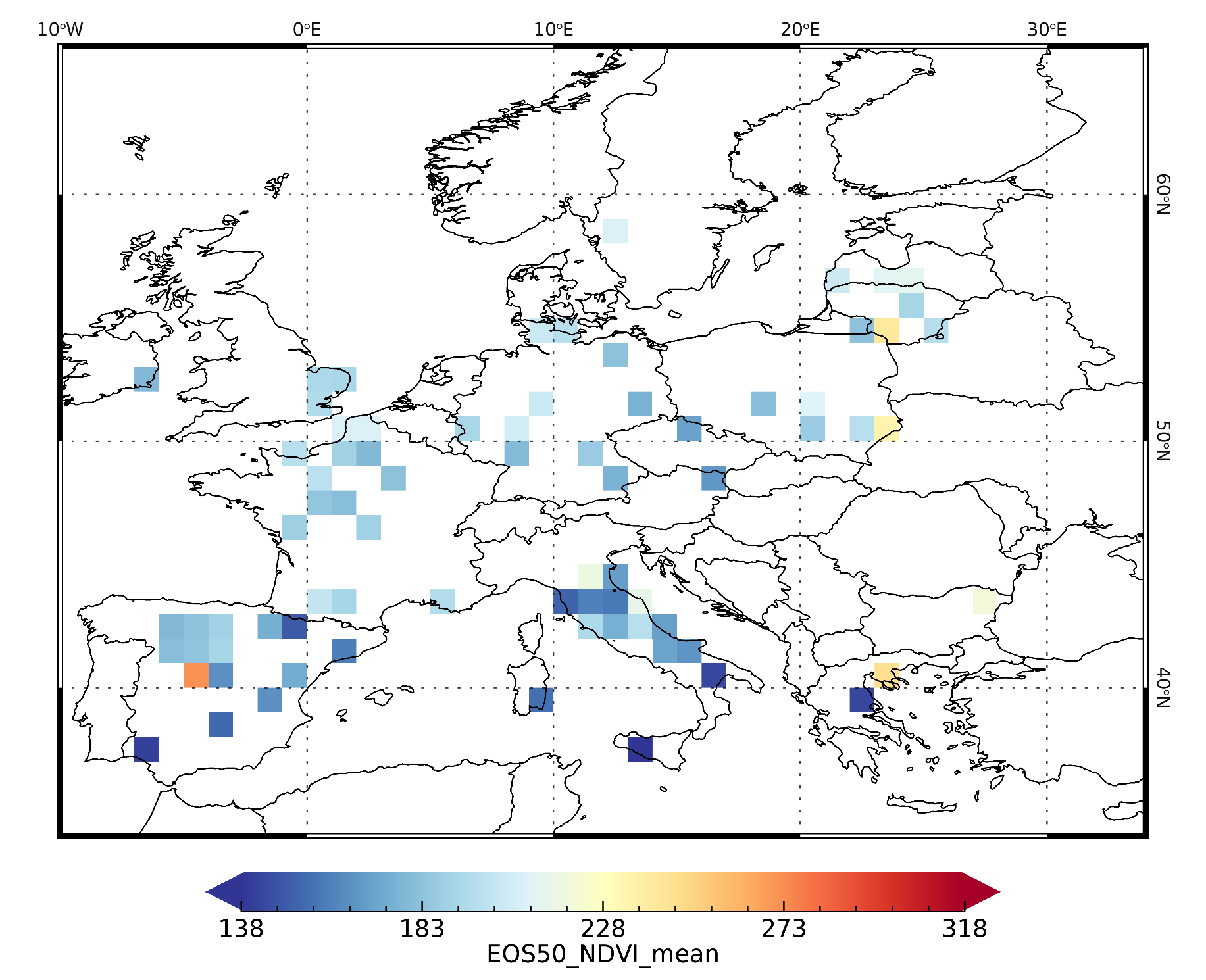 | 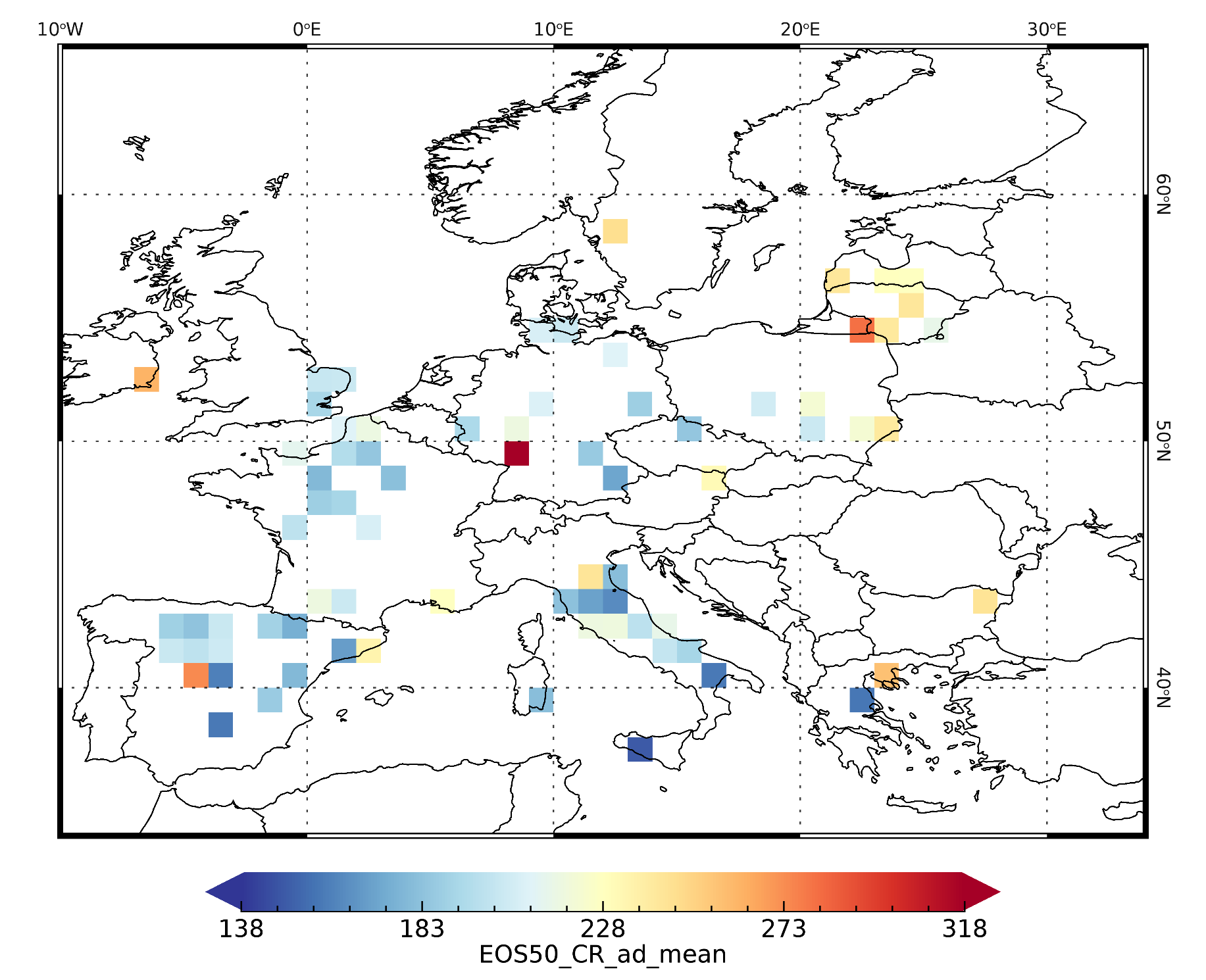 |

*Fig. S5. EU-level maps of common wheat, maize, durum wheat, rape and turnip rape, oats, sugar beet, sunflower, and dry pulses. Barley is presented in the manuscript Fig. 7. For each crop type, four maps are presented: top-left one depicts SOS50 derived from NDVI, top-right one depicts SOS50 derived from CR, bottom-left one depicts EOS50 derived from NDVI, and bottom-right one depicts EOS50 derived from CR. Maps were obtained by averaging the phenological timings of the polygons available within the cells of a 1 degree grid.*

| **A)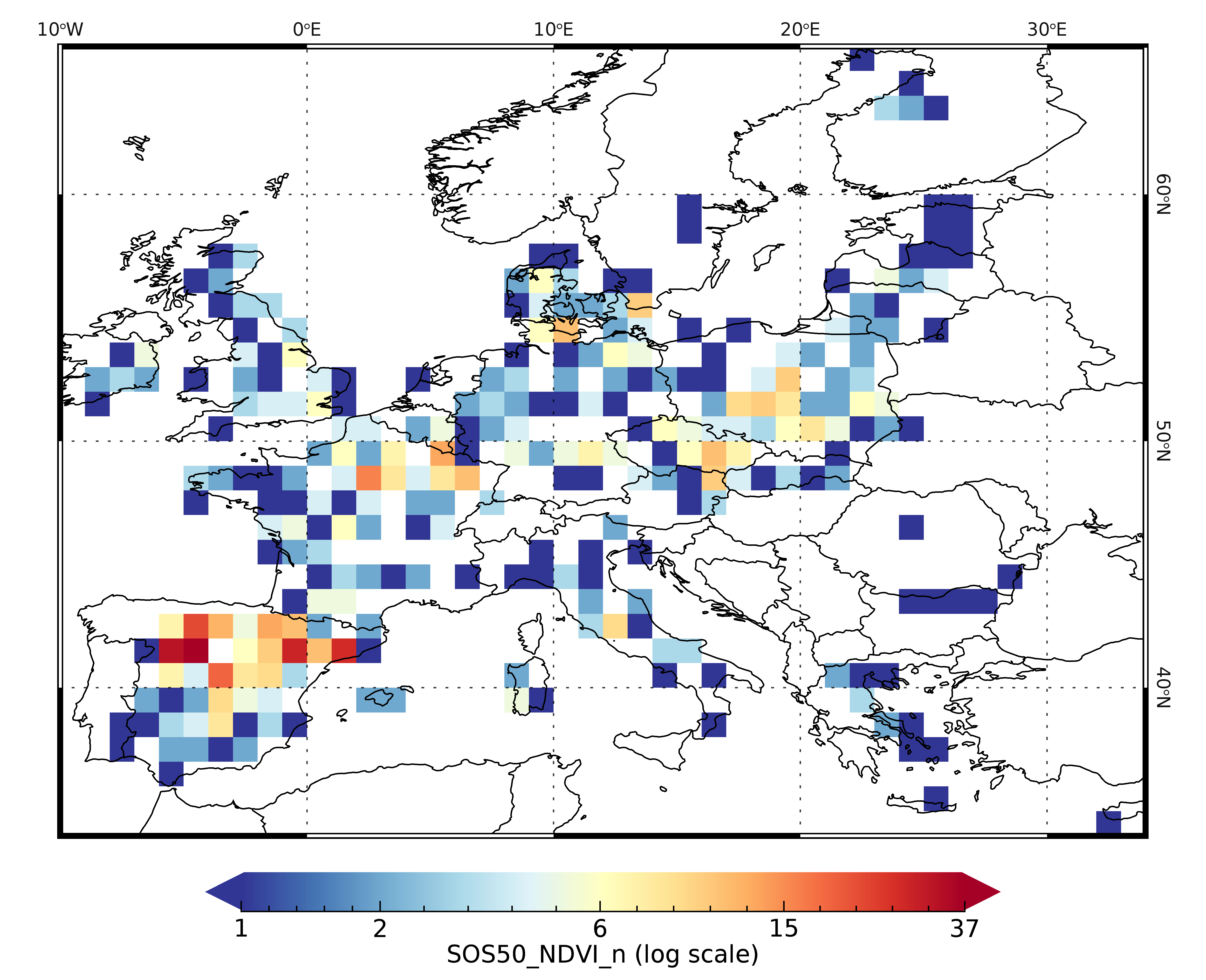** | **B)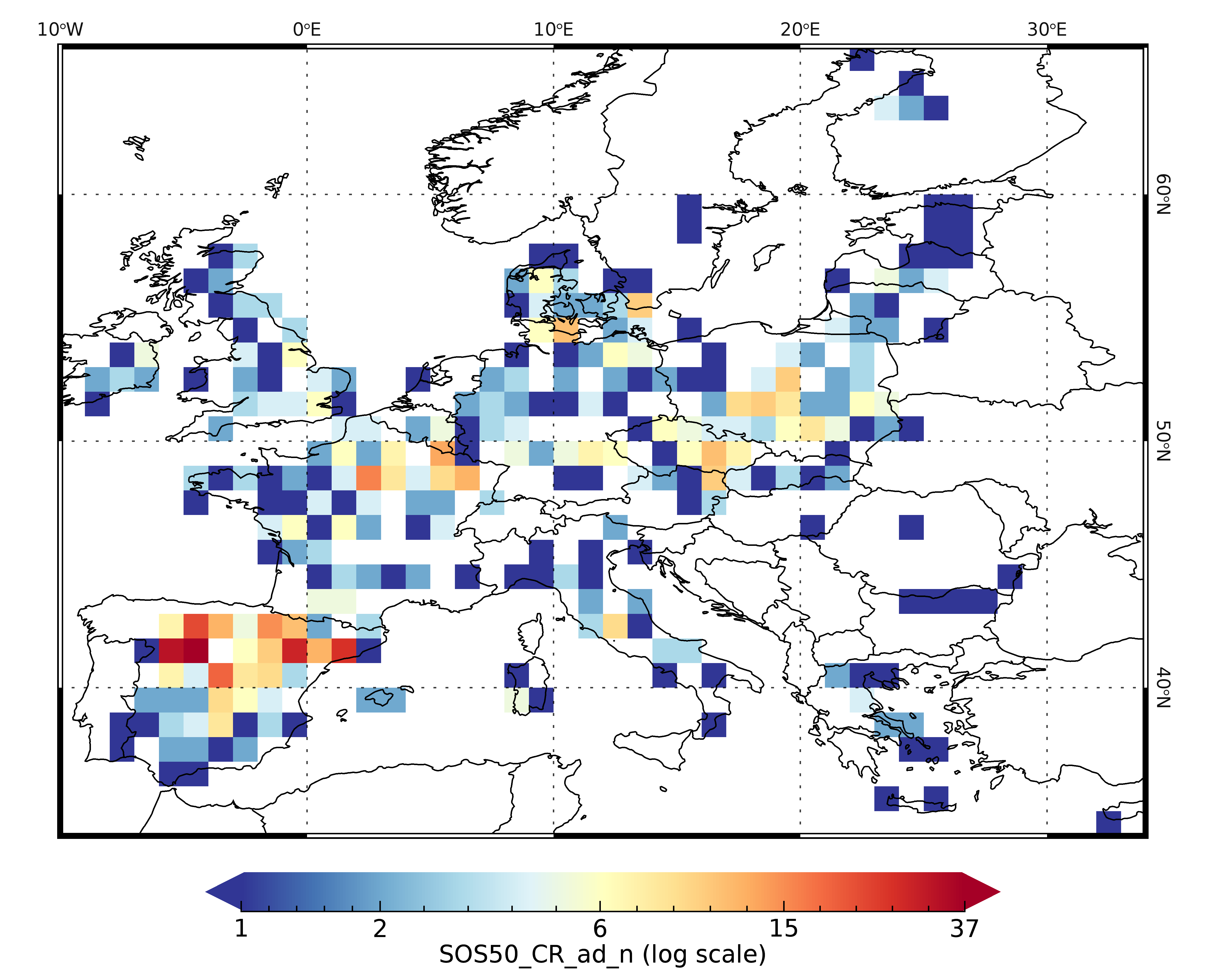** |
| --- | --- |

*Fig. S6. Number of successful barley phenology retrievals per 1-deg grid cell using NDVI (A) and CR (B).*

| 1. **All orbits (ascending and descending)**   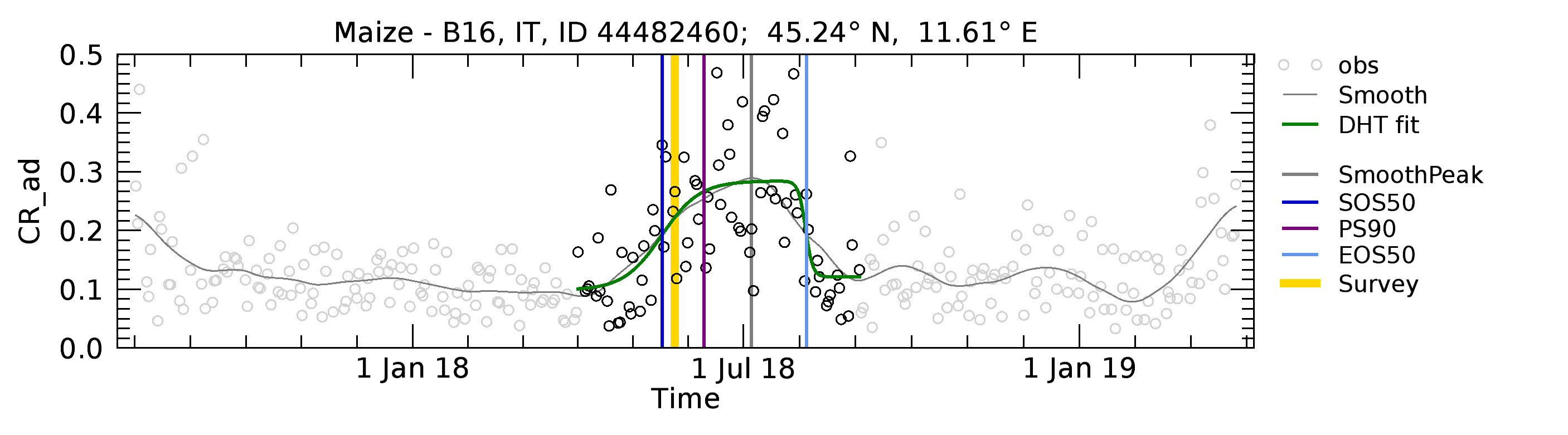 |
| --- |
| 1. **Ascending, relative orbit 117**   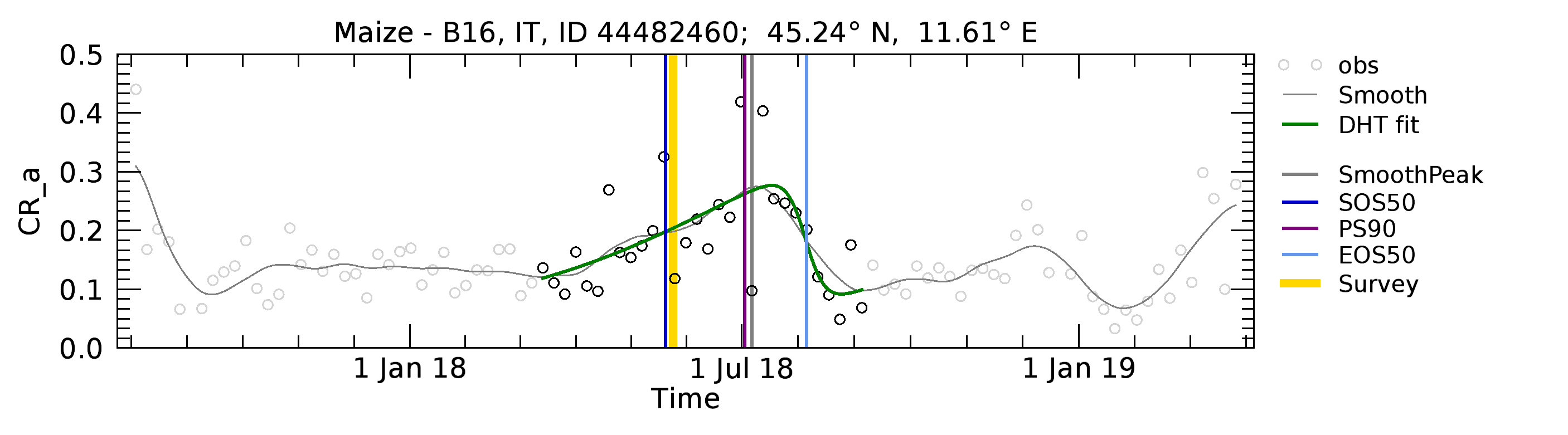 |
| 1. **Descending, relative orbit 95**   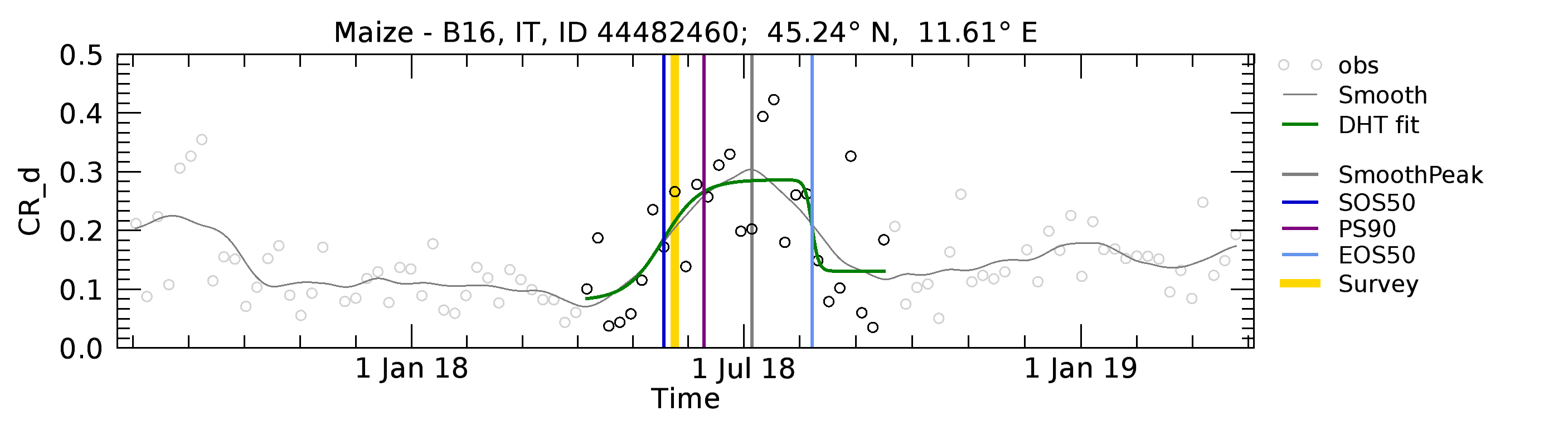 |
| 1. **Descending, relative orbit 168**   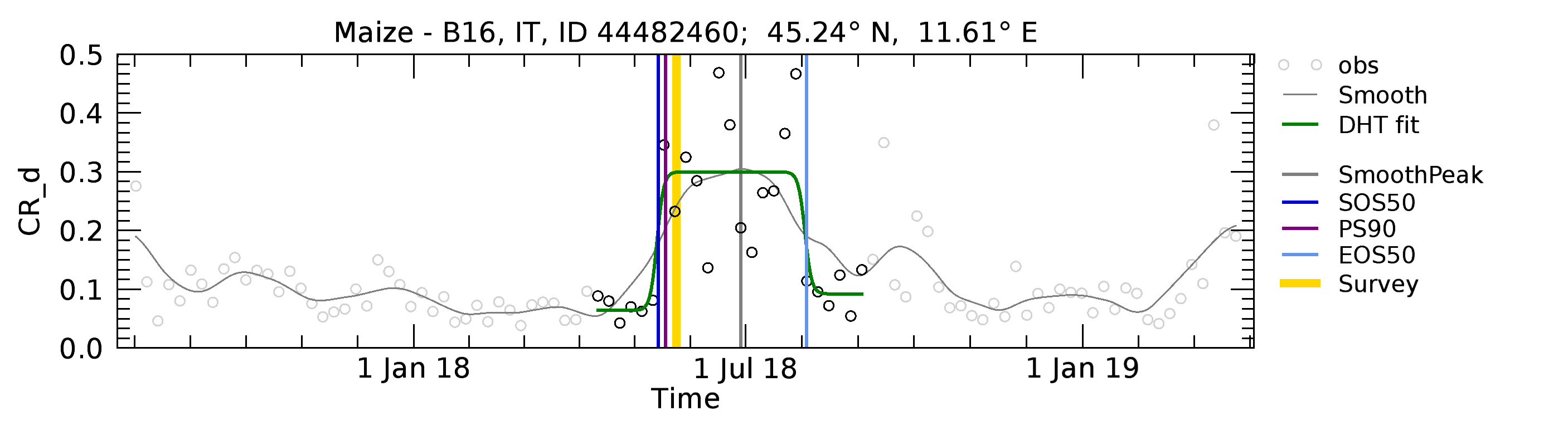 |

*Fig. S7. The data points of the temporal profile of Fig. 5B (reported in A) are separated in the available relative orbits (ascending orbit 117 in B, descending orbit 95 in C, descending orbit 168 in D) that are treated separately by the LSP retrieval algorithm.*
